# ﻿Towards a revision of the Palaearctic species of *Aphodius* Hellwig, 1798, subgenus *Liothorax* Motschulsky, 1860 (Coleoptera, Scarabaeidae, Aphodiinae)

**DOI:** 10.3897/zookeys.1207.117225

**Published:** 2024-07-22

**Authors:** Robert B. Angus, Jason F. Maté, Elizabeth M. Angus, David Král

**Affiliations:** 1 Department of Life Sciences (Insects), The Natural History Museum, Cromwell Road, London SW7 5BD, UK The Natural History Museum London United Kingdom; 2 c/Henares 16, Velilla de San Antonio, Madrid, 28891, Spain Unaffiliated Madrid Spain; 3 Biomedical Imaging Unit, Level B South Block, Mail point 12, General Hospital, Southampton SO16 6YD, UK Mail point 12, General Hospital Southampton United Kingdom; 4 Department of Zoology, Faculty of Science, Charles University, Viničná 7, CZ-128 00, Praha 2, Czech Republic Charles University Praha Czech Republic

**Keywords:** Chromosomes, distribution, habitats, morphology, mtDNA, new species, Scarabaeidae, taxonomy

## Abstract

The Palaearctic species of Aphodius Hellwig, 1798, subgenus Liothorax Motschulsky, 1860 are revised using a combination of chromosome analysis, molecular phylogenetics and morphological statistical analysis. Sixteen species are recognised, one of which is shown to comprise two subspecies. Based mainly on the morphology of the aedeagal endophallus and the phylogenetic analysis, they are placed in two groups: the. *niger* group, apparently monophyletic, comprising Aphodius (Liothorax) niger Illiger, 1798, A. (L.) muscorum (Ádám, 1994), **stat. rest.**, A. (L.) felix**sp. nov.**, A. (L.) bellumgerens**sp. nov.**, A. (L.) bameuli**sp. nov.**, A. (L.) krelli**sp. nov.**, A. (L.) isikdagensis (Balthasar, 1953), A. (L.) alberti**sp. nov.** and A. (L.) wilsonae Maté et Angus, 2005, **stat. rest.**; and the *plagiatus* group, almost certainly paraphyletic, comprising A. (L.) plagiatus (Linnaeus, 1767), including A. (L.) p. plagiatus and A. (L.) p. sinoplagiatus**subsp. nov.**, A. (L.) rodrigoi**sp. nov.**, A. (L.) discoidesA. Schmidt, 1916, **stat. rest.**, A. (L.) rutilipennis (Baudi di Selve, 1870), **stat. rest.**, A. (L.) chellala**sp. nov.**, A. (L.) kraatzi Harold, 1868, and A. (L.) rusakovi Gusakov, 2004. A key to the species is given as well as details of their morphology, distributions, and habitats.

## ﻿Introduction

Aphodius Hellwig, 1798 subgenus Liothorax Motschulsky, 1860 species are elongate parallel-sided beetles, to some extent hemicylindrical in shape though occasionally less elongate and more rounded. The head nearly always lacks any trace of tubercles on the frontoclypeal suture, the anterior margin of the clypeus is emarginate over its median third and the lateral angles of this emargination are bluntly rounded. The lateral area of the frons, between the frontoclypeal suture and the eyes, bulges outwards as a pair of rounded genae whose outer margin may be either a simple continuation of that of the clypeus or distinctly angled outwards. The adult beetles typically inhabit mud at the edges of pools and epipharynx is of the type mainly adapted for saprophagy. Only very rarely do the beetles inhabit and eat dung.

At present five species of *Liothorax* are listed from the Palaearctic (Dellacasa et al. 2016), and a further five from the Nearctic (Dellacasa et al. 2007). No species is recorded as Holarctic.

In recent years, a large amount of chromosomal, molecular, and morphological data, not congruent with the current classification of the Palaearctic members of the group, has been accumulated by the authors, necessitating a taxonomic revision. The present revision is based on a combination of different methodologies – morphology, morphometrics, molecular phylogenetics and chromosomal studies – a rare example of truly integrative taxonomy.

The initial motivation of the present work stems from total disbelief that the distinctive features of Aphodius (Liothorax) wilsonae Maté & Angus were merely traits of intraspecific variation, as claimed by Dellacasa and Dellacasa (2005). In 2005 Maté and Angus (2005) described a new species of *Liothorax* as Aphodius (Liothorax) wilsonae, clearly related to *L.niger* Illiger, 1798 but differing significantly from it on several aspects, not just on external morphology but also in its chromosomes, mtDNA, and in the morphology of the aedeagus and particularly the endophallus, with strikingly smaller endophallic teeth.

Maté and Angus (2005) regarded *A.wilsonae* as widely distributed in southern Europe and extending into Asia as far as Iran, although they noticed that the specimens from outside the Iberian Peninsula were quite different, and therefore all material outside Iberia was left out of the type series pending additional data. Furthermore, the authors also lacked sufficient *A.niger*-like material from much of its range in Central and Eastern Europe, and as such left these specimens labelled as A.cf.niger. In spite of these limitations, the evidence for *niger* and *wilsonae* being distinct species was highly supportive. Nevertheless, and in spite of this suite of characters, Dellacasa and Dellacasa (2005) placed *A.wilsonae* as a synonym of A. (L.) niger, stating that the characters used to characterise the new species were the traits of intraspecific variation. Furthermore, in their 2007 review of *Liothorax*, Dellacasa et al. (2007) maintained this view, and it persists in Volume 3 of the revised edition of the Catalogue of Palaearctic Coleoptera (Dellacasa et al. 2016).

Since Dellacasa and Dellacasa (2005), Dellacasa et al. (2007) provided neither data nor a detailed explanation of why they considered characters of *A.wilsonae* to be within intraspecific variation of *A.niger*, we can only speculate that it was based solely on gross (dorsal) morphological, aedeagal, and epipharyngeal characters (but not, surprisingly, the endophallus), whilst completely ignoring the morphological, karyological, and molecular evidence which was impossible to reconcile with both lineages being conspecific. In order to counter the claim that the character variation was mere “intraspecific variation” it was necessary to examine a much larger sample of specimens. In April 2009 RBA collected a small sample of a black Aphodius (Liothorax) on the Campo Felice of the Abruzzo mountains of Italy and, assuming it to be *A.wilsonae*, remarked that we now had the species in Italy, so it would have to be taken more seriously (Angus 2010: 3). However, subsequent study of the chromosomes by RBA and of the DNA by JFM showed that the animal was not *L.wilsonae* but an undescribed species. In the light of this discovery, RBA and JFM embarked on a study of *L.niger*-like material from as wide an area as possible, and it is this project which lies at the heart of the current revision.

## ﻿Materials and methods

### ﻿Material

The museums from which material has been borrowed for study, and in which material has been deposited, are as follows:

The Linnean Society, Burlington House, London, UK (**LSCL**) (Susan Ryder); The Natural History Museum, London, UK (**NHMUK**) (Max Barclay); Oxford University Museum, Oxford, UK (**OUM**) (Darren Mann); Muséum d’histiore naturelle, Genève, Switzerland (**MNHG**) (Giulio Cuccodoro); Muséum national d'Histoire naturelle, Paris, France (**MNHN**) (Mlle Nicole Berti); Humboldt Museum, Berlin, Germany (**ZMB**) (Johannes Frisch); Zoologische Staatsammlung München, Germany (**ZSM**) (Michael Balke); Hungarian Museum of Natural History, Budapest, Hungary (**MNHB**) (Ottó Merkl); Museo Nacional de Ciencias Naturales, Madrid, Spain (**MNCN**) (Mercedes París); Naturhistorisches Museum, Wien (**NMW**) (Harald Schilhammer); ZZoological Institute, Russian Academy of Sciences St Petersburg, Russia (**ZIN**) (Andrey Frolov); Steinhardt Museum of Natural History, Tel Aviv, Israel (**IL-SMNH**) (Laibale Friedman); National Museum Prague, Czechia (**NMP**) (Jiří Hájek); Denver Museum of Nature and Science, Denver, Colorado, USA (**DMNS**) (Frank Krell); Alberto Ballerio Collection, Brescia, Italy (**AB**); Stefano Ziani Collection, Imola, Italy (**SZ**); Axel Bellmann Collection, Bremen, Germany (**ABC**); Carsten Zorn Collection, Gnoien, Germany (**CZC**); Jason Maté Collection, Madrid, Spain (**JFMC**).

Details of the material from which successful chromosome preparations have been obtained are given in Table 1 and of that sequenced for DNA, comprising ten ingroup and three outgroup species, are given in Table 2.

**Table 1.** Details of the material from which successful preparations have been obtained.

Species	Locality of origin	Specimens
*A.plagiatus*	England: Norfolk, Hunstanton 52.968°N, 0.525°E	1♂
	Dorset, Studland Heath 50.654°N, 1.955°W	3♂♂, 3♀♀
	China: Qinghai, Gangca 37.3°N, 100.183°E	1 ♂
*A.kraatzi*	Slovakia: Chl’aba 47.823°N, 18.849°E	1♀
*A.rutilipennis*	Cyprus: Limassol district, Zakaki marshes 34.644°N, 30.001°E	1♂, 1♀
*A.niger*	Sweden: Södermanland, Hölö, Tullgarn, Näsudden 58.95°N, 17.62°E	3♂♂, 3♀♀
	England: Hampshire, New Forest, Brockenhurst district, Balmer Lawn 50.830°N, 1.57°W	2♂♂, 3♀♀
	White Moor 50.821°N, 1.607°W	5♂♂
	Long Slade Bottom 50.800°N, 1.620°W	2♂♂, 3♀♀
*A.bameuli*	France, Corsica: Haute-Corse, by Lac de Melo 42.212°N, 9.025°E	6♂♂, 12♀♀
*A.bellumgerens*	Italy, Sicily: Provincia di Palermo, Parco delle Madonie, Piano Battaglia 37.880°N, 14.073°E	5♂♂, 1♀
	Provincia di Messina, Parco dei Nebrodi Monte Soro 37.529°N, 14.693°E	12♂♂, 5♀♀
*A.felix*	Italy: Provincia di L’Aquila, Campo Felice 42.215°N, 13.445°E	2♂♂, 3♀♀
*A.krelli*	Italy, Sardinia: Provincia di Nuoro, Badde Salighes 40.343°N, 8.902°E	3♂♂, 3♀♀
*A.muscorum*	Hungary:Hajdú-Bihar, Hortobágyi National Park, Kis-Kecskés area 47.677°N, 21.061°E	10♂♂, 10♀♀
	Jasz-Nagykun-Szolnok, Kisújszállás 47.216°N, 20.713°E	2♀♀
*A.wilsonae*	Spain: Provincia de Burgos, near Balneario de Corconte 43.009°N, 3.859°W	5♂♂, 6♀♀
	Provincia de Cantabria, Areños 43.112°N, 4.729°W	2♂♂
	Provincia de Madrid: Manzanares el Real 40.725°N, 3.860°W	2♂♂, 2♀♀
	Near El Vellón 40.76°N, 3.620°W	2♂♂, 2♀♀
	Provincia de Guadalajara, Matarrubia 40.852°N, 3.295°W	1♀

**Table 2.** Species sequenced in the present study with their locality data. Those belonging to subgenus Liothorax are considered as “ingroup” (IG) and the rest used to root the phylogeny as “outgroup” (OG). Last two columns show the GenBank submission codes for the successfully amplified sequences.

CODE	IG/OG	Subgenus	Species	Locality Data	COX1	CytB
AALioBam21	IG	*Liothorax*	*bameuli*	FRANCE, Corsica, Lac de Melo, above Corte, 19–25.vi.2011 short turf. Leg. R.B & E.M Angus	PP788597	PP791934
AApLioBam66	IG	*Liothorax*	*bameuli*	FRANCE, Corsica, Lac de Melo, above Corte, 19–25.vi.2011 short turf. Leg. R.B & E.M Angus	PP788594	PP791932
AApLioBam77	IG	*Liothorax*	*bameuli*	FRANCE, Corsica, Lac de Melo, above Corte, 19–25.vi.2011 short turf. Leg. R.B & E.M Angus	PP788595	PP791933
AApLioBam573	IG	*Liothorax*	*bameuli*	FRANCE Corsica Plateau d´Alzo 1500 m, 42°16'N, 9°04'E 21V1994 leg C. Zorn	PP788593	
AApLioBel125	IG	*Liothorax*	*bellumgerens*	ITALY, Sicily, Parco delle Madonie, Piano Battaglia, At deges of pool 1 MAY2013 Leg. R.B. & E.M. Angus	PP788596	PP791935
AApLioBel343	IG	*Liothorax*	*bellumgerens*	ITALY, Sicily, Parco delle Madonie, Piano Battaglia, At deges of pool 1 MAY2013 Leg. R.B. & E.M. Angus	PP788598	PP791936
AApLioBel593	IG	*Liothorax*	*bellumgerens*	ITALY, Sicily, Monte Soro 25APRIL2018 chromosome 4 27APR2018	PP788599	PP791958
AALioBel594	IG	*Liothorax*	*bellumgerens*	ITALY, Sicily, Parco delle Madonie, Piano Battaglia, #2, edge of pool 1 MAY2013 Leg. R.B. & E.M. Angus	PP788600	PP791937
AApLioFx22	IG	*Liothorax*	*felix*	ITALY. Abruzzo. Campo Felice. Washed into pool. 1.vi.2009. R. B. Angus	PP788601	PP791938
AApLioRu91	IG	*Liothorax*	*rutilipennis*	CYPRUS, Limassol Distrc, Zakaki, Rain puddle in lorry park, at edge of reedbed; 3/3/2005. R.B.Angus	PP788621	PP791940
AApLioRu110	IG	*Liothorax*	*rutilipennis*	CYPRUS, Limassol Distrc, Zakaki, Rain puddle in lorry park, at edge of reedbed; 3/3/2005. R.B.Angus mounted spec	PP788620	PP791939
AApLioKz236	IG	*Liothorax*	*kraatzi*	RUSSIA, Astrakhan P, Dosang env. (46°54'N, 47°55'E) 1–5/v/2014 cattle dung. A. Frolov & L. Akhmetova	PP788605	PP791944
AApLiKz270	IG	*Liothorax*	*kraatzi*	RUSSIA, Astrakhan P, Dosang env. (46°54'N, 47°55'E) 1–5/v/2014 cattle dung. A. Frolov & L. Akhmetova	PP788606	PP791945
AApLiKz272	IG	*Liothorax*	*kraatzi*	SLOVAKIA, S., E of Chlaba, 11.vi. 2014, 47°49'24.01"N, 18°50'56.23"E, 107 m a.s.l. Sandy place S of railway, at light, Leg. D. Král	PP788607	
AApLiKz273	IG	*Liothorax*	*kraatzi*	SLOVAKIA S., E of Chlaba, 11.vi. 2014, 47°49'24.01"N, 18°50'56.23"E, 107 m a.s.l. Sandy place S of railway, at light, Leg. D. Král	PP788608	
AApLioKr83	IG	*Liothorax*	*krelli*	ITALY, Sardinia, Nuoro Prov., Badde Salighes, 1 April 2012 R.B. & E.M. Angus unsexed	PP788604	PP791941
AApLioKr165	IG	*Liothorax*	*krelli*	ITALY, Sardinia, Sassari, La Ciaccia, Valledoria, 17 Feb 2008, old sheep dung, R.B.Angus	PP788603	PP791943
AApLioKr155	IG	*Liothorax*	*krelli*	ITALY, Sardinia, Nuoro, Altopiano della Campeda, 40°21.245'N, 8°47.044'E 580 m 18 MAY 2005 leg. Starke	PP788602	PP791942
AApLioMu59	IG	*Liothorax*	*muscorum*	HUNGARY. Jász-Nagykun-Szolnok kisújszállás. Mud in drying ditch. 14.iv.2011. R. B. Angus.	PP788612	PP791946
AApLioMu60	IG	*Liothorax*	*muscorum*	HUNGARY. Jász-Nagykun-Szolnok Kisviszalas. Mud in drying ditch. 14.iv.2011. R. B. Angus.	PP788613	PP791947
AApLioMu68	IG	*Liothorax*	*muscorum*	HUNGARY. Jász-Nagykun-Szolnok kisújszállás. Mud in drying ditch. 14.iv.2011. R. B. Angus.	PP788614	PP791948
AApLioMu299	IG	*Liothorax*	*muscorum*	HUNGARY, Hortobagy, 3;7/5/2011, R.B. & E.M. Angus	PP788609	PP791949
AApLioMu417	IG	*Liothorax*	*muscorum*	HUNGARY, Hortobagy, 5;7/5/2011, R.B. & E.M. Angus	PP788610	
AALioMu559	IG	*Liothorax*	*muscorum*	CZECH REPUBLIC, Bohemia, Hradec Kralove -16.4.2011 “Na Plachte”, lgt P. Kylies	PP788611	PP791955
AApLioNgSE	IG	*Liothorax*	*niger*	SWEDEN: Södermanland, Hölö: Tullgarn, Näsudden, 58.953788°N, 17.616277°E,15.v.2011. Hans-Erik & Livia Wanntorp	PP788615	PP791950
AApLioNgUK1	IG	*Liothorax*	*niger*	U.K., Hants. New Forest Long Slade Bottom 30.v.2002 R.B.Angus	PP788616	PP791963
AApLioPl37	IG	*Liothorax*	*plagiatus*	U.K., Norfolk, Hunstunton, 27/9/2001, R.B.Angus	PP788619	PP791951
AApLioPl131	IG	*Liothorax*	*plagiatus*	CHINA, Qinghai, N. Qinghai Hu, Gangca. Roadside pool 37°18'N, 100°11'E, 3370 m. 5.vi.2013,R.B. Angus, F.L. Jia & Y. Zhang	PP788618	PP791964
AApLioPl1	IG	*Liothorax*	*plagiatus*	UK, England, Norfolk, Hunstunton, 27/9/2001, R.B.Angus	PP788617	
AApLioW86	IG	*Liothorax*	*wilsonae*	SPAIN, Burgos, Balneario de Corconte, 12/03//2011 Leg R. Angus	PP788626	PP791952
AApLioW1	IG	*Liothorax*	*wilsonae*	SPAIN, Provincia de Burgos, Balneario de Corconte, 40.031°N, 3.884°W, 26.iv.2001. leg. R.B. Angus. (BMNH).	PP788622	PP791959
AApLioW248	IG	*Liothorax*	*wilsonae*	SPAIN, Burgos, Balneario de Corconte, 12/03/2011 Leg R. Angus	PP788623	PP791965
AApLioW264	IG	*Liothorax*	*wilsonae*	SPAIN, Burgos, Balneario de Corconte, 26.iv.2001 R.B. Angus	PP788624	PP791953
AApLioW606	IG	*Liothorax*	*wilsonae*	SPAIN, Burgos, Valle de Valdebezana, 42.993512, -3.831779, Virtus R. Angus leg	PP788625	PP791954
AApNiaVarMD53	OG	*Nialus*	*varians*	SPAIN, Madrid, Alcalá de Henares, 15/4/2008, lg J.F.Maté	PP788627	PP791960
AApNialVarAz208	OG	*Nialus*	*varians*	AZERBAIJAN, 4.v.2014 fish pond btw Kalageyli & Xalilli 40°36'55.8"N, 48°12'37.5"E 220 m lg Faille, Fresneda, Ribera & Rudoy	PP788628	PP791957
AApSubSt1	OG	*Subrinus*	*sturmi*	SPAIN, Madrid, La Acebeda, 41.091714, -3.627400 01/08/1999 1375 m lg. J.F.Maté	PP788629	PP791962
AApLabPseu1	OG	*Labarrus*	*pseudolividus*	NAMIBIA, Mukwe Dist., W. Capriva Pk Divvju; 18°04'04"S, 21°28'51"E 31/xii/1998 campsite at U.V. D Mann Leg.	PP788630	PP791961

Morphological data were obtained from 209 specimens (183 ingroup and 26 outgroup taxa) from several collections and from field-collected specimens (Table 3). Only specimens that could be identified with certainty a priori were used with the exception of the *niger*-*muscorum* complex. For this latter group there were a number of specimens from Czechia that could not be unambiguously categorised and so they were identified as “*A.niger* cf.” as a way to compare them with the unambiguously identifiable *A.niger* and *A.muscorum* (Ádám, 1994) material. These *A.niger* cf. specimens were used only in the principal component analyses to explore their relation within the morphospace, but not for other analyses as they did not represent a proper category. Although we aimed to sample at least six specimens per taxon, a few species were represented by only a few historical specimens. Due to the limited availability of material for some species and the low sexual dimorphism in the group (Dellacasa and Dellacasa 2005) male and female specimens were used together. The linear measurements used here are not affected by sexual dimorphism.

**Table 3.** List of species and number of specimens used in the morphometric part of the study.

Subgenus	Species	# Specimens
*Liothorax*	*alberti*	12
*Liothorax*	*bameuli*	11
*Liothorax*	*bellumgerens*	9
*Liothorax*	*chellala*	7
*Liothorax*	*discoides*	8
*Liothorax*	*felix*	7
*Liothorax*	*isikdagensis*	4
*Liothorax*	*kraatzi*	13
*Liothorax*	*krelli*	7
*Liothorax*	*muscorum*	16
*Liothorax*	*niger*	20
*Liothorax*	*niger cf*	6
*Liothorax*	*plagiatus*	23
*Liothorax*	*rodrigoi*	3
*Liothorax*	*rusakovi*	6
*Liothorax*	*rutilipennis*	13
*Liothorax*	*sinoplagiatus*	4
*Liothorax*	*wilsonae*	14
*Labarrus*	*lividus*	6
*Nialus*	*varians*	9
*Subrinus*	*sturmi*	11
**Total specimens measured**		**209**

### ﻿Methods

Habitus photographs of whole beetles and parts of the external structures were taken with a Leica M125 stereomicroscope + Canon EOS 550D digital camera, and of uninflated aedeagi and mouthparts mounted on slides in dimethyl hydantoin formaldehyde (DMHF) resin with a Zeiss Axioskop + Canon EOS 450D digital camera, in the Sackler Bioimaging Laboratory of the Natural History Museum, London. Both were stacked using Helicon Focus software. Where aedeagi were partly inflated as a result of the hypotonic potassium chloride treatment for chromosome preparation the endophallus was further extruded by gentle squeezing with forceps. Detailed methods are given by Angus et al. (2000). The preparations were viewed uncoated for scanning electron microscopy in the Natural History Museum, London and the Biomedical Imaging Unit of Southampton General Hospital. Occasionally the endophallus of old, mounted specimens had been extruded as a result of partial decomposition. In these cases the aedeagi were transferred to a 5% solution of potassium hydroxide to soften them, and the aedeagus was squeezed very gently to further inflate the extruded endophallus. Inflated aedeagi were transferred to alcohol for critical-point drying. They were then photographed as for other external structures, and, in some cases scanning electron micrographs (SEMs) of uncoated aedeagi were taken. They were viewed in low vacuum mode using secondary electrons on a FEI Quanta 200 SEM. This work was carried at the Biomedical Imaging Unit in Southampton. Endophallus teeth were normally measured from SEM images, usually at 1200× magnification but sometimes at 600 or 300×. The teeth of *A.isikdagensis* Balthasar, 1953 were measured from a photograph.

Pronotal bases were also viewed as SEM images, to show the varying development of the border. In this case the whole beetle was mounted and the pronotal bases were viewed uncoated in low vacuum mode using secondary electrons on a FEI Quanta 250 SEM. This work was also carried at the Biomedical Imaging Unit in Southampton General Hospital.

SEMs were also used to check the nature of the surface reticulation of elytra and metasterna. For this, back-scattered electrons were used for imaging, as these categorically show only the surface sculpture. Images were taken in the Electron Microscope Unit of the Natural History Museum, London, using a Zeiss Leo 1455VP SEM in low vacuum mode on uncoated specimens.

Chromosome preparations were made from dividing cells in testes and midguts of adult beetles, using the methods described by Shaarawi and Angus (1991) and Angus (1982). In brief, living beetles are placed in watch glasses of 0.1% colchicine solution in insect saline buffered to pH6.8 with Sörensen’s phosphate buffer, and the abdomen is partially detached. After ca 12 min the beetle is transferred to a watch glass of ½-isotonic (0.48% KCl) at pH 6.8. The abdomen is fully detached and the mid gut and testes are dissected out. After 12 min the midgut and testes are transferred to a watch glass of fixative (3:1 absolute ethanol: glacial acetic acid). The fixative is changed 3× and the tissue left for at least 1 h. At this stage, the beetle is killed with boiling water and either mounted on a card or placed in absolute ethanol for DNA analysis. Small pieces of tissue are then transferred to clean microscope slides, excess fixative is removed and a small drop of 45% acetic acid is dropped on to the tissue, to dissociate the cells. The tissue is pulled apart in this drop with fine pins. A small drop of fixative causes the cell suspension to spread over the slide where it is allowed to dry. It is then stained with 1% Giemsa stain at pH 6.8. The dry slide is then examined under a microscope and suitable chromosome spreads are photographed under oil immersion. After this the oil is removed using two changes of xylene and one of absolute ethanol, in Coplin jars, and the slide is once more allowed to dry. At this stage C-banding is attempted, ideally on two-day-old slides. The slides are immersed in a saturated solution of barium hydroxide at room temperature (ca 23 °C), initially for 3 min. They are then rinsed in three changes of distilled water at pH 6.8 and placed in a Coplin jar of 2× SSC (0.3 M NaCl + 0.03 M trisodium citrate) at 55–60 °C and left for 1 h. They are then rinsed in 3 changes of distilled water at pH 6.8 and stained for 10 min in 1% Giemsa, as before. If C-banding has not developed the C-banding procedure can be repeated (any immersion oil being removed as before) and the slide the slide can be restained. This cycle can be repeated as often as is found necessary – each repeat usually involves an additional 1 min in barium hydroxide. Meiosis metaphase I chromosomes usually require more treatment than mitotic ones.

Preparations were photographed onto high-contrast microfilm and printed at a magnification of 3000×. They were than scanned into a computer and the chromosomes arranged using Adobe Photoshop. Chromosomes were measured once arranged as karyotypes, and Relative Chromosome Lengths (RCL, the length of each chromosome expressed as a percentage of the total haploid autosome length in the nucleus) and Centromere Indices (CI, the length of the short arm of a chromosome expressed as a percentage of the total length of the chromosome) were calculated. These terms and calculations are as recommended by the Paris Conference (1971). In practice CI measurements are subject to considerable variation and are best expressed as a limited number of categories. Based on Sumner (2003) these are metacentric, CI 50–46; submetacentric, CI 45–26; subacrocentric, CI 25–5, and acrocentric (including telocentric), CI < 15. RCL data are given in Table 4 and a summary of chromosome characters, including CI, is given in Table 5.

**Table 4.** Relative Chromosome Lengths, mean, 95% confidence limits, number measured. Important distinctive values shown in cells with yellow background.

	1	2	3	4	5	6	7	8	9	X	Y
*A.niger* S + NF	17.68 17.09–18.27 N=12	16.23 15.53 -16.92 N=12	12.71 11.83–13.59 N=12	12.32 11.72–12.92 N=12	11.40 10.88–12.12 N=11	9.49 8.82–10.17 N=12	8.03 7.76–8.30 N=12	7.37 6.94–7.81 N=12	6.32 5.84–6.81 N=11	15.55 13.46–17.64 N=7	13.88 12.03–15.72 N=5
*A.muscorum* Hortobágyi	14.5 14.2–14.83 N= 59	16.78 16.46–17.09 N=60	12.92 12.67–13.17 N=60	12.71 12.41–13.02 N=59	10.82 10.55–11.11 N=60	9.65 9.35–9.95 N=60	8.83 8.59–9.06 N=60	7.46 7.26–7.67 N=60	6.29 6.09–6.5 N=60	18.33 17.63–19.03 N=44	5.95 5.15–6.75 N=16
*A.muscorum* Kisújszállás ♀♀	13.41 12.13–14.69 N=4	17.19 16.31–18.07 N=4	11.63 10.36–12.91 N=4	12.75 11.88–13.62 N=4	11.00 10.40–11.60 N=4	10.31 9.56–11. 05 N=4	9.00 8.09–9.91 N =4	7.19 5.94–8.45 N=4	6.74 6.24–7.24 N=4	18.92 17.09–20.76 N=4	
*A.felix*	13.39 12.93–13.86 N=14	13.48 13.12–13.84 N=14	12.84 12.22–13.45 N=14	12.21 11.86–12.56 N=14	11.57 11.19–11.95 N=14	10.61 10.2–11.02 N=14	9.54 9.23–9.85 N=14	9.29 8.88–9.71 N=14	7.66 7.16–8.16 N=14	10.76 9.97–11.55 N=8	2.42 2.0–2.84 N=6
*A.bellumgerens*	16.95 15.95–17.94 N=12	15.43 14.78–16.08 N=12	11.96 11. 40– 12.52 N=12	10.59 9.93–11.25 N=12	10.19 9.62–10.75 N=12	9.93 9.59–10.27 N=6	9.17 8.64–9.69 N=12	8.80 8.22–9.38 N=12	7.40 6.83–7.97 N=12	19.64 18.09–21.18 N=9	8.71 5.17–12.25 N=3
*A.bameuli*	13.16 12.45–13.87 N=12	12.82 12.24–13.42 N=12	13.19 12.49–13.89 N=12	11.97 11.48–12.46 N=12	11.31 10.95–11.67 N=12	11.14 10.24–12.04 N=11	9.59 8.96–10.22 N=12	9.3 9.01–9.59 N=12	8.96 8.5–9.41 N=11	13.74 12.2–15.28 N=7	3.61 1.89–5.34 N=5
*A.krelli*	13.57 12.9–14.25 N=16	12.88 12.35–13.42 N=16	12.76 12.11– 13.42 N=16	11.89 11.4–12.39 N=16	11.49 11.15–11.82 N=16	10.91 10.61 - 11.21 N=16	10.09 9.18–11 N=11 5.22 4.63–5.8 N=5 Short form	9.68 9.14–10.21 N=16	9.5 9.07–9.92 N=16	15.09 14.08–16.1 N=12	3.61 2.98–4.24 N=4
*A.wilsonae*	14.74 14.11–15.37 N=16	12.16 11.59– 12.73 N=16	12.97 12.51–13.44 N=16	11.63 11.29–11.98 N=16	10.89 10.6–11.19 N=16	10.07 9.68–10.49 N=16	9.81 9.52–10.11 N=16	9.22 9.02–9.41 N=16	8.26 7.96–8.56 N=16	11.53 10.71–12.35 N=10	3.5 3.14–3.84 N=6

**Table 5.** Summary of the major features of the karyotypes.

Chromosome	*A.plagiatus*	*A.kraatzi*	*A.rutilipennis*	*A.niger*	*A.muscorum*	*A.felix*	*A.bellumgerens*	*A.bameuli*	*A.krelli*	*A.wilsonae*
1	m	m. hs	sm	m.hl	sm.hl(part)	m-sm hl weak	m.hl	m.hl	m.hl	m.hl
2	m	m.hs	m	m	sm	m	m	m	m	sm
3	m	sm.hs	sm.hl	m.	sa.hs	m.hs	m	sm.hl	sm.hl	sm.hl
4	m.**2c**?	sm.hs	sm	sm	sm	m	m	sm.hl	sm.hl	sm.hl
5	sm/sa	sm.hs	sm.hl	sm	sa.hs	m	m	sm.hl	sm.hl	sm.hl
6	m	sm.hs	m	sm.hs	sa.hs	m.hl	sm	sm	sm.hl	sm.hl
7	sm **2c**?	sm.hs	m.hl	sm	sm	m	m	sm	sm.hl/sa	sm.hl
8	sm **2c**?	sa	sm.hl	sm	sa	m/sa	sm	m.hl	m.hl	sm.hl
9	sm/sa	sa	m	sa	sm-sa	sm	sm	m/sa	m/sa	sm
X	sm/sa	[10] sa	sm.hl	m.hl	m-sm.hl	sm hl	m.hl	sm.hl	sm.hl	sm.hl
y	sm(dot)	-	m(dot)	m	m(dot)	m(dot)	m (small, h)	sa (small)	m(dot)	m(dot)
B		1.h		1–3.sa.h						
Distinctive features	No hl or hs	8 hs	5 hl	y long m	C1 shorter than C2	C1 hl weak	2 hl, including C1	6 hl not C6,7	8 hl C7 &9 polymorphic	8 hl No polymorphisms

C-chromosome; m-metacentric; sm-submetacentric; sa-subacrocentric; hl-heterochromatic long arm; hs-heterochromatic short arm; h-heterochromatic; /- either borderline or alternative (polymorphic). **2c**?-possible secondary constriction.

For molecular analysis all collected specimens were taken back alive to the lab by RBA for karyological work. After dissection of the abdomen, the specimens were stored in 95% ethanol until DNA extraction. Isolation of DNA from specimens followed a modified protocol of the “salting out” method of Miller et al. (1988). Briefly, whole specimens were air dried in 1.5 ml microcentrifuge tubes, after which a mix of proteinase K (5 μl, 10 mg/ml) in lysis buffer (300 μl TNES buffer, pH 7.5, 10% SDS) was added and the samples incubated for 6–8 h at 37–40 °C to ensure complete tissue digestion. Tubes were shaken every hour to ensure that the lysis solution could access the whole specimen. Afterwards the specimens were chilled to 4 °C for 1 h before the next step. Once chilled, 85 μl of saturated NaCl solution (> 5M) was added to each vial for a total volume of 390 μl. The tubes were shaken for 15 sec to ensure mixing and spun for 5 min at 14k rpm. The supernatant was removed to a new set of 1.5-ml vials containing 400 μl of 100% EtOH. They were shaken for 15 sec and spun at 14k rpm for five minutes. The supernatant was discarded and the DNA pellet washed with 500 μl of ice cold 70% EtOH. After discarding the supernatant the pellets were allowed to air dry before resuspending in 20–50 μl of TE buffer (pH 8.0) and storage at -20 °C.

Sequencing. PCR reactions were carried out using PCR beads (GE27-9557-01, Illustra™ PuReTaq RTG PCR, 0.2 ML, Cytiva, Buckinghamshire, U.K.) to which 0.2–0.5 μl of genomic DNA suspension was added to an overall reaction volume of 25 μl. Typical conditions were as follows: 1 cycle of denaturation at 94 °C for 2 min as an initial step followed by 35–40 amplification cycles at 94 °C for 30 sec for denaturing, 47–49 °C for 30 sec, 68–72 °C for 30 sec for extension, and a final extension step at 72 °C for 5 min. PCR products were cleaned via sodium acetate/ethanol precipitation. Once dried, products were sent to an outside sequencing lab (SECUGEN S.L., Madrid, Spain) for final Sanger sequencing.

Two mtDNA regions were sequenced, the 39 end of cox1 (Pat [5’TCCAATGCACTAATCTGCCATATTA] and Jerry [5’CAACATTTATTTTGATTTTTTGG]), and cytochrome b (cb-1 [5’ TATGTACTACCATGAGGACAAATATC] and cb-2 [5’ATTTACACCTCCTAATTTATTAGGAAT]) (Simon et al. 1994). DNA chromatographs were visually checked in Chromas (v. 2.6.6.) for ambiguous or erroneous base readings. Final sequences were exported into AliView (v. 1.28; Larsson 2014) for editing and alignment of sequences. Aligned datasets were exported as fasta files into MEGA11 (MEGA11: Molecular Evolutionary Genetics Analysis v. 11; Koichiro et al. 2021) for concatenation and analysis.

### ﻿Phylogenetic analysis

Parsimony analysis was run in MEGA 11, which was also used to run the 1000 bootstrap replications (50% character removal). The parsimony search employed the Tree-Bisection-Regrafting (TBR) algorithm (Nei and Kumar 2000) with search level 3 in which the initial trees were obtained by the random addition of sequences (100 replicates). All positions with less than 95% site coverage were eliminated (i.e., fewer than 5% alignment gaps), missing data and ambiguous bases were allowed at any position (partial deletion option). Bootstrapping was run concurrently with the parsimony tree search using the default conditions in Mega11. Jackknife support was estimated in PAUP (v. 4.0a169: Swofford 2003) using default conditions. For decay indices, one of the trees of the parsimony search was used to generate a search file in TreeRot (v. 3.0; Sorenson and Franzosa 2007). The settings for the decay index file were Branch-and-bound search, gaps treated as missing, with addition sequence set to furthest “Maxtrees” set at 500 (no automatic increase), “Multrees” on and branch collapse on and trees unrooted.

For the Maximum Likelihood method, the model implemented was the General Time Reversible model. A discrete Gamma distribution was used to model evolutionary rate differences among sites (5 categories (+G, parameter = 1.2620)). The rate variation model allowed for some sites to be evolutionarily invariable ([+I], 62.14% sites). All positions with less than 95% site coverage were eliminated, i.e., fewer than 5% alignment gaps, missing data, and ambiguous bases were allowed at any position (partial deletion option).

Genetic distances were calculated to quantify sequence divergences within species and between species using p-distance and using Kimura’s (Kimura 1980) two-parameter (K2P) models as implemented in MEGA 11. The p-distance is the most commonly reported genetic distance method whereas K2P distance is preferred if genetic divergence is low (Hebert et al. 2003). Interspecific distances were calculated for those species with at least two sequences; hence no intraspecific divergence could be reported for A. (L.) felix sp. nov. (intraspecific cell labelled n/c).

**Figure 1.**
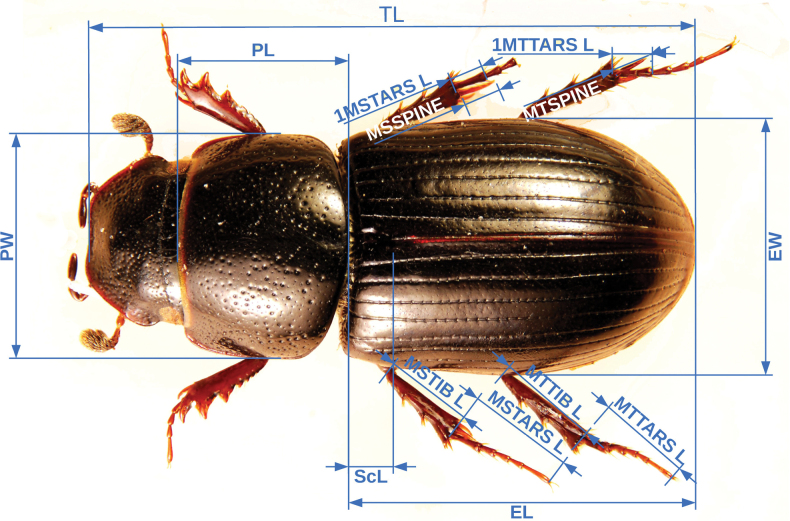
Linear morphological measurements collected in this study. For definitions refer to Table 6.

For the multivariate analysis fifteen linear measurements were collected for each specimen pertaining to the body and legs (Table 6). These measurements were selected based on the discriminatory potential as evidenced by previous work on *Aphodius* taxa (Maté 2003) as well as on the observations made on the tarsal characters of *A.wilsonae* (Maté & Angus, 2005). The measurements (Fig. 1) were made on stacked images of the specimens as well as mounted material. For the material that was imaged, specimens were mounted and at least three perspectives were captured per specimen (dorsal, lateral, and ventral) to ensure that the anatomical features of interest were properly oriented. Images were taken wither with a Nikon D40 camera mounted on a Wemacro rig and aligned using Zerene Stacker (Zerene Systems LLC, WA, USA), or when photographed in the Sackler imaging laboratory of the Natural History Museum, London, a Leica MZ 125 stereomicroscope equipped with a Cannon DSLR camera, and the images stacked using Helicon Focus 7. All images were size-calibrated using a reference measure, and the features of interest were measured either in ImageJ (Schneider et al. 2012) or in Photoshop (Photoshop CS5.1). Each measurement was taken twice to the nearest hundredth of a millimetre and averaged for each feature/specimen. A random subsample of specimens was remeasured to check for repeatability. Measurements were taken, when possible, from the same side to minimise the effects of asymmetry. All measurements were curated for outliers that could indicate input or measurement errors.

**Table 6.** Tabulated list of the fifteen morphological linear measurements collected in the present study with their acronyms.

Code	Description
**EL**	Elytral length, from base of elytra at corner of scutellum to apex measured dorsally.
**PL**	Pronotal length, measured dorsally.
**PW**	Pronotal width, the widest distance across the pronotum as measured dorsally.
**EW**	Elytral width, the widest distance across combined elytra as measured dorsally.
**MTTIB L**	Metatibial length, distance from joint to apex of tibia.
**DV HEIGHT**	Dorsoventral height, highest point from dorsum to ventrum as measured laterally (not illustrated).
**ScL**	Scutellar length, medial length from apex to inflexion point at base.
**MT TARS L**	Metatarsal length, combined length of all metatarsi.
**1MT TARS L**	Length of first metatarsal segment as measured between joints.
**MTSPINE**	Metattibial spine length, distance from joint to apex.
**MSTIB L**	Mesotibial length, distance from joint to apex of tibia.
**MSTARS L**	Mesotarsal length, combined length of all five mesotarsi.
**1MSTARS L**	Length of first mesotarsal segment as measured between joints.
**MSSPINE**	Mesotibial spinal length
**TL**	Total length

### ﻿Statistical analyses

Prior to all analyses, measurements were size corrected using total body length (TBL) as the size correction. Although most previous studies have used a ratio of each linear measure against TBL, aphodiine scarabs show allometric body size variation (Maté 2003) which cannot be completely removed by performing a ratio, making it difficult to accurately discriminate taxa using morphometric techniques (Sidlauskas et al. 2011). To correct for allometric size effects, the GroupStruct (v. 0.1.0) R-package was used (Chan and Grissmer 2022). After extracting size, the measurements were cross checked against total length to ensure that the size correction procedure was effective and the new data matrix was used for all further analyses. All statistical analyses were performed in the RStudio (v. 1.2.5033; RStudio Team 2020) platform using the packages tidyverse (v. 1.3.0; Wickham et al. 2019), tibble (v. 3.1.6; Müller and Wickham 2023), dplyr (v. 1.0.7; Wickham et al. 2021) and yarr (v. 0.1.5; Phillips 2017) as well as others that are individually mentioned below.

### ﻿Principal component analyses

We derived the orthogonal morphological axes using principal component analysis (PCA) on a matrix of the allometrically corrected linear measurements, computed using the packages Psych (v. 1.9.12.31; Revelle 2021) and nFactors (v. 2.4.1). Prior to analysis the data was tested for suitability via several indicators (Bartlett’s test for sphericity, Bartlett Test of Homogeneity of Variances, and KMO [Kaiser, Meyer and Olkin Measure of Sampling Adequacy]). The number of components to extract was estimated examination of a scree plot, parallel analysis and VSS [Very Simple Structure]. Different rotation methods were performed to better load each variable to the components. The resulting factors were used to generate the bivariate plots.

### ﻿Morphological divergence

Divergence between groups and within groups was assessed by measuring the morphological divergence across all the measurements as well as individual morphological parameters. Euclidian and Manhattan distances were computed and compared, with both methods giving the same results, hence subsequent analyses used only Euclidian distances. Significance between groups was tested via the pairwise Wilcoxon Test and Tukey’s Test with the *p*-values adjusted using the Bonferroni method.

## ﻿Results

### ﻿Taxonomy

Treatment of *Liothorax* as a genus or as a subgenus of *Aphodius*

Motschulsky (1860) erected the genus *Liothorax* for three species, including *Scarabaeusplagiatus* Linnaeus, 1767, the only one of the trio still included in *Liothorax*, and which G. Dellacasa (1983) designated as the type species. Following Bedel (1911) most authors, including Baraud (1992) have regarded *Liothorax* as a subgenus of *Aphodius* Hellwig, 1798. However, Dellacasa et al. (2001a) proposed the elevation of all the subgenera of *Aphodius* to generic level, and this arrangement is used in Volume 3 of the latest Catalogue of Palaearctic Coleoptera (Dellacasa et al. 2016). Nevertheless, in the absence of phylogenetic research demonstrating the need to change the rank of *Liothorax* we follow majority of authors and treat it as a subgenus of *Aphodius* in this revision, as recommended by two referees and with the support of the editor.

**Subgeneric characters**

**Figure 2.**
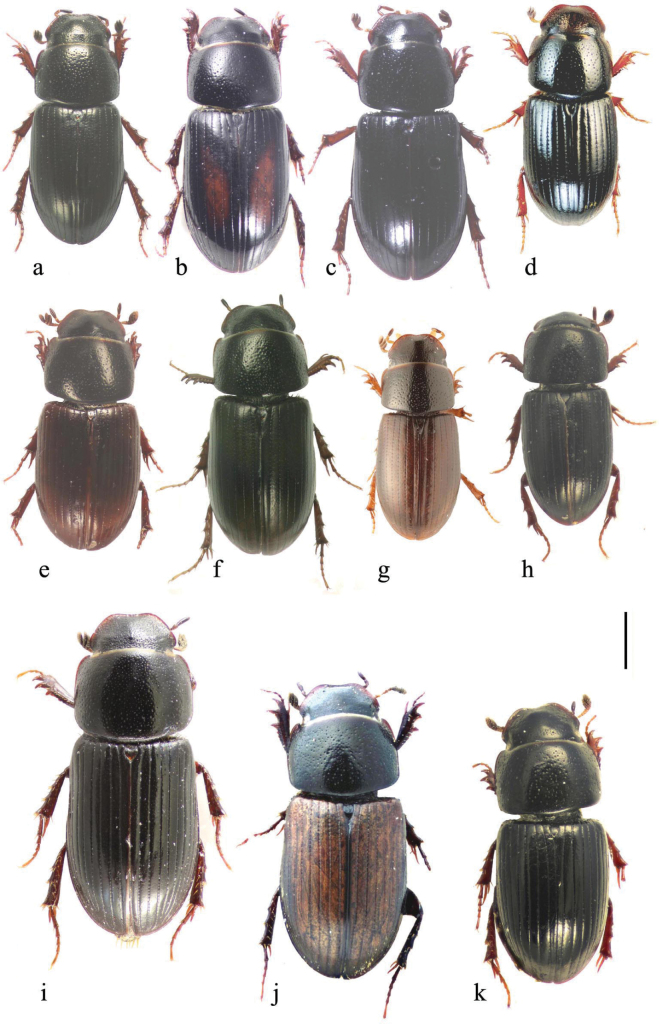
**a–k** whole beetles **a***A.plagiatus*, CZ, Moravia **b***A.plagiatus*, MG, Mongolia **c***A.jakutorum*, holotype, RU, East Siberia **d***L.rodrigoi* sp. nov., holotype SP, Aranjuez **e***A.rutilipennis* (*A.ressli*, holotype) TR, Hatay, Iskenderun **f***A.rutilipennis*, large ♀, CY, Akrotiri **g***A.rutilipennis* (*A.cypricola*, holotype) CY **h***A.chellala* sp. nov., holotype, AG, Chellala **i***A.rusakovi*, ♂ paratype, RU, Orenburg **j***A.discoides*, neotype (*A.bytinskisalzi*, holotype) IS, Kuneitra **k***A.discoides*, black form from TR, Karakurt. Scale bar: 1 mm.

**Figure 3.**
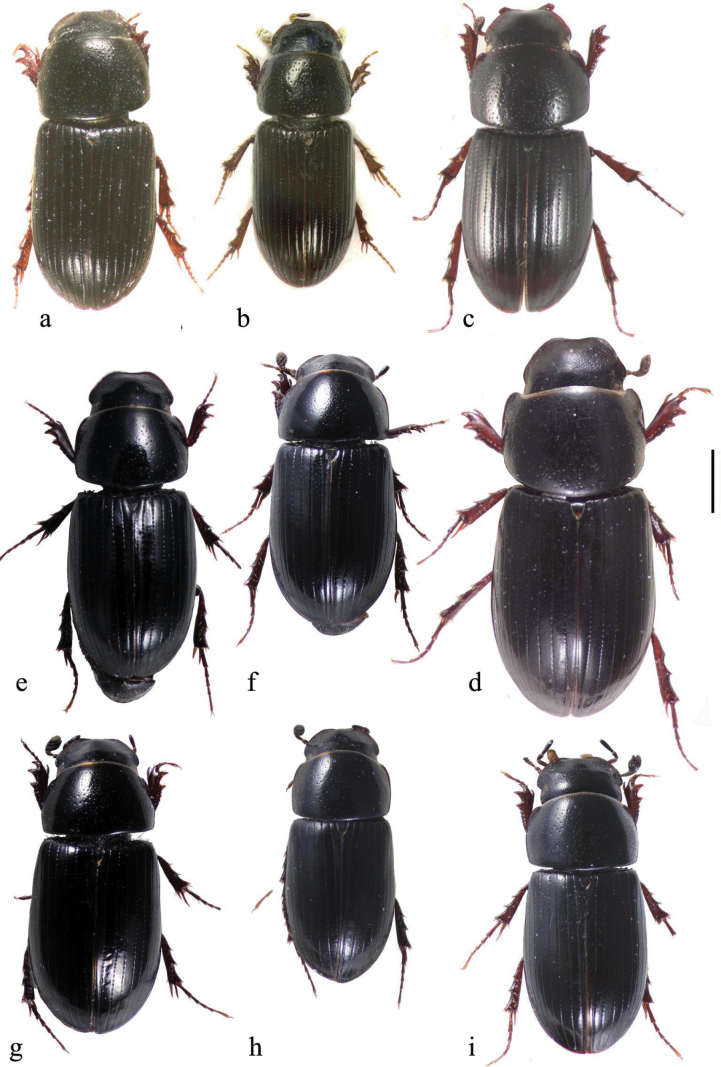
**a–i** whole beetles **a**, **b***A.kraatzi***a** AF, Kabul, ♀ **b** KZ, Kyzil Kum **c**, **d***A.isikdagensis*, TR, Ḉamildere **c** ♂ paratype **d** ♀ paratype **e**–**g***A.alberti* sp. nov., TR. Rize, Ovitdagi **e** holotype ♂ **f** paratype ♂ **g** paratype ♀ **h***A.alberti*?, ♂, AR, Armenia **i***A.isikdagensis*?, ♂, TR, Artvin. Scale bar: 1 mm.

**Figure 4.**
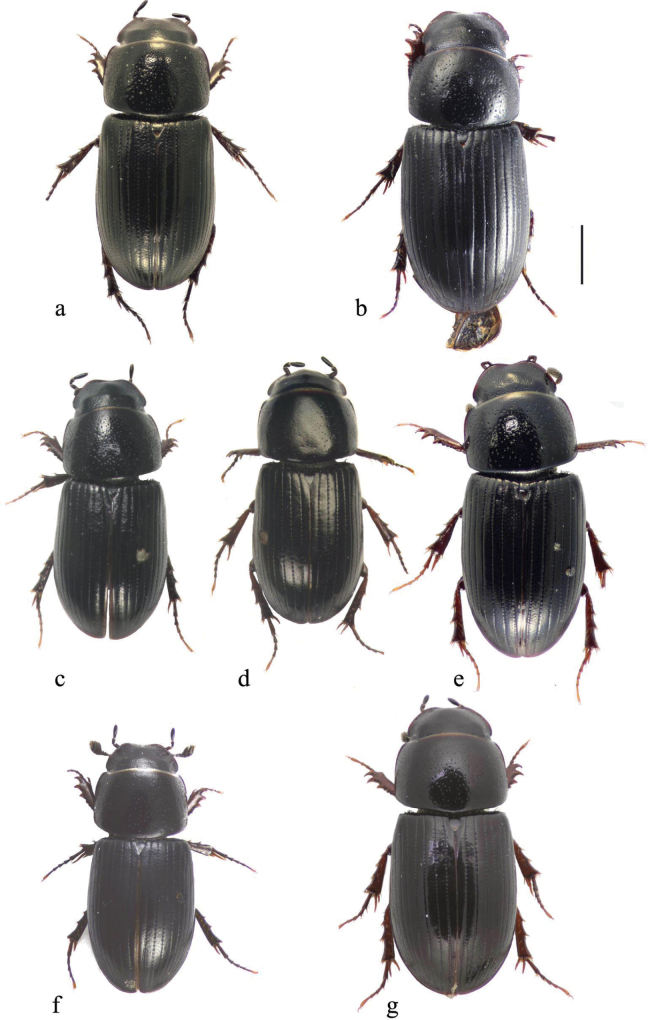
**a–g** whole beetles **a***A.niger*, SV, Tullgarn **b***A.muscorum*, holotype HU, Vörös tó **c***A.felix* sp. nov., holotype, IT, Campo Felice **d***A.bameuli* sp. nov., holotype, Corsica, by Lac de Melo **e***A.krelli* sp. nov., holotype, IT, Sardinia **f***A.bellumgerens* sp. nov., holotype, IT, Sicily, Piano Battaglia **g***A.wilsonae*, holotype, SP, Provincia de Burgos, Balneario de Corconte. Scale bar: 1 mm.

**Figure 5.**
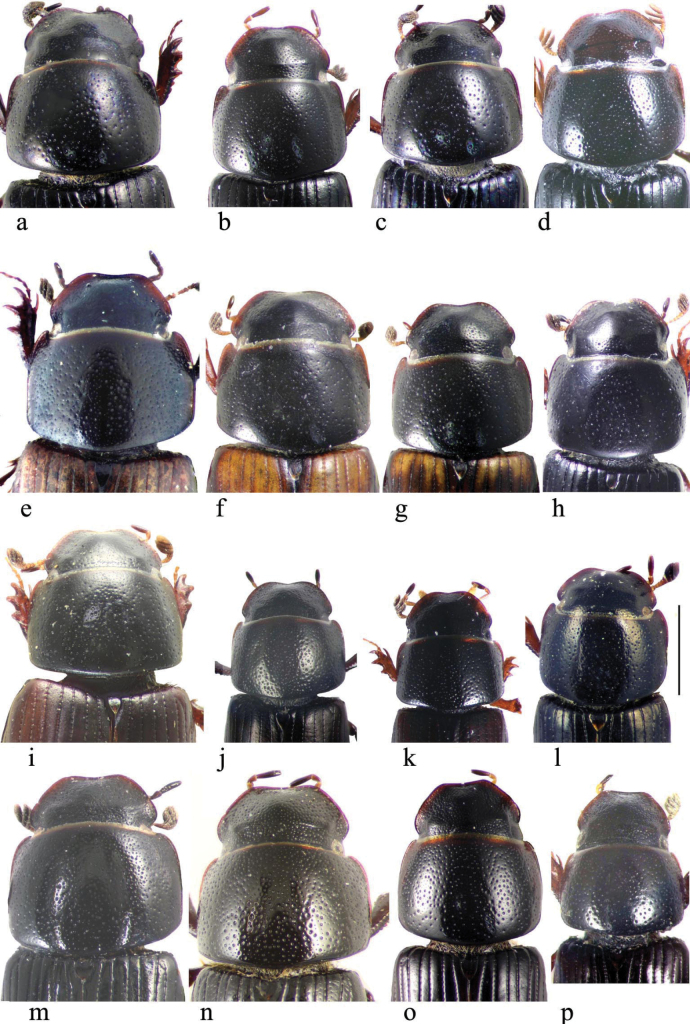
**a–p***A.plagiatus* group, heads and pronota **a–c***A.plagiatusplagiatus***a** GB, Norfolk **b** CZ, Moravia, Dobre Pole **c** RU, St Petersburg **d***A.p.sinoplagiatus* ssp. nov., paratype, CH, Qinghai, Gangca **e–g***A.discoides***e** neotype IS Kuneitra **f** TR, Kyzil Dag **g** Turkey Muş **h***A.discoides*, black ♂, TR, Karakurt **i–k***A.rutilipennis***i***A.ressli*, holotype, TR, Hatay **j** CY, Akrotiri **k***A.cypricola*, holotype, CY **l***A.chellala* sp. nov., holotype, AG, Chellala **m***A.rusakovi*, paratype ♂, RU, Orenburg **n***A.ballerioi* sp. nov., paratype ♂, TR, Rize **o**, **p***L.kraatzi***o** RU, Astrakhan **p** SR. Scale bar: 1 mm.

**Figure 6.**
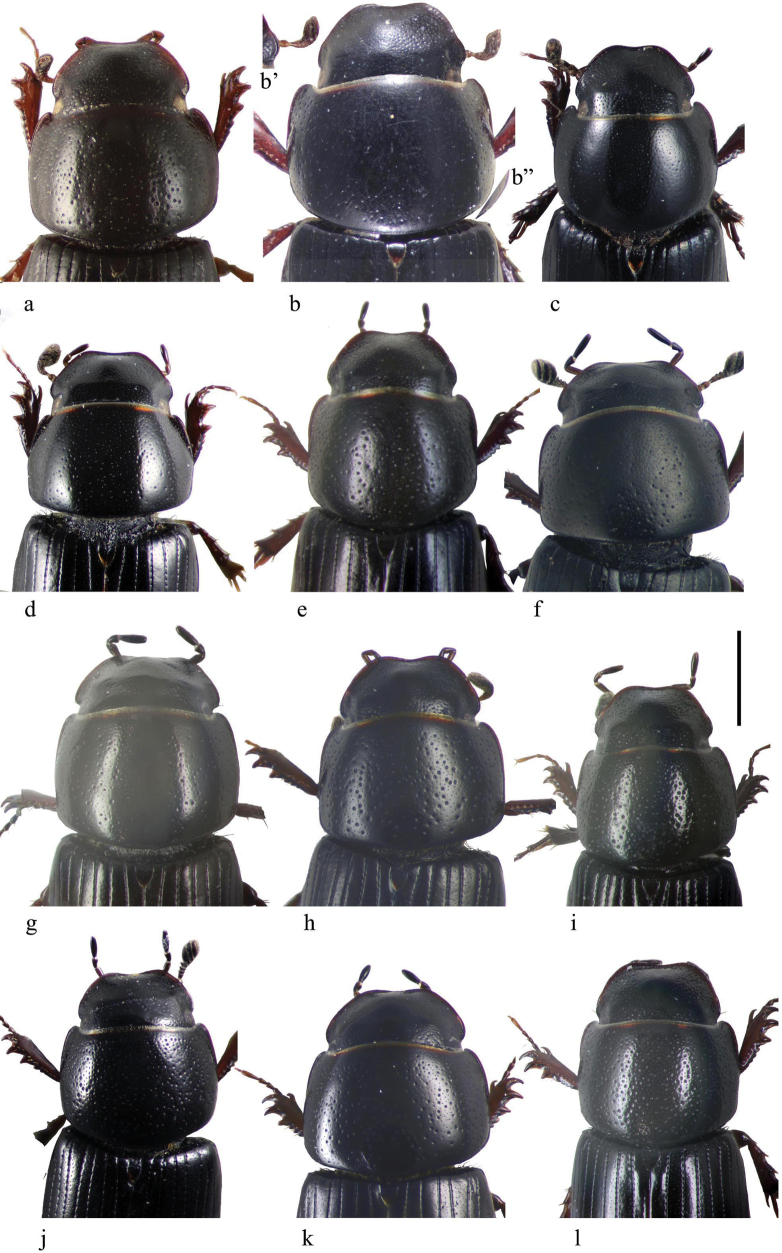
**a**–**l***A.niger* group, heads and pronota **a**, **b***A.isikdagensis*, paratypes, TR Ḉamildere **a** ♂ **b** ♀ **b**’ detail of right lateral part of frons and gena, different orientation **b**” detail of right posterior of pronotum, different orientation **c**, **d***A.alberti* sp. nov., TR, Rize, Ovitdagi **c** holotype **d** ♀paratype **e***A.niger*, GB, Hampshire, New Forest **f***A.muscorum*, HU, Hortobágyi **g***A.bameuli* sp. nov., holotype, FR, Corsica, by Lac de Melo **h***A.krelli* sp. nov., holotype, IT, Sardinia **i***A.felix* sp. nov., holotype, IT, Campo Felice **j***A.bellumgerens* sp. nov., holotype, IT, Sicily, Piano Battaglia **k**, **l***A.wilsonae*, SP **k** holotype, Provincia de Burgos, Balneario de Corconte **l** paratype, Provincia de Madrid, Manzanares el Real. Scale bar: 1 mm.

**Figure 7.**
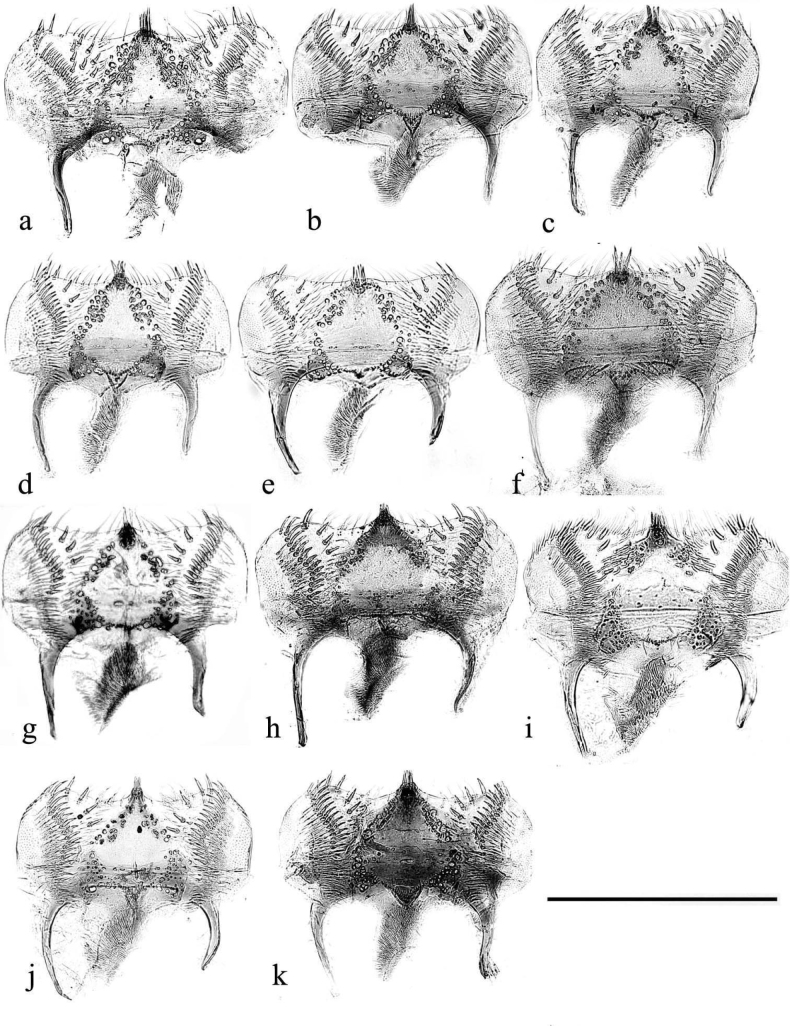
**a–k** epipharynxes **a– f***A.plagiatusplagiatus***a**, **b** GB, Hunstanton, Norfolk **c** CZ, Moravia, Dobré Pole **d** RU, Pavlovsk, St Petersburg **e***A.p.sinoplagiatus* ssp. nov., paratype, CH, Gangca, Qinghai **f***A.p.sinoplagiatus*?, CH, “Pekin, coll. Fry” **g***A.rodrigoi* sp. nov., holotype, SP **h***A.discoides*, TR **i***A.rusakovi*, paratype, RU, Orenburg **j***A.rutilipennis*, CY, Akrotiri **k***A.chellala*, sp. nov., paratype, AG, Chellala. Scale bar: 0.5 mm.

**Figure 8.**
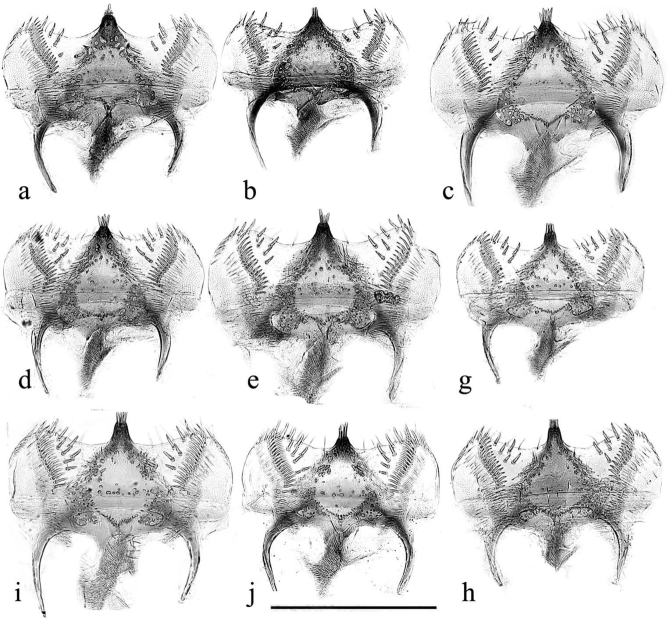
**a–k** epipharynxes **a**, **b***A.kraatzi*, SV **c**, **d***A.isikdagensis* paratypes, TR, Ḉamildere **e**, **f***A.felix* sp. nov., paratypes **g**, **h***A.bameuli* paratypes **i***A.alberti*?, AR **j***A.krelli* sp. nov., paratype **k**, **l***A.alberti* sp. nov., paratypes. Scale bar: 0.5 mm

**Figure 9.**
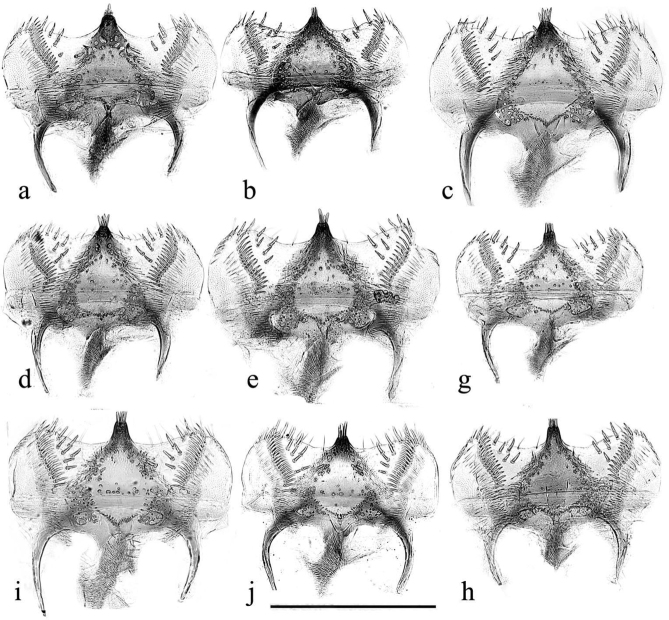
**a–i** epipharynxes **a**–**c***A.niger***a** SV, Tullgarn **b**, **c** GB, Hampshire, New Forest **d***A.niger*?, CZ, Hradec Králové **e***A.muscorum*, HU, Hortobagyi **f**, **g***A.bellumgerens* sp. nov., IT Piano Battaglia, paratypes **h**, **i***A.wilsonae* paratypes, SP **h** El Vellón **i** Manzanares el Real. Scale bar: 0.5 mm.

In this illustrated account of the genus the two-letter abbreviations of countries from where material was obtained are those used in the Catalogue of Palaearctic Coleoptera. *Liothorax* species are elongate parallel-sided beetles, to some extent hemicylindrical in shape (Figs 2–4) though occasionally less elongate and more rounded (*A.rodrigoi* sp. nov., Fig. 2d; *A.krelli* sp. nov. and *A.wilsonae*, Fig. 4e, g). The head nearly always lacks any trace of tubercles on the frontoclypeal suture, although there can be a very slight medial one in some male specimens of *A.rusakovi* Gusakov, 2004, just visible by the lighting in the whole beetle (Fig. 2i) but not in the enlarged view (Fig. 5m) and with no trace in the female, and the clypeus is elevated medially. The anterior margin of the clypeus is emarginate over its median 1/3 and the lateral angles of this emargination are bluntly rounded (Figs 5, 6). The lateral area of the frons, between the frontoclypeal suture and the eyes, bulges outwards as a pair of rounded genae whose outer margin may be a simple continuation of that of the clypeus, but may be distinctly angled outwards, as in female *A.isikdagensis* (Fig. 6b) The epipharynx (Figs 7–9) is of the type mainly adapted for saprophagy (Dellacasa et al. 2010), but retaining some features of the coprophagous type. The acropariae are sparse but fine, more extensively developed in some species (e.g., *A.plagiatus* (Linnaeus, 1767) (Fig. 7a–f) and almost absent in others (e.g. *A.isikdagensis*, Fig. 8c, d), the chaetopariae are stout but are fairly long and closely set, the prophobae and apophobae are present – the apophobae as lines of dense fine bristles lateral to the chaetopariae and the prophobae concentrated in the posterior part of the pedia generally clustered at the edges of the mesoepitorma. The number and position of the chaetopedia varies, both between and within species and may differ on the two sides of the same specimen (e.g., *A.wilsonae*, Fig. 9i). The pariae are rounded laterally and the clithra is emarginate either side of the median tylus, which has the corypha distinctly protruding. For morphology of the epipharynx see Dellacasa et al. (2010).

**Figure 10.**
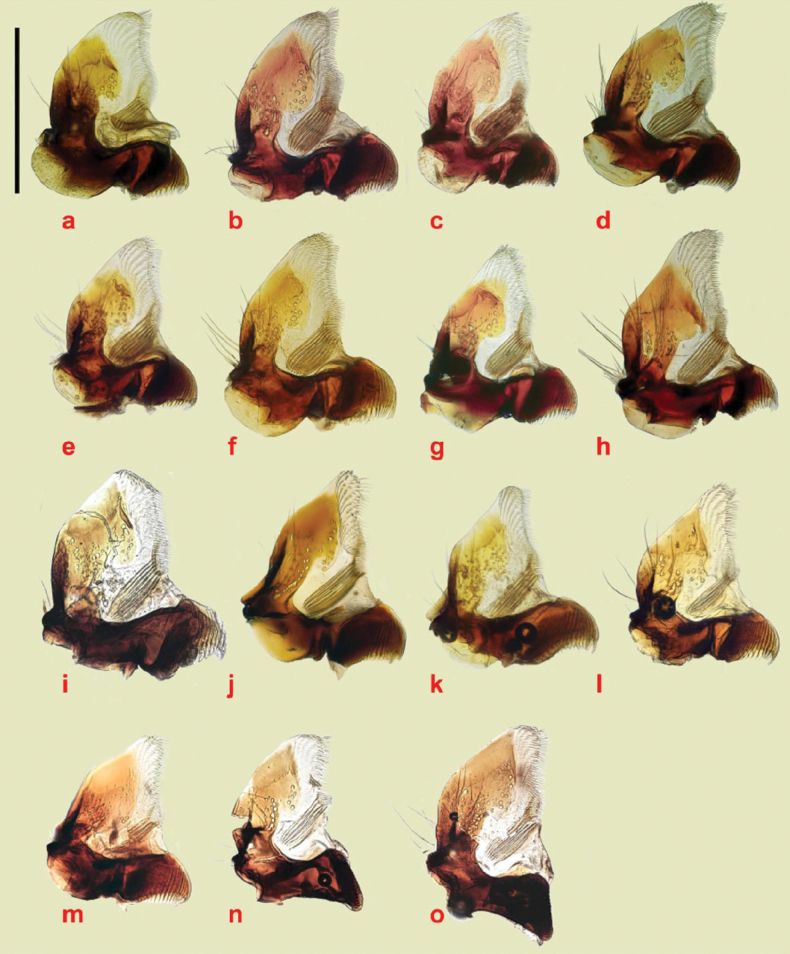
Mandibles **a***A.niger*, GB, Hampshire, New Forest **b***A.muscorum*, HU, Hortobagyi **c***A.felix* sp. nov., IT, Campo Felice **d***A.krelli* sp. nov., IT, Sardinia, Badde Salighes **e***A.alberti* sp. nov., TU, Ovit Dağı **f***A.bellumgerens* sp. nov., IT, Sicily, Piano Battaglia **g***A.bameuli* sp. nov., FR, Corsica, Haute-Corse **h***A.wilsonae*, SP, Cantabria, Corconte **i***A.rusakovi*, RU **j***A.kraatzi*, SK, Chlaba **k***A.plagiatus*, GB, Norfolk, Hunstanton **l***A.rodrigoi* sp. nov., SP, Madrid, Aranjuez **m***A.chellala* sp. nov., AG, Chellala **n***A.rutilipennis* sp. nov. **o***A.discoides* sp. nov. Scale bar: 0.5 mm.

The mandibles (Fig. 10) conform to the typical structure of most Aphodiini adapted to a soft saprophagous diet (sensu Stebnicka 1985), being triangular in shape and equally as long as they are wide, with a well-developed, flattened incisor lobe which extends over the apex of the callum laterale. The lateral edge of the mandible is strongly developed and sclerotised basally and can be regularly convex or almost (*A.niger* group, except for *wilsonae*, Fig. 10h), or sinuate (*A.plagiatus* group) depending on the width of the incisor lobe which varies in width and is much smaller in the *plagiatus* group taxa. The incisor lobe is broadly concave medially with pectinated edge and is clothed in long setae both in the distal end (pecten distalis) as well as the medial edge (pecten medialis) and ventral side of the apex of the callum laterale (pecten ventralis). In common with other closely related taxa in the “*Nialus* Mulsant & Rey, 1870” complex (sensu Rakovič 1991) their mandibles have well sclerotised basis and callum laterale with abundant and large glandular pores as well as a strong apical pore on the callum laterale that sits at the distal end of a strengthening ridge running more or less parallel to the medial edge of the callum. The length of this ridge is variable intra-specifically, from strong and well developed in the niger group (except *L.wilsonae*), to much reduced (*rodrigoi* crown group) or even absent (*plagiatus* group). The setae on the pecten distalis are particularly strong and well sclerotised compared to most other Aphodiini species and the pectination along the medial edge is also stronger than other groups of Aphodiini. The prostheca is reduced in length and it falls well short of the middle of the medial edge. Compared to other species in the *Nialus* complex, *Liothorax* has a well-developed molar lobe with rather pronounced tritors (sensu Hata and Edmonds 1983), and the prostheca can be much shorter than typical, barely reaching less than a 1/3 of the medial edge (*plagiatus* group) and with a poorly developed prosthecal comb. The conjuncta (filtrum) is much coarser, with wider and reduced number of ridges than in other groups, ranging from five to six in the *plagiatus* group and seven to nine in the *niger* group, with the highest number of ridges being found in the *niger*-crown group (*A.niger* Illiger, 1798, *A.muscorum* (Ádám, 1994), *A.felix* sp. nov., and *A.krelli* sp. nov.). For definitions of the morphology of the mandibles please refer to Nel and De Villiers (1988) and Hata and Edmonds (1983).

**Figure 11.**
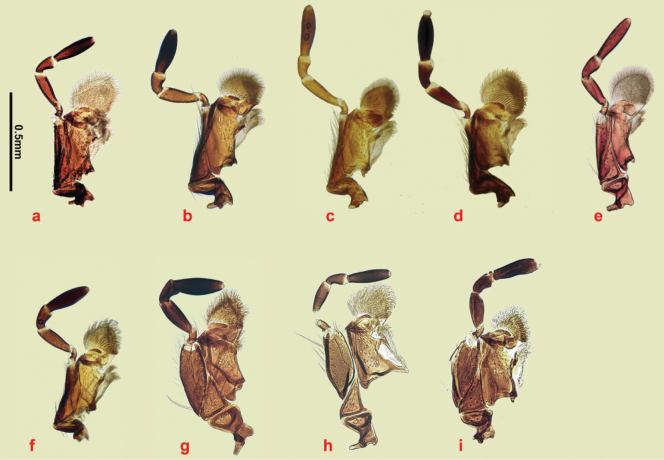
*Aphodiusplagiatus* group, maxillae **a***A.rusakovi*, KZ, Syr-Darja **b**, **c***A.kraatzi***b** CZ **c** RU **d***A.plagiatusplagiatus*, GB, Norfolk **e***A.p.sinoplagiatus* ssp. nov., paratype, CH, Qinghai, Gangca **f***A.rodrigoi* sp. nov. paratype SP Madrid, Aranjuez **g***A chellala*, paratype, AG, Chellala **h***A.rutilipennis* CY **i***A.discoides*, TR, Karacadag. Scale bar: 0.5 mm.

**Figure 12.**
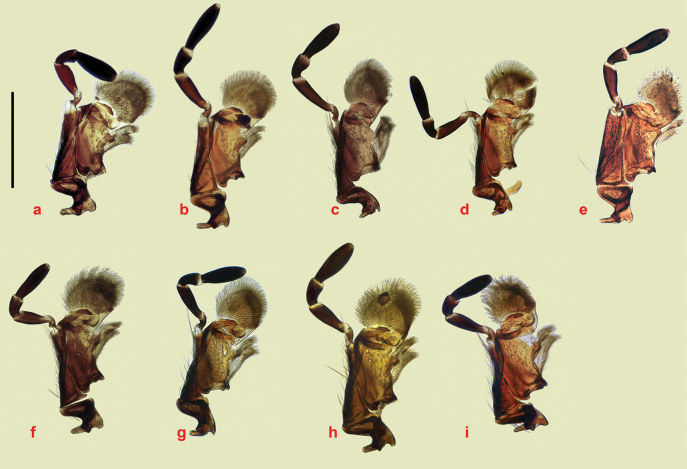
*Aphodiusniger* group, maxillae **a***A.niger*, GB, Hampshire, New Forest **b***A.muscorum* HU, Hortobagy **c***A.felix* sp. nov., paratype, IT, Campo Felice **d***A.krelli*, paratype, IT, Sardinia *A.muscorum*? CZ, Břeclav, Pohansko **e***A.isikdagensis*, paratype, TR, Ḉamildere **f***A.alberti*, sp. nov., paratype, TR *A.niger*? CZ, Hradec Králové **g***A.bellumgerens* sp.nov., paratype IT, Sicily *A.felix* sp. nov., paratype, IT, Sardinia **h***A.bameuli* sp. nov., paratype, FR, Corsica Sicily **i***A.wilsonae*, SP. Scale bar: 0.5 mm.

The maxillae (Figs 11, 12) of the *plagiatus* group are generally narrower than those of the *niger* group, and with the galeae generally smaller, barely produced laterally and with hooked setae on apex and medio-apical area and less dense setation.

**Figure 13.**
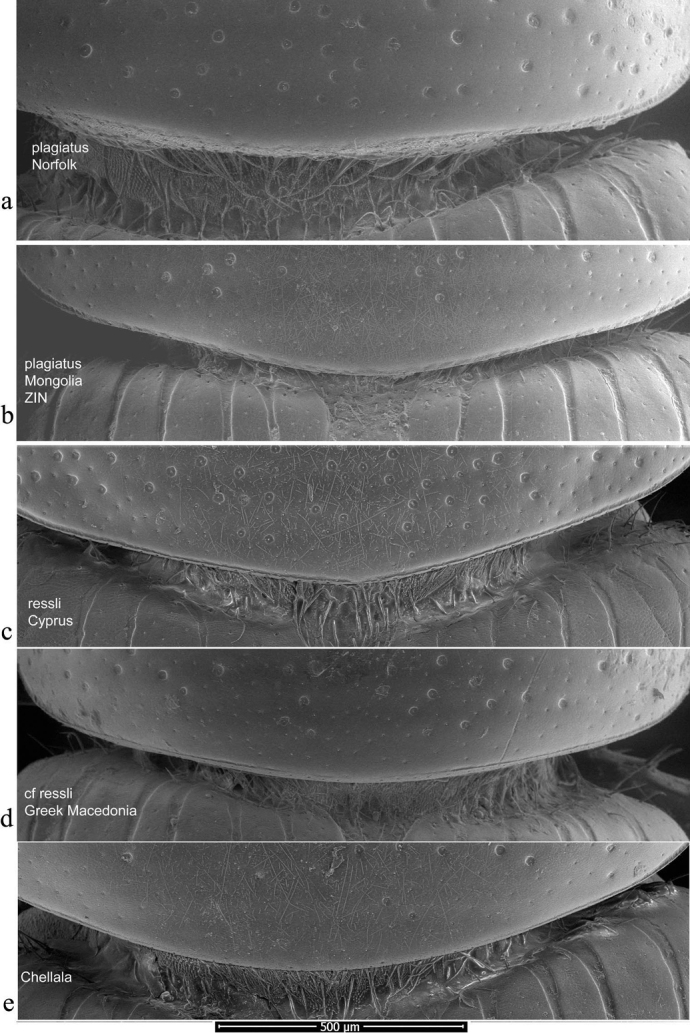
*Aphodiusplagiatus* group, pronotal bases 1 **a**, **b***A.plagiatus***a** GB, Norfolk **b** MG **c***A.rutilipennis*, CY **d**A.cf.rutilipennis, GR, Thessaloniki **e***A.chellala* sp. nov., paratype, AG.

**Figure 14.**
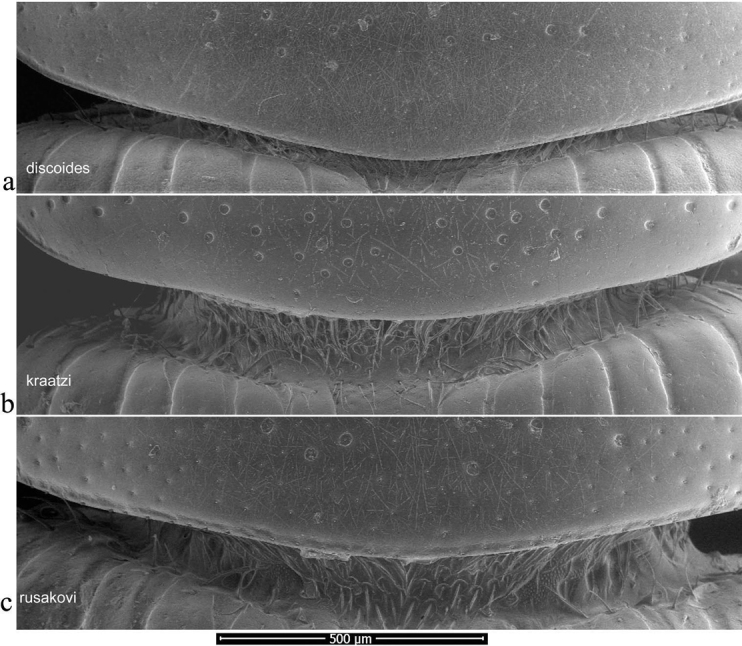
*Aphodiusplagiatus* group, pronotal bases 2 **a***A.discoides*, TR **b***A.kraatzi*, SK **c***L.rusakovi*, paratype, RU, Volgograd oblast’.

**Figure 15.**
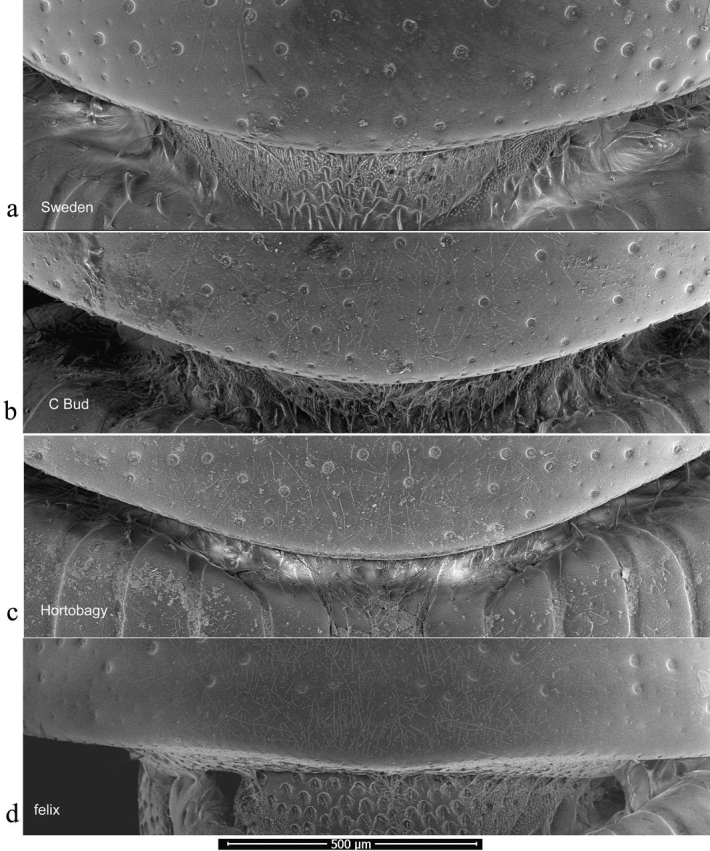
*Aphodiusniger* group, pronotal bases 1 **a***A.niger*, Sweden, Södermanland, Tullgarn **b**A.cf.niger, CZ, České Budějovice **c***A.muscorum*, HU, Hortobágyi **d***A.felix* sp. nov., paratype.

**Figure 16.**
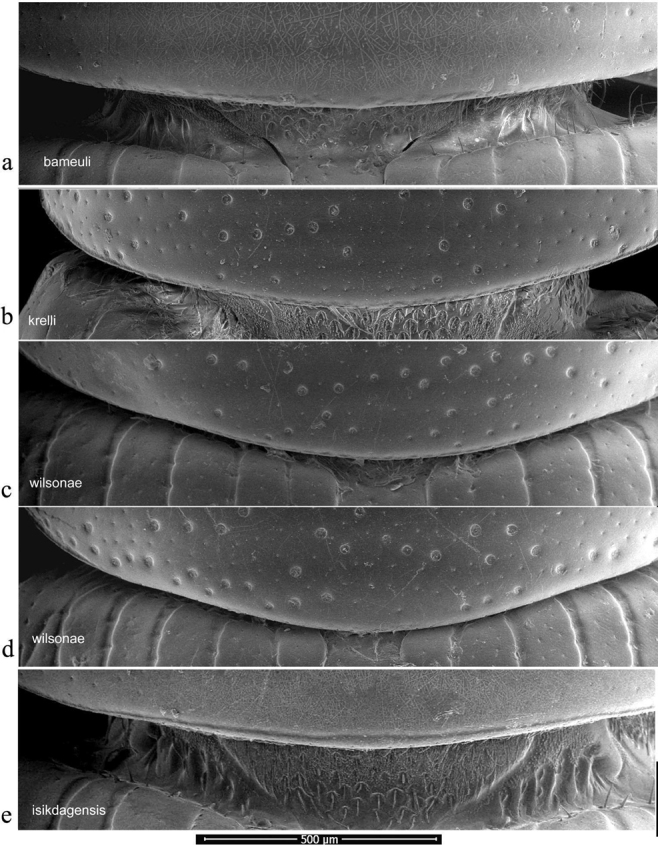
*Aphodiusniger* group, pronotal bases 2 **a***A.bameuli* sp. nov., paratype, FR, Corsica **b***A.krelli* sp. nov., paratype, IT, Sardinia **c**, **d***A.wilsonae*, paratypes, SP, Manzanares el Real **e***A.isikdagensis*, paratype, TR, Ḉamildere.

The pronotum is hemicylindrical, highly arched transversely but generally only weakly so longitudinally, and more or less parallel-sided (Figs 5, 6). The surface of the pronotum has a double punctation whose strength and density are very variable. The lateral margins are bordered and this border extends inwards round the hind angles to a variable extent. Thus, the hind margin may be entirely bordered, or the border may be narrowly or widely interrupted medially, or there may be an unbordered section either side of the bordered median area (Figs 13–16).

The scutellum is pentagonal, with the sides more or less parallel over their basal 1/2, then tapered to a point apically (Figs 5, 6).

**Figure 17.**
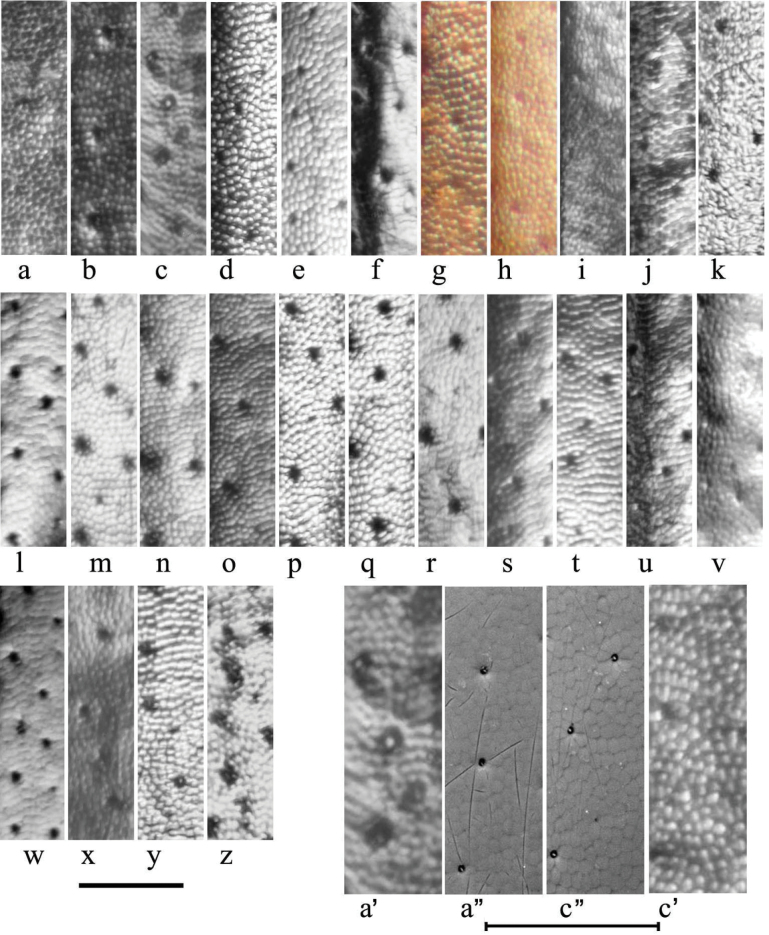
Elytral sculpture of interstices 2 or 3 **a**–**z** stacked photographs **a***A.plagiatusplagiatus*, CZ, Moravia **b**, **c***A.p.sinoplagiatus* ssp. nov., paratypes, CH **b** Gangca, Qinghai **c** Gansu **d***A.rutilipennis* (*A.ressli*, holotype), TR, Hatay **e***A.chellala* sp. nov., holotype **f***A.rusakovi*, paratype, RU, Orenburg **g**, **h***A.discoides***g** neotype, IS, Kuneitra **h** TR, Kizildag **i***A.discoides*, black specimen, TR, Karakurt **j***A.kraatzi*, SK **k***A.isikdagensis*, paratype, TR, Ḉamildere **l**, **m***A.niger*, Sweden, Södermanland, Tullgarn **n–q***A.muscorum*, HU **n** holotype, Vörös-tó **o** Hortobágyi **p**, **q** Kisjúszállás **r, s***A.niger*? **r** CZ, Hradec Králové **s** CZ, České Budějovice **t***A.muscorum*? SK, Bol **u***A.bellumgerens* sp. nov., paratype, IT, Sicily **v***A.felix* sp. nov., paratype, IT **w***A.wilsonae*, paratype, SP **x***A.bameuli* sp. nov., paratype, FR, Corsica **y***A.krelli* sp. nov., paratype, IT, Sardinia **z***A.alberti* sp. nov., paratype, TR, Rize, Ovitdag. **a’–c**’ *A.plagiatus*, at higher magnification **a**’, **c**’ stacked photographs **a**”, **c**” SEM images **a**’, **a**” *A.p.sinoplagiatus* ssp. nov., CH, Gansu **c**”, **c**’ *A.p.plagiatus*, CZ, Moravia. Scale bars: 0.1 mm.

The elytra are finely but distinctly striate with the interstices 6–10× wider than the striae, normally flat but sometimes weakly convex. The interstices may be distinctly but finely reticulate, in some species giving a slightly dull appearance (in contrast to the glossy striae), while in other species the elytra appear glossy black. The underlying reticulation in all cases is small-meshed isodiametric, but its strength varies both within and between species, and has caused considerable confusion (Fig. 17). Thus, in Britain at least, *A.plagiatus* has been distinguished from *A.niger* by its more obviously reticulate elytra, giving a somewhat matt appearance. While this is true of some populations of *A.plagiatus* e.g., from England and Moravia (Fig. 17a), in other populations (e.g. from Hungary and the St Petersburg district of Russia) the elytra are glossy black with the reticulation less prominent, while material from the Tibetan plateau has the elytra glossy with reticulation visible only under intense illumination and at high magnification (Fig. 17b, c). This necessity for high intensity illumination to reveal the reticulation suggested that perhaps the transparent epicuticle was smooth, with the reticulation a feature of the underlying exocuticle. However, scanning electron (SEM) micrographs show that the reticulation is in fact present on the epicuticle (Fig. 17a”, c”).

The raised bead round the apex of the elytra is well developed (Fig. 19a), comparable with that of Aphodius (Nialus) varians Duftschmid, 1805 (Fig. 19b), slightly narrower than the apical section of stria 2, clearly narrower than those of Aphodius (Calamosternus) granarius (Linnaeus, 1767) and A. (C.) hyxos (Petrovitz, 1962) where the bead is at least as wide as the stria and may be ca 2× its width (Fig. 19c, d). The bead is clearly wider than in species such as A. (Agoliinus) lapponum Gyllenhal, 1806 (Fig. 19e) and A. (Agrilinus) constans Duftschmid, 1805 (Fig. 19f).

The elytra have clearly angulate, rounded shoulders with a more or less developed denticle which can be variable in development.

**Figure 18.**
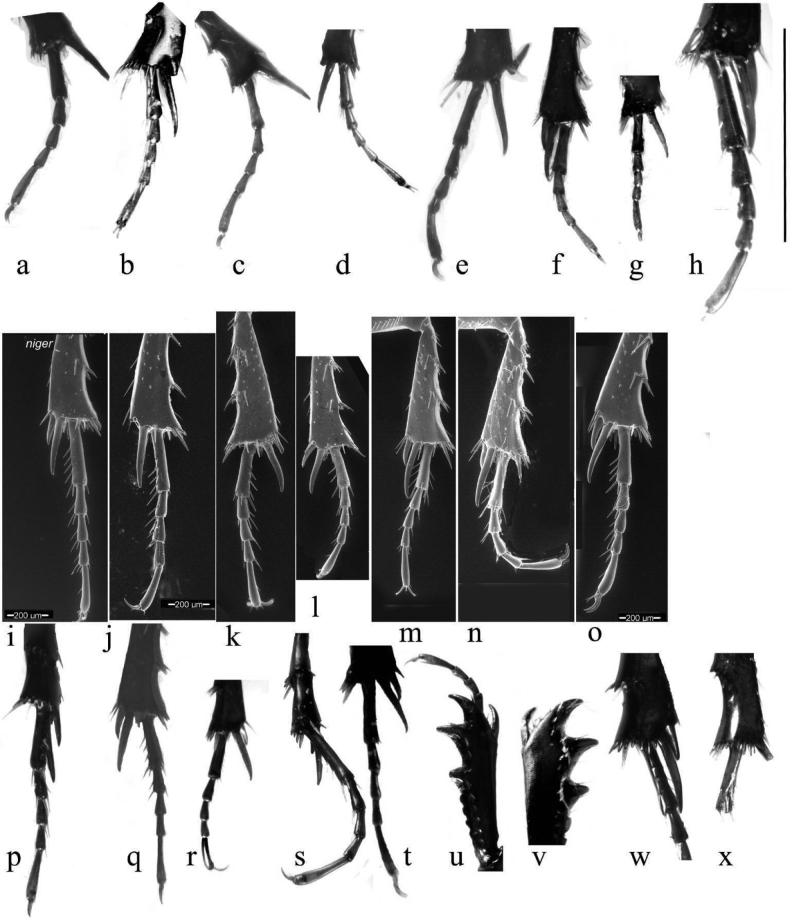
Legs **a–h***A.plagiatus* group, stacked photographs mid tarsi and apical portions of tibiae **a***A.plagiatussinoplagiatus* ssp. nov., paratype, CH, Gangca, Qinghai **b***A.rodrigoi* sp. nov. holotype, SP, Aranjuez **c***A.rutilipennis* (*A.ressli*, holotype), TR, Hatay **d***A.chellala* sp. nov., holotype, AG, Chellala **e***A.discoides*, neotype, IS, Keneitra **f**, **g***A.kraatzi***f** SV **g** RU, Karasuk, W. Siberia **h***A.rusakovi*, paratype ♂, RU, Orenburg **i**–**t***A.niger* group, mid tarsi, and tibial apices **i–o** SEM images **p–t** stacked photographs **i***A.niger*, Sweden, Södermanland, Tullgarn **j***A.muscorum*, HU, Hortobágyi **k**, **l***A.bameuli* sp. nov., paratypes, FR, Corsica **m**, **n***A.krelli* sp. nov., paratypes, IT, Sardinia **o***A.wilsonae*, paratype SP **p, q***A.felix* sp. nov, IT, Abruzzo, Campo Felice **p** paratype **q** holotype **r***A.bellumgerens* sp. nov., paratype, IT, Sicily **s***A.isikdagensis*, paratype, TR, Ḉamildere **t***A.alberti* sp. nov., holotype, TR, Rize, Ovitdag **u**, **v** fore tibiae and tarsi of ♂♂ to show the spur. **u***A.bameuli* sp. nov., paratype, FR, Corsica **v***A.rusakovi*, paratype, RU, Orenburg **w**, **x** apex of hind tibia to show the short spines **w***A.muscorum*, HU **x***A.wilsonae*, paratype, SP. Scale bar: 1 mm.

The small spines at the apex of the mid- and hind tibiae are short and fairly even in length (Fig. 18w, x). The apical spurs of the mid and hind tibiae are of varying length between the species and can be helpful in species identification, in particular in separating members of the *A.plagiatus* group of species, in which the basal segment of the mid tarsus is always clearly shorter than the longer tibial spur from the *A.niger* group of species in which the basal tarsal segment in some species is longer than the longer tibial spur, while in others the segment may be either shorter than or ca the same length as the spur (Fig. 18a–t). This character was first used by Ljungberg and Hall (2009) for separating Swedish *A.niger* and *A.plagiatus*.

**Figure 19.**
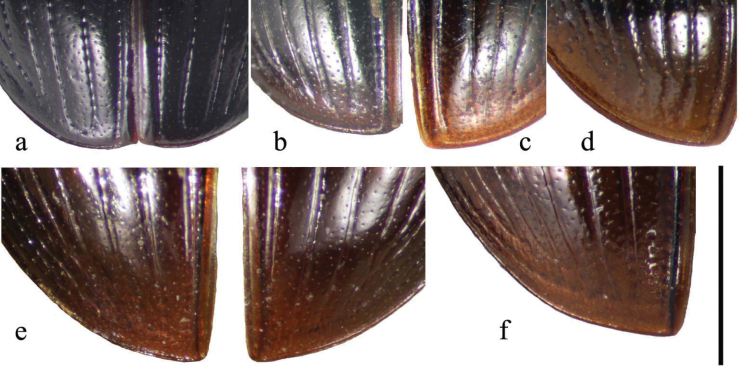
Elytral apices **a**A. (L.) niger, SV, Södermanland, Tullgarn **b**A. (*Nialus*) *varians*, FR, left elytron **c**A. (Calamosternus) granarius, CY, right elytron **d**A. (C.) hyxos, CY, left elytron **e**A. (Agoliinus) lapponum, GB, Cumbria, both elytra **f**A. (Agrilinus) constans, IT, Sardinia, left elytron. Scale bar: 1 mm.

**Figure 20.**
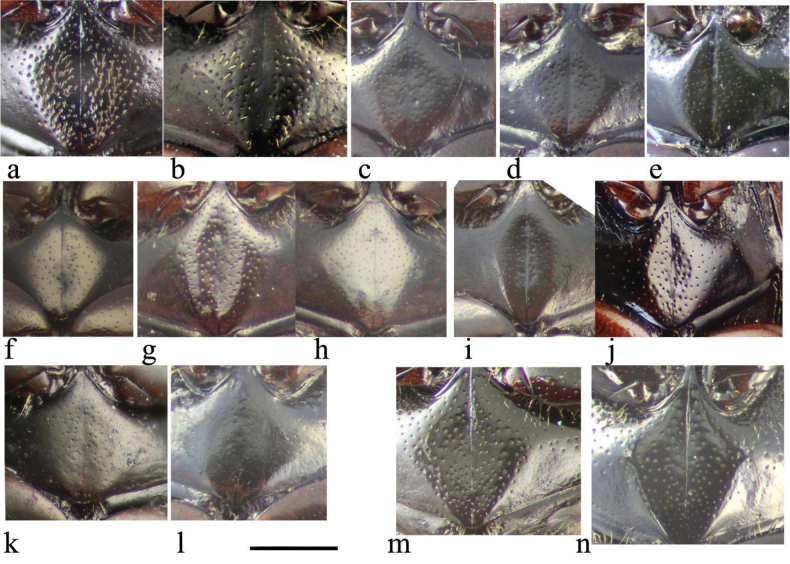
Metaventrites, *A.plagiatus* group **a***A.plagiatusplagiatus*, GB, Studland, ♂ **b***A.p.sinoplagiatus* ssp. nov., paratype, CH, Gansu, ♀ **c**, **d***A.rutilipennis*, CY **c** ♂ **d** ♀ **e**, **f***A.chellala* sp. nov., paratypes, AG **e** ♂ **f** ♀ **g**, **h***A.kraatzi*, RU, Orenburg, ♂ **i***A.kraatzi*, SK, ♀ **j***A.rodrigoi* sp. nov., holotype, ♂, SP, Provinicia de Madrid, Aranjuez **k**, **l***A.discoides***k** neotype ♂, IS, Kuneitra **l** TR, ♀ **m**, **n***A.rusakovi***m** paratype, ♂, RU, Orenburg **n** ♀ RU, Volgograd oblast’. Scale bar: 0.5 mm.

**Figure 21.**
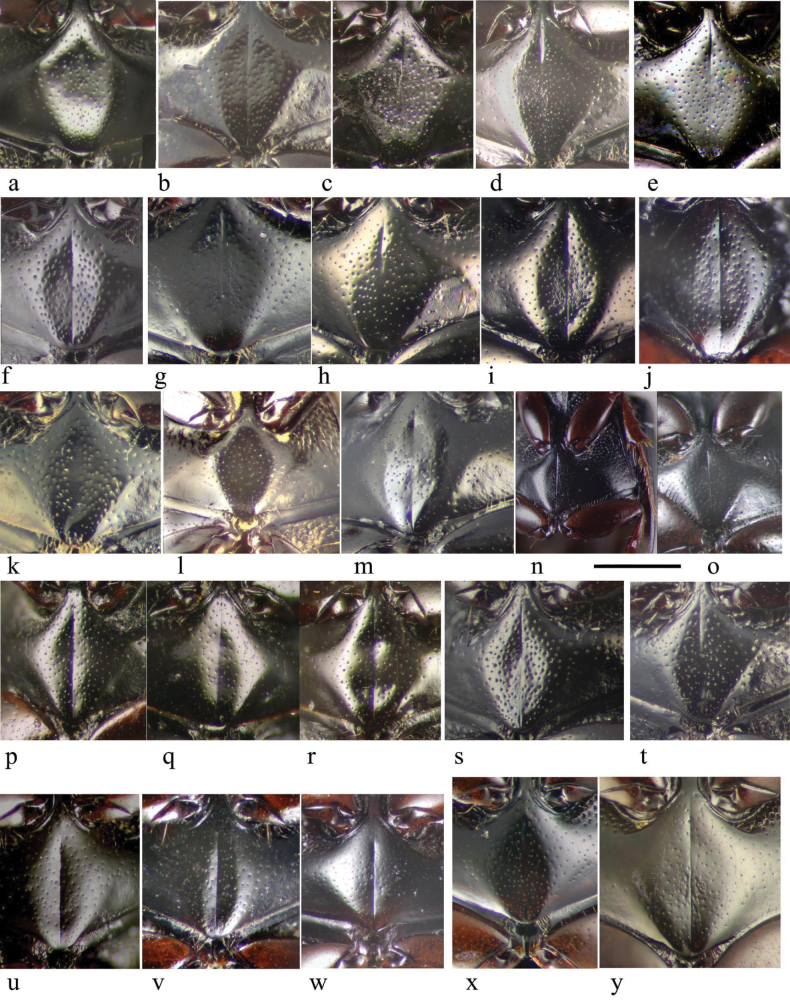
Metaventrites, *A.niger* group **a–d***A.niger***a**, **c**, **d** SV, Södermanland, Tullgarn **b** GB, Hampshire, New Forest **a**, **b** ♂ **c**, **d** ♀ **e**, **g–j***A.muscorum***e** holotype ♀, HU, Vörös-tó **f***A.felix* sp. nov., paratype ♂, IT, Campo Felice **g** ♂, HU, Hortobágyi **h**, **i** ♀♀, HU, Kisujszallas **j** ♀, HU, Josvafo **k***A.felix* sp. nov. paratype ♀, IT, Campo Felice **l**, **m***A.bellumgerens* sp. nov., paratypes, IT, Sicily, Piano Battaglia **l** ♂ **m** ♀ **n**, **o***A.alberti* sp. nov., paratypes, TR, Rize, Ovitdag **n** ♂, **o** ♀ **p**–**r***A.bameuli* sp. nov., paratypes, FR, Corsica **p** ♂ **q**, **r** ♀ **s**, **t***A.krelli* sp. nov., paratypes, IT, Sardinia **s** ♂ **t** ♀ **u**–**w***A.wilsonae*, paratypes **u**, **v** SP, Manzanares el Real **u** ♂ **v** ♀ **w** ♀, SP, El Vellon **x**, **y***A.isikdagensis*, paratypes, TR, Ḉamildere **x** ♂ **y** ♀. Scale bar: 0.5 mm.

The sculpture of the metaventrites (Figs 20, 21) may be helpful in recognising species. Particularly striking is the ventrite of male *A.plagiatus*, closely and heavily punctate with the punctures bearing yellow setae. Otherwise, the extent of any reticulation over the median part of the ventrite can be useful.

Following the account of aedeagal structure given by Lawrence and Ślipiński (2013: 61) the phallobase is sclerotised ventrally, and the parameres in the *A.niger* group are turned upwards at the apex. However, for practical purposes it seems more useful to refer to the orientation of the aedeagus when extruded backwards from the apex of the abdomen, with the parameres downturned apically in the *A.niger* group, and with the phallobase sclerotised on its upper surface (Fig. 23f). This means that the right and left sides refer to their positions within the abdomen.

**Figure 22.**
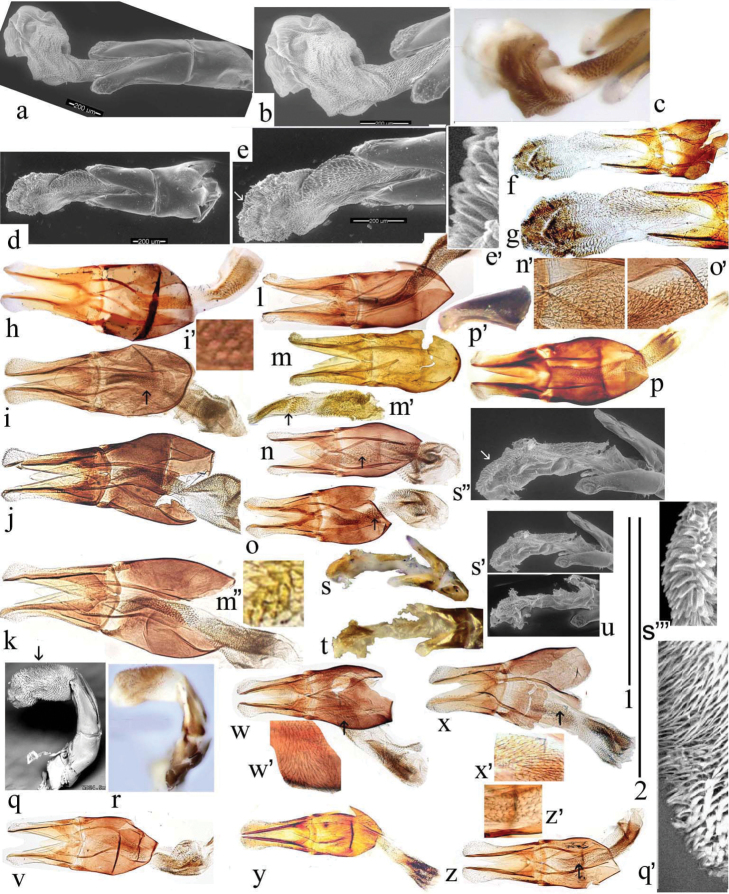
*Aphodiusplagiatus* group aedeagi **a–c***A.plagiatusplagiatus*, GB, Studland, Dorset, aedeagus with extruded endophallus **a**, **b** SEM images **a** whole aedeagus **b** endophallus in more detail **c** stacked photograph of endophallus **d–g***A.discoides*, neotype (*Aphodiusbytinskisalzi*, holotype, IS, Kuneitra) **d**, **e**, **e**’ SEM images **f**, **g** stacked photographs **h–n** cleared preparations mounted on slides **h***A.p.plagiatus*, GB, Norfolk **i***A.p.sinoplagiatus*? “China, Pekin”, Fry collection **i**’ teeth on neck of endophallus in the region marked with an arrow in **i j**, **k***A.plagiatussinoplagiatus* ssp. nov., paratypes **j** CH, Gangca **k** CH, Tsaidam **l***Aphodiusjakutorum* Balthasar, lectotype, RU, East Siberia **m***A.rodrigoi* sp. nov., holotype, SP, Aranjuez, dissected aedeagus **m**’ endophallus **m**” endophallic teeth in area indicated by arrow in **m**’ at higher magnification **n***A.discoides*, TR, Karacadag **n**’ teeth on neck of endophallus, in area indicated by arrow in **n** at higher magnification **o***A.discoides*, black specimen from TR, Karakurt, **o**’ teeth on neck of endophallus, in area indicated by arrow in **o** at higher magnification **p**, **p**’ *A.rusakovi***p** paratype, RU, Orenburg **p**’ RU Volgograd obl. Fastov, paramere, lateral **q–v** SEM images and stacked photographs **q**, **r***A.rutilipennis*, CY, with endophallus inflated **q** SEM image **q**’ (bottom right of plate) dorsal surface of endophallus in area indicated by arrow in **q** at higher magnification, to show the hair-like setae **r** stacked photograph of the same specimen **s**, **s**’ *A.chellala* sp. nov., paratype with endophallus extruded **s** stacked photograph **s**’, **s**”, **s**”’ SEMs of the same specimen **s**”, **s**”’ at higher magnifications **t**, **u***L.chellala* sp. nov., holotype with the endophallus extruded **t** stacked photograph **u** SEM image **v**–**z** photographs of cleared specimens mounted in DMHF on slides **v**, **w***A.rutilipennis***v** CY **w** TR **w**’ teeth on neck of endophallus, in area indicated by arrow in **w** at higher magnification **x***A.chellala* sp. nov., paratype, AG, Taguin (La Smala) **x**’ teeth on neck of endophallus, in area indicated by arrow in **x** at higher magnification **y**, **z***A.kraatzi***y** KZ, Syr Darya **z** AF, Kabul **z**’ teeth on neck of endophallus, in area indicated by arrow in **z** at higher magnification Scale bar 1: 1 mm (**a**, **d**, **f**–**t**, **v**–**z**); 0.4 mm (**n**’, **o**’, **s**”, **w**’, **x**’, **z**’); 0.17 mm (**e**’, **m**”, **q’ s**”’). Scale bar 2: 1 mm (**b**, **c**, **e, g** and **s**”).

**Figure 23.**
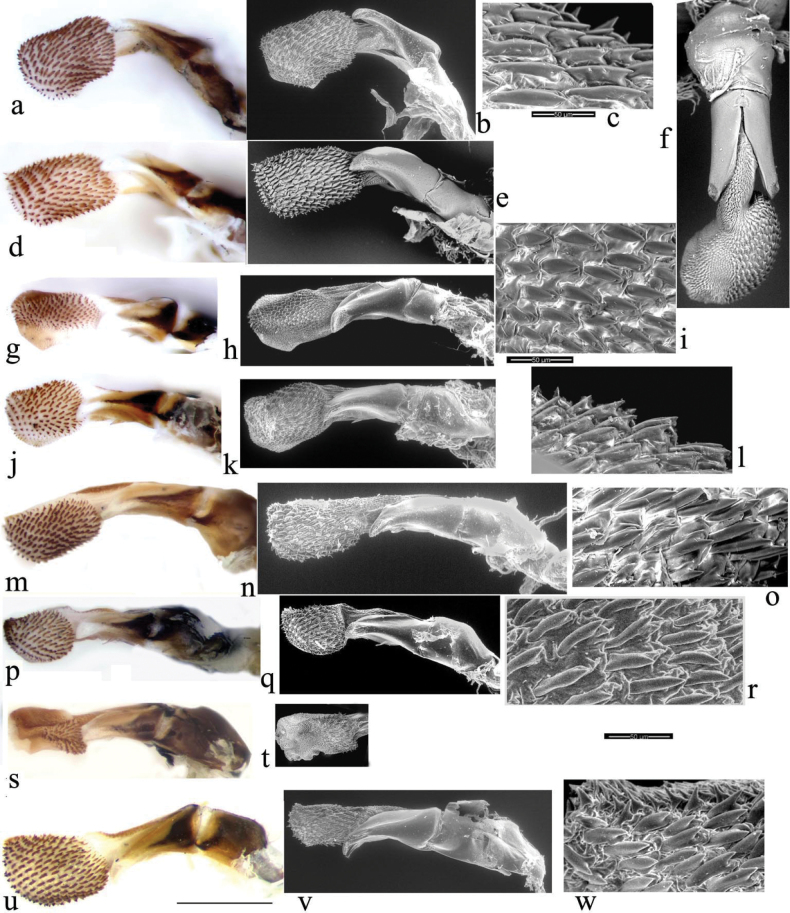
*A.niger* group, aedeagi with inflated endophalli, stacked photographs and SEM images **a–e, g–w** right lateral view **f** viewed from above **a–c***A.niger*, SV, Södermanland, Tullgarn **a** stacked photograph **b** the same aedeagus, SEM image **c** detail of endophallic teeth **d–f***A.niger*, GB, Hampshire, New Forest **d** stacked photograph **e**, **f** SEM images **g–i***A.wilsonae*, SP, Areños **g** stacked photograph **h** SEM image, **i** detail of endophallic teeth **j–l***A.bameuli* sp. nov., holotype **j** stacked photograph **k** SEM image **l** detail of endophallic teeth **m–o***A.krelli* sp. nov., holotype **m** stacked photograph **n** SEM image **o** detail of endophallic teeth **p–r***A.bellumgerens* sp. nov., paratype **p** stacked photograph **q** SEM image **r** detail of endophallic teeth **s**, **t***A.bellumgerens* sp. nov., holotype **s** stacked photograph **t** SEM image of endophallus **u***A.muscorum*, HU, Hortobágyi, stacked photograph **v**, **w***A.felix* sp. nov., holotype, SEM images **v** aedeagus **w** detail of endophallic teeth. Black Scale bar: 0.5 mm for aedeagi and endophallus, white SEM scale on black background: 50 μm for details of endophallic teeth.

**Figure 24.**
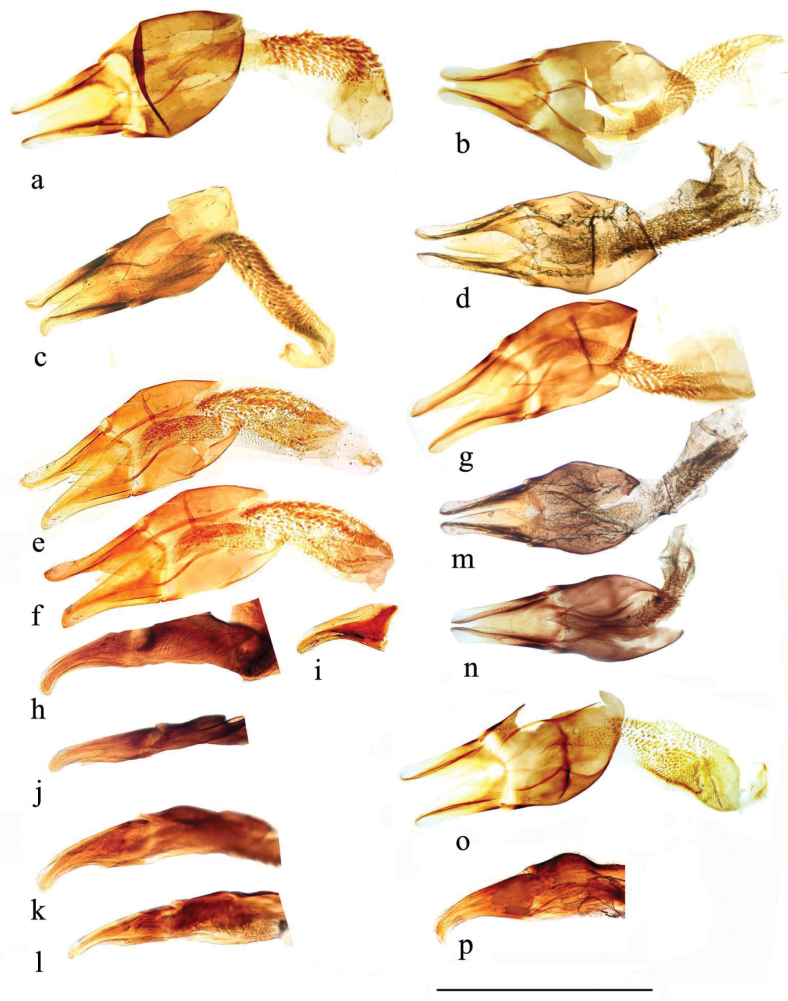
*A.niger* group aedeagi, cleared preparations mounted in DMHF on slides **a–g**, **m–o** dorsoventral view **h–l**, **p** right lateral view **a***A.niger*, SV, Södermanland, Tullgarn **b***A.bameuli* sp. nov., paratype **c***A.isikdagensis*, paratype, TR, Ḉamildere **d***A.krelli* sp. nov., paratype, IT, Sardinia **e**–**f***A.alberti* sp. nov., TR, Rize, Ovitdag **e** paratype, after squashing **f** the same specimen, before squashing **g**, **h**, **j***A.alberti* sp. nov., different paratypes **i***A.alberti* sp. nov., paratype, paramere **k***A.isikdagensis*?, TR, Artvin **l***A.alberti*?, Armenia **m***A.bellumgerens* sp. nov., paratype, IT, Sicily, Piano Battaglia **n***A.bellumgerens*, paratype IT, Sicily, Nebrodi **o***A.wilsonae*, paratype, SP, Manzanares el Real **p***A.muscorum*, HU, Hortobági. Scale bar: 1 mm.

The aedeagus has its endophallus armed with teeth, spines, and bristles, both on the neck of the endophallus (which in inflated specimens never protrudes beyond the paramere apices), and on the main section. The armature of the main section may be used to divide the genus into two sections, the *plagiatus* group with various spines and bristles and the *niger* group with an area of strong recurved thorn-like teeth on its right side. The main field of bristles in *A.rutilipennis* Baudi di Selve, 1870 also lies on the upper right side of the endophallus but the inflated endophallus of *A.chellala* sp. nov. is too damaged for this to be ascertained, and the location of the spines and bristles on the endophalli of *A.plagiatus* and *A.discoides* (A. Schmidt, 1916) is not clear from these rumpled endophalli (Figs 22–24). The parameres of the two sections are also shaped differently. In the *plagiatus* group the parameres are more or less straight and in dorsoventral view of cleared preparations there is a sclerotised strut running to the inner apical corner, lateral to which is a soft sensory area whose shape can be informative (Fig. 22). In the *niger* group the parameres are downturned apically and in dorsoventral view the sensory area is generally not visible, though it is visible in lateral view (Fig. 23) In some species this down-turning is weak and if the parameres are flattened, either by squashing on a slide, or by collapse on drying, the sensory area may become visible in dorsoventral view as a curved band across the parameres apex (Fig. 24).

**Figure 25.**
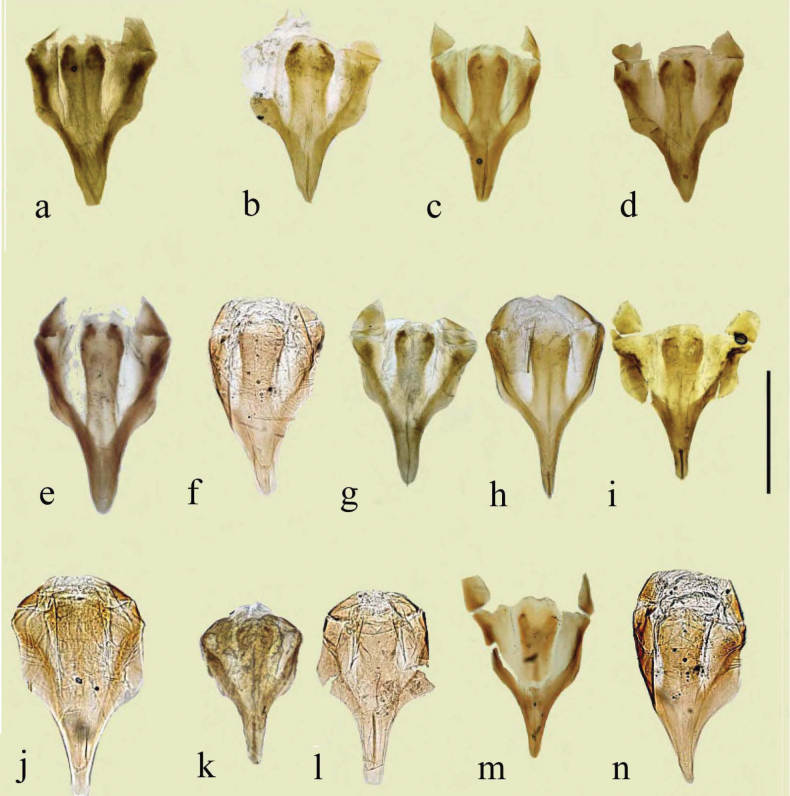
Aedeagal encasements (9^th^ abdominal sternite) **a***A.niger*, GB, Hampshire, New Forest **b***A.muscorum*, HU, Hortobagyi **c***A.felix* sp. nov., IT, Campo Felice **d***A.alberti* sp. nov., TU, Ovit Dağı **e***A.bellumgerens* sp. nov., IT, Sicily, Piano Battaglia **f***A.bameuli* sp. nov., FR, Corsica, Haute-Corse **g***A.wilsonae*, SP, Cantabria, Corconte **h***A.kraatzi*, RU, Chita Reg. **i***A.plagiatus*, UK, Norfolk, Hunstanton **j***A.plagiatus*, CN, Beijing **k***A.rodrigoi* sp. nov., SP, Madrid, Aranjuez **l***A.chellala* sp. nov., AG, Chellala, paratype **m***A.rutilipennis* sp. nov., CY, Konakli, paratype **n***A.discoides* sp. nov., paratype. Scale bar: 0.5 mm.

The sternite of the aedeagal encasement (Fig. 25) shows variation in the central spike, which tends to be narrower in the *A.plagiatus* group.

**Figure 26.**
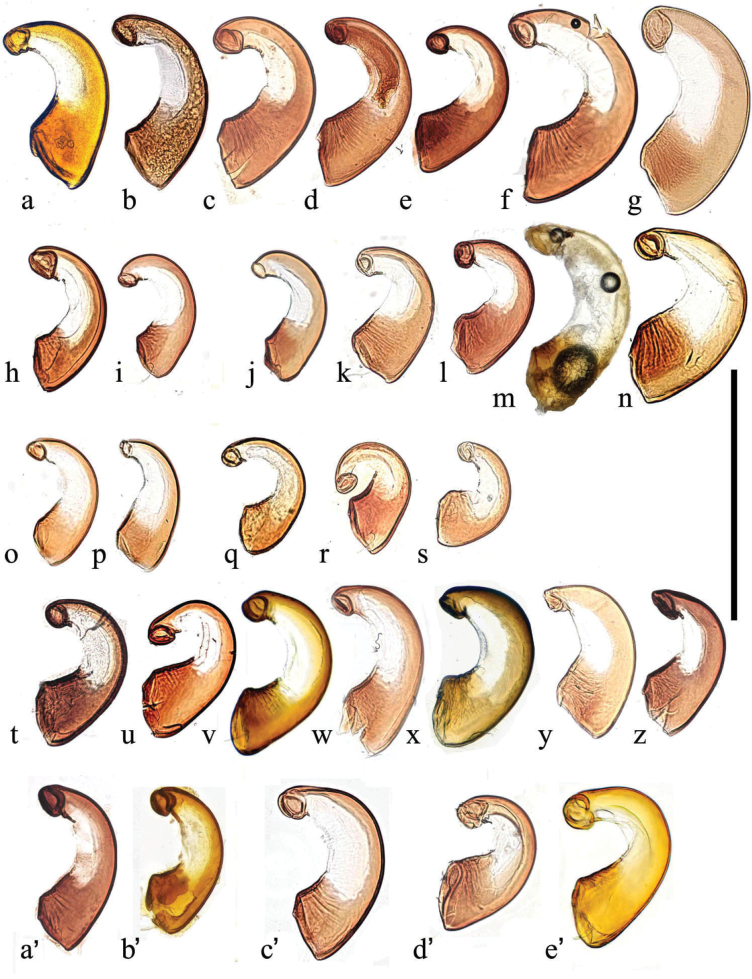
Spermathecae **a–e***A.plagiatusplagiatus***a** CZ, Dobre Pole **b** GB, Norfolk **c** KZ, Karaganda **d**, **e** RU, Transbaikal, Ulan Ude **f**, **g***A.p.sinoplagiatus*, CN **f** Gansu **g** holotype, CN, Qinghai **h**, **i**A.discoides, TR **h** Kizil dag, **i** Muʂ **j***A.rutilipennis* CY, Limassol distr **k***A.rutilipennis*?, GR Thesaloniki **l***A.chellala*, paratype, AG, Chellala **m**, **n***A.rusakovi***m** paratype, RU, wet but not covered **n** KZ, Syr-Darja **o–s***A.kraatzi***o**, **p** SK **q** RU, Astrakhan **r** RU, Lenkoran **s** AF, Kabul **t***A.niger*, GB, Hampshire, New Forest **u***A.muscorum* HU, Hortobágyi **v***A felix*, paratype, IT **w**, **x***A.bameuli*, paratypes, FR **y***A.krelli*, sp. nov., IT **z***A.bellumgerens* sp. nov., paratype, IT **a**’, **b**’ *A.alberti* sp. nov., TR paratypes **c**’ *A.isikdagensis*, paratype, TR Ḉamildere **d**’, **e**’ *A.wilsonae*, SP **d**’ paratype, SP, Madrid **e**’ SP, Soria. Scale bar: 0.5 mm.

The spermathecae (Fig. 26) have a simple curved (comma-shaped) form and their sizes may be useful in species (or subspecies) recognition.

Secondary sexual characters are variable and generally only weakly developed. The metaventrite may be flattened or concave medially in males, as against more rounded in females, but this is not always the case (Figs 20, 21). In *L.plagiatus* the metaventrite is flat and rather strongly punctate and bears well-developed yellowish setae in males while in females it is more arched medially and the punctures and setae are finer and the setae are often lost (Fig. 20a, b). Males may have the apical spur of the fore tibiae stronger than in females, and sometimes curved (Fig. 18u, v).

### ﻿Species recognised in this revision

**The *A.plagiatus* group**

A. (L.) plagiatus (Linnaeus, 1767)

*plagiatusplagiatus* (Linnaeus, 1767)

= *jakutorum* Balthasar, 1938)

= *hungaricus* Endrődi, 1955

*plagiatussinoplagiatus* subsp. nov.

A. (L.) rodrigoi sp. nov.

A. (L.) discoidesA. Schmidt, 1916, stat. rest.

= *discus* Reitter, 1892

= *bytinskisalzi* Petrovitz, 1971

A. (L.) rutilipennis Baudi di Selve, 1870, stat. rest.

= *ressli* Petrovitz, 1962

= *cypricola* Balthasar, 1971

A. (L.) chellala sp. nov.

A. (L.) kraatzi Harold, 1868

A. (L.) rusakovi Gusakov, 2004

**The *A.niger* group**

A. (L.) niger Illiger, 1798

A. (L.) muscorum Ádám, 1994, rest.

A. (L.) felix sp. nov.

A. (L.) bellumgerens sp. nov.

A. (L.) bameuli sp. nov.

A. (L.) krelli sp. nov.

A. (L.) isikdagensis Balthasar, 1953

A. (L.) alberti sp. nov.

A. (L.) wilsonae Angus & Maté, 2005, stat. rest.

### ﻿Key to the *Liothorax* species included in this paper

The key is suitable for males and series including both sexes. It is not suitable for females only because females of some species cannot be separated by morphological features.

**Table:** 

1	Parameres downturned apically, viewed from above either not showing the apical sensory area (Fig. 23a, b, d, g, m–o) or with it extending over the apical margin from the tip of the dark strut to the outer margin of the apex (Fig. 24c, e), Endophallus with strong recurved teeth (Figs 23, 24). Basal segment of mesotarsus either longer or ca the same length as the longer mesotibial spur (Fig. 18i–t) (the *niger* group)	**2**
–	Parameres not downturned apically, viewed from above with more or less transparent sensilla-rich pads running along the apical 1/3 to 1/2 of their outer margins (Fig. 22h–n, v–z). Endophallus with various hairs and bristles, never with strong recurved teeth (Fig. 22). Basal segment of mesotarsus always clearly shorter than the longer mesotibial spur (Fig. 18a–h) (the *plagiatus* group)	**10**
2	Specimens from Anatolia and the Trans Caucasus. Parameres in lateral view often only weakly downturned (Fig. 24h–l, p), and, viewed from above, with the apical sensory area often running across the apex, from the tip of the darkened strut to the outer apical angle (Fig. 24c, e)	**3**
–	Specimens from Europe, Kazakhstan, or western Siberia. Parameres in lateral view generally more strongly downturned (Fig. 24p) and, viewed from above, with a darkened strut running along their inner margins to the apex, their apices lateral to this transparent, not showing the sensilla-rich pads (Fig. 24a, b, d, g, m-o)	**4**
3	Larger species, length 4.5–5.8 mm. Legs mid brown (Fig. 3c, d). Epipharynx (Fig. 8c, d), with apophobae arranged in narrow bands ca 2 bristles wide. Turkey, Ḉamildere, Isik Dag mountain	**A. (L.) isikdagensis**
–	Smaller species, length 4.1–4.9 mm. Legs very dark brown to glossy black (Fig. 3e–h). Epipharynx (Fig. 8k, l) with the median darkened area narrowed over basal 1/4 and apophobae arranged in single rows. Turkey	***A* . (*L* .) *alberti* sp. nov.**
4	Basal segment of mesotarsus always distinctly longer than longer mesotibial spur. Length of longest teeth of endophallus ca 55–65 μm.	**5**
–	Basal segment of mesotarsus usually slightly shorter than longer mesotibial spur. Length of longest teeth of endophallus ca 35–55 μm. Y chromosome short, dot-like. If slightly longer, largely heterochromatic	**6**
5	Y chromosome long, metacentric, euchromatic (Fig. 31a, b, d, e). Elytra glossy black, though interstices have fine reticulation. Central part of metaventrite without reticulation (Fig. 21a–d)	**A. (L.) niger**
–	Y chromosome short, almost dot-like (Fig. 31f–h). Elytral interstices often with fine but distinct reticulation, giving the surface a leaden-grey silky sheen. Metaventrite of females variously depressed medially, depressed area often partly reticulate (Fig. 21e, g–j). Hungary	**A. (L.) muscorum**
6	Endophallic teeth conspicuously shorter than in all other species, ca as high as long. Length of longest teeth ca 35 μm (Fig. 23g–i, 24o). Iberian Peninsula	***A.* (*L.*) *wilsonae***
–	Endophallic teeth clearly longer than high. Length of longest endophallic teeth at least 40 μm	**7**
7	Pronotal punctation generally rather sparse and fine (Fig. 6g). Median plate of metaventrite more finely and sparsely punctured (Fig. 21p–r). Corsica	**A. (L.) bameuli sp. nov.**
–	Pronotal and/or metaventral punctation stronger	**8**
8	Tooth-field of endophallus approximately as long as parameres (Figs 23p–t, 24m, n). Pronotal punctation generally strong, that of metaventrite strong or weak. Sicily	**A. (L.) bellumgerens sp. nov.**
–	Endophallic tooth-field longer than parameres. Pronotal and metaventral punctation generally strong	**9**
9	Lateral near vertical area of pronotal surface bulging in posterior 1/3, so lateral margins in this area not visible from above (Fig. 6i). Central Apennines (Campo Felice)	**A. (L.) felix sp. nov.**
–	Lateral near vertical area of pronotal surface less bulging, lateral margins entirely visible from above (Fig. 6h). Sardinia	**A. (L.) krelli sp. nov.**
10	Median plate of metaventrite more or less flat in males, weakly arched in females, strongly punctate, the punctures bearing yellow recurved setae, these particularly conspicuous in males. In some females the punctures are a bit weaker, and in females the setae seem more easily lost (Fig. 20a, b). (Caution: these setae are easily lost, especially through attempts to clean dried specimens). Most specimens (from Europe, Siberia, and Mongolia) with the elytral interstices distinctly but finely reticulate, often giving a leaden-grey sheen, and with the pronotal base bordered by a more or less continuous fine impressed line, but some (subspecies *L.p.sinoplagiatus* from the Tibetan Plateau) with the elytra glossy black with the reticulation difficult to see, and the basal pronotal impressed line broadly interrupted medially. Elytra slightly rounded at sides. The most widespread species, from Europe to eastern Siberia and China	**A. (L.) plagiatus**
–	Median plate of metaventrite without yellow setae, usually more finely punctured	**11**
11	Rather elongate beetles with the outer margins of the elytra, viewed from above, rather straight and parallel-sided (Figs 2i, 3a, b)	**12**
–	Less elongate beetles with the outer margins of the elytra more rounded (Fig. 2a–h, j, k)	**13**
12	Larger, length 4.5–6.5 mm. Base of pronotum completely bordered by a fine impressed line, this sometimes weaker medially (Fig. 14c). Protibial spur of male strongly incurved apically (Fig. 18v). ♀ with spermatheca larger (Fig. 26m, n), length ca 0.35 mm. Russia, Orenburg and Volgograd districts, eastern Ukraine	***A* . (*L* .) *rusakovi***
–	Smaller, length 3.7–4.8 mm. Basal line of pronotum completely effaced over middle 1/3 (Fig. 14b). Beetles often appearing very narrow. ♀ with spermatheca smaller (Fig. 26o–s), length ca 0.25 mm. Dry sandy areas eastwards from southern Slovakia to Middle Asia, Transbaikal, and Afghanistan	**A. (L.) kraatzi**
13	Base of pronotum with a continuous fine but distinct groove (Fig. 2d). Metaventrite with median diamond-shaped area moderately punctate, depressed medially (Fig. 20j). Aedeagus (Fig. 22m, m’, m”) with the scales of the basal portion longer than in any other member of the *L.plagiatus* group (length 19–26 µm in *rodrigoi* vs 14–21 µm in the others.) Central Spain.	**A. (L.) rodrigoi sp. nov.**
–	Base of pronotum with fine groove interrupted by at least the width of one larger pronotal puncture (Fig. 13c, d), but frequently more widely interrupted	**14**
14	Width of median interruption of basal pronotal groove normally ca equal to diameter of one larger pronotal puncture, but occasionally wider, equal to ca 5 of the larger punctures (Fig. 13c, d). Cyprus, southern Anatolia, Greece, Albania.	***A* . (*L* .) *rutilipennis***
–	Basal groove of pronotum broadly interrupted medially	**15**
15	Small black species from Algeria. Aedeagus with endophallus with some elongate scales (aedeagus Fig. 22s–u, x)	**A. (L.) chellala sp. nov.**
–	East Mediterranean species. Elytra usually dirty yellowish on disc, but may be black. Aedeagus with endophallus without bristles or elongate scales (aedeagus Fig. 22d–g, n, n’)	**A. (L.) discoides**

**Figure 27.**
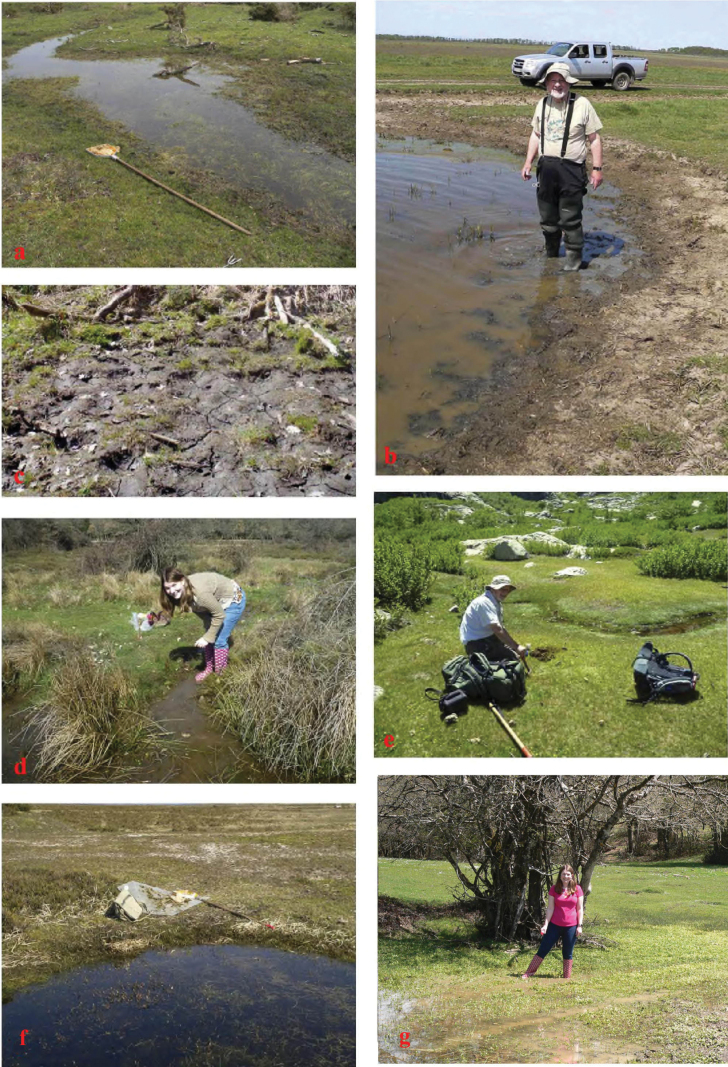
Habitat pictures **a***A.niger*, GB, Hampshire, New Forest **b***A.muscorum*, HU, Hortobagyi **c***A.niger*, SW, Tullgarn **d***A.krelli* sp. nov., IT, Sardinia, Badde Salighes **e***A.bameuli* sp. nov., FR, Corsica, Haute-Corse, Lac de Melo **f***A.wilsonae*, SP, Cantabria, Corconte **g***A.bellumgerens* sp. nov., IT, Sicily, Nebrodi.

**Figure 28.**
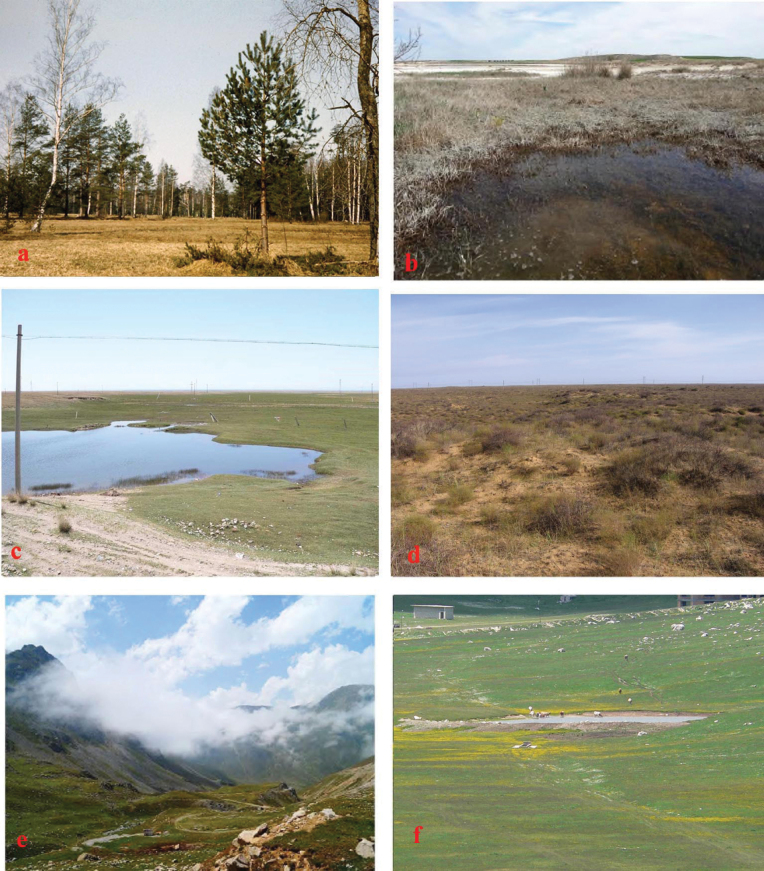
Habitat pictures **a***A.plagiatus*, RU, Pavlosk Park **b***A.rodrigoi* sp. nov., SP, Toledo, Quero **c***A.p.sinoplagiatus*, CN, Tibet, Ganca **d***A.kraatzi*, RU, Astrakhan oblast’ **e***A.alberti* sp. nov., TU, Ovit Dağı (photograph Copyright Yunus Yasuv) **f***A.felix* sp. nov., IT, Campo Felice.

**Figure 29.**
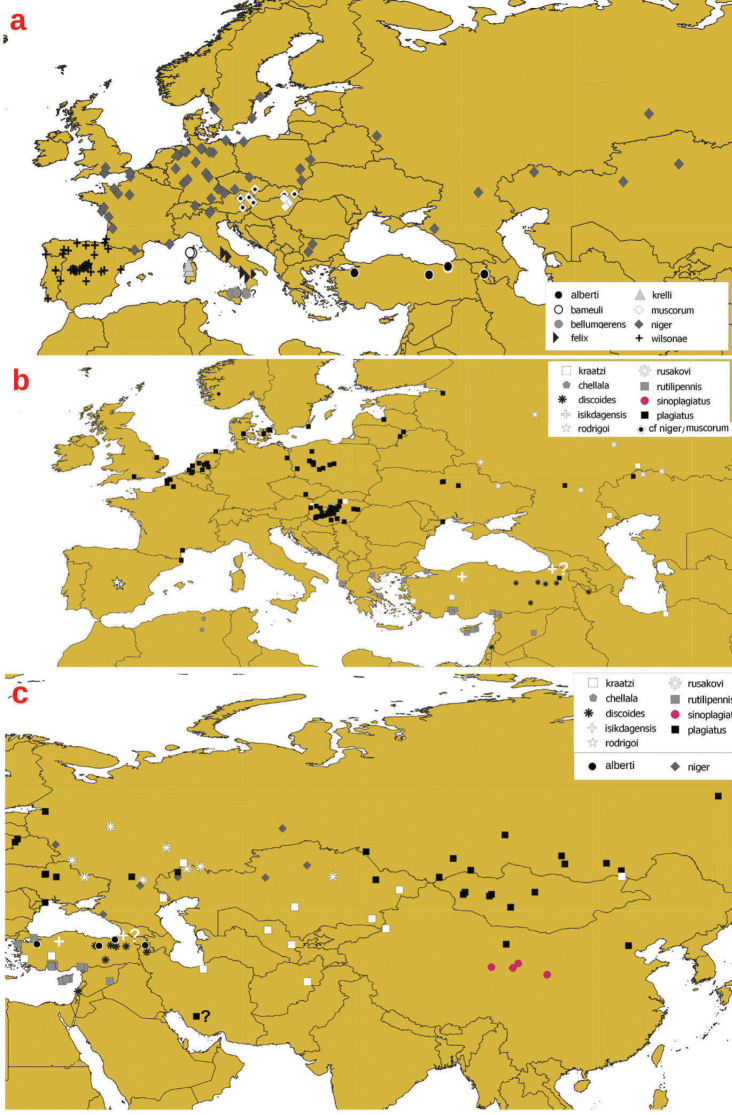
**a** distribution of *niger*-group taxa in Western and Central Europe **b** distribution of *Liothorax* species in Central and Eastern Palearctic **c** distribution of *plagiatus*-group species in Central and Eastern Palearctic. The record of *isikdagensis* with the question mark indicates a doubtful specimen from Artvin Çam, TR (Ziani Coll).

### ﻿The *plagiatus* group

Species of the *plagiatus* group are characterised by the aedeagal endophallus bearing fields of scales, hairs, or bristles, but never strong recurved teeth (Fig. 22). The basal segment of the mesotarsi is always shorter than the longer mesotibial spur (Fig. 18a–h).

#### ﻿ Aphodius (Liothorax) plagiatusplagiatus

Taxon classificationAnimaliaColeopteraScarabaeidae

(Linnaeus, 1767)

9801C793-67F4-5A07-A475-9B4C8A071C7B

Figs 2a–c , 5a–d , 7a–f , 13a, b , 17 a–c ”, 18a, 20a, b, 22h–l, 25i, j, 26a–g , 30a, b

##### Type material examined.

*Scarabaeusplagiatus* Linnaeus, 1767 (image examined on linnean-online.org/20869/#?s=0&cv=0). Designated lectotype by Krell (1995) (LSCL).

*Aphodiushungaricus* Endrődi, 1955. Type series examined (MNSB).

*Aphodiusjakutorum* Balthasar, 1938. Holotype ♂ (NMP).

##### Additional material examined.

SP: Aiguamolls de l’Empordà, Urbanización Ampuriabrava, 42.225°N, 3.089°E. spec 5445, 1♀ (MNCN).

GB: Norfolk, Hunstanton, 52.9638°N, 0.5168°W. At edge of lake after flood. 27.x.2001, R.B. Angus. 6 ♂♂, 4 ♀♀, 5 unsexed (NHMUK). Dorset, Studland Heath, 50.455°N, 1.954°E. Buried in sand by damp path. 17.v.2002, C. J. Wilson & R.B. Angus. 3 ♂♂, 1♀ (NHMUK).

LI: Litauen, Nowe Święciany, 4–20.6.1916, leg. P. Salchert, 1 unsexed; Livind, Smolwy, Sudl, Dünaburg, 16.6.1916, leg. Selchert, 1 unsexed (ABC).

HU: Hungria, Aphodius (Liothorax) plagiatus var immaculatus, 201539 (MNCN).

CZ: Dobré Pole, 48.824°N, 16.533°E, 30.iv.2011, Martinů Ivo. 1 ♂, DNA (1) (62) JFM, 4 unsexed (NHMUK);

SK: E of Chlaba, 11.vi.2014, sandy place S of railway, 47.823°N, 18.846°E, 107 m a.s.l., at light, D. Král lg. 1♂(JFMC).

RU: Pavlovsk Park, St Petersburg, 59.694°N, 30.476°E, at edge of snowmelt pool. v.1982, R.B. Angus. 1♂ (NHMUK); Yaroslavl, Berditsino, 57.454°N, 40.1108°E. leg. Yakovlev. 1♂ (ZIN); Novosibirsk oblast’, Sorochicko env. 116 m. 53.285°N, 77.887°E. Waterside litter. 9.v.2012. D.J. Mann & J. Cooter. 1♂, 2♀♀ (OUM); Tuapse, Chernomorsk oblast’, 44.098°N, 39.090°E. 1, unsexed (ZIN); Minusinsk distr. Shushenskoe, 53.324°N, 91.946°E. 1, unsexed (ZIN); Chuyskaya steppe, Kosh Agach, 49.991°N, 88.657°E. 1 ♀ (ZIN); Bunbui, Kansk neighbourhood, Yenisei government, 56.382°N, 99.025°E, 1 unsexed, black (ZIN); Transbaikal, Berezovka near Ulan Ude, 51.9320°N, 107.2400°E. 1, unsexed (JFMC).

TR: Bozkale near Kars, 40.587°N, 42.980°E (ZIN).

IN: Shiraz, Lake Maharlou, 29.450°N, 52.759°E, 1♀ (MNHP).

KZ: Yanvartsevo, right bank of the Ural River, 51.424°N, 52.239°E, leg. L. Arnoldi, 1 unsexed, black (ZIN); Koksengir S of Zhana-arka, Karaganda. 49.44°N, 79.44°E. leg. Logonova, 22.v.1958. 1 ♀ (ZIN)

MG: 40 KM Bayanbulag 45.083°N, 98.583°E. 16.vii.2005, leg. A, Mikyška. 1 ♂, elytra with red flashes (NMP); Mongolia west, 50 KM SW Uliastay. 16.vii,2005, leg. A. Mikyška, 1 ♂, elytra with red flashes (NMP); Right bank of R. Kerulen (Herlen) near the Prikhity mountains. 1 unsexed, black (ZIN)

CH: “Pekin”, Fry coll. 2♂♂, 1♀ (NHMUK).

##### Differential diagnosis.

*Aphodiusplagiatus* is recognisable by the basal segment of the mesotarsi being shorter than the longer mesotibial spur, and the heavily punctured metaventrite, especially in males. The aedeagus is distinctive, with the parameres not decurved apically and the endophallus lacking recurved teeth.

##### Redescription.

General appearance (Fig. 2a–c). Length: 3.6–4.9 mm (♂), 4.0–4.8 mm (♀); width: 1.6–2.0 mm (♂), 1.8–2.0 mm (♀). Black, head, pronotum, and scutellum glossy (pronotum sometimes less so) (Fig. 5a–d), elytra black, sometimes with a dull red streak over the middle part (Fig. 2b), interstices with very fine reticulation often giving a silky grey sheen.

Head glossy black with a diffuse dull brown area along the outer margins of the clypeus and genae, Frons with moderately strong punctation, the punctures separated by ca 1.5× their diameters, and with some sparse very fine punctures between them. Frontoclypeal suture often very fine, straight over median 2/3, then angled obliquely forward at each side, reaching the margin at the front of the genae. Occasionally the suture is more distinct. Clypeus bulging upwards medially, this bulge with fine sparse punctation which becomes coarser and rather rugose anterolaterally. Most European material has this rugose area fairly extensive and well-developed, but a male from St Petersburg (Russia) has the rugosity weaker and less extensive, and in material from the Tibetan Plateau (Qinghai and Gansu) the rugosity is further reduced (Fig. 5a–d). The genae protrude laterally at an angle of ca 45˚ in front of eyes, then curve inwards to meet the frontoclypeal suture ca 1.5× eye-diameter in front of eyes. Clypeal margin obtusely angled outward from this point, then curved inwards to the median excision. Genae laterally with a narrow raised margin, this continued across the clypeus, becoming weaker medially. Antennae and maxillary palpi black to blackish brown. Epipharynx (Fig. 7a–f) with the clithra generally only weakly sinuate either side of the tylus, but more strongly so in one Chinese specimen (Fig. 7f). Chaetopedia 2–6.

Pronotum glossy black, hemicylindrical, highly arched transversely, weakly so longitudinally. Lateral area of surface bulging outwards, almost obscuring the lateral margins (viewed from above) in basal 1/2. Surface with double punctation, the larger punctures heavy, twice the diameter of those on the head, separated from one another by 1–4× their diameter, and with very fine dot-like punctures scattered among them. The larger punctures are very sparse on the disc, denser laterally. Basal margin of pronotum varying from being entirely bordered to having the middle 1/2 unbordered (Fig. 13a, b).

Scutellum pentagonal, elongate, the sides more or less parallel in basal 1/2, then convergent apically to rounded apex. Surface glossy black with a few very fine punctures medially

Elytra black, sometimes with an oblique reddish streak over their middle parts. Interstices generally with a greyish silky sheen, contrasting with the glossy striae, but sometimes glossy black, as in the type material of *L.hungaricus* (Endrődi, 1995) (MNSB), the St Petersburg specimen already mentioned, and in material from the Tibetan Plateau. The interstices have a fine isodiametric reticulation, easily seen in most European and Mongolian material, but less so in the St Petersburg specimen, and almost invisible in the Tibetan material where the elytra appear very highly polished. Nevertheless, if the elytra are viewed with strong light and at high magnification the reticulation is always present (Fig. 17a–c). Scanning electron microscopy (Fig. 17a”, c”) confirms that this reticulation is on the elytral surface rather than being buried under the epicuticle. In most specimens the fine punctures of the interstices are very faint, though with some variation, but in the Tibetan material they are considerably stronger (Fig. 17a, a’, a”, c, c’, c”). Interstices flat, ca 8–10× wider than the striae. Striae narrow, vertical-sided, their bottoms glossy. Strial punctures in single rows, small, separated by ca 5× their diameter, and scarcely indenting the strial margins. Lateral margins of elytra gently rounded and slightly divergent over their basal 1/2, then tapered to the bluntly rounded apex.

Metaventrite median diamond-shaped plate in males flat and heavily punctate, the punctures bearing distinct yellowish setae (Fig. 20a) which may be lost, especially if an attempt is made to clean dead specimens. In females the median plate is less flat, generally depressed medially and the punctation is finer, ca 2/3 the strength of that in males, and the setae are more frequently lost, even in living material (Fig. 20b).

Legs dark brown to black, tarsi dark brown, and tibial spurs a slightly paler brown. Basal segment of mid tarsi clearly shorter than the longer tibial spur (Fig. 18a).

Aedeagus (Fig. 22a–c, h–k) ca 1.1 mm long in *A.p.plagiatus*, 1.25–1.4 mm in *A.p.sinoplagiatus* ssp. nov., the parameres widened apically and the endophallus bearing fields of small scales but no hairs or bristles. The expanded apical area of the parameres is more abruptly widened in Chinese material, giving a square-ended appearance (Fig. 22i, j, k), than in European where it is more oblique (Fig. 22h) and the holotype of *A.jakutorum* (Balthasar, 1938) (Fig. 22l) appears to match European material in this character. However, some caution is needed as this part of the parameres tends to shrivel when dried and exposed and often does not fully expand when wetted out. Note that the aedeagus of the Peking specimens shown in Fig. 22i is ca 1.1 mm long, matching *A.p.plagiatus* rather than *A.p.sinoplagiatus* ssp. nov.

Spermatheca (Fig. 26a–g) ca 0.36 mm long in *A.p.plagiatus*, but clearly longer in *A.p.sinoplagiatus* ssp. nov., length ca 0.42 mm.

##### Remarks.

*Aphodiusplagiatus* is the most widely distributed of all the Palaearctic *Liothorax*, with its range extending from England in the west to east Siberia (Yakutia) and China (Qinghai, Gansu, and the Beijing area) in the east (Fig. 29b, c). The southern limits of its range are unclear. There are no confirmed records from the Iberian Peninsula and the Catalogue of Palaearctic Coleoptera gives no records from Italy. Records from the Balkans require confirmation because of confusion with *A.rutilipennis*. All material from Syria examined in this study has been determined to be *L.discoides* and not *L.plagiatus* as reported by Baraud (1992). The only (doubtful) record of *L.plagiatus* from the Middle East is a female from Iran (Shiraz) found in the collections of the Paris Museum by one of the authors (JFM) which was collected during the 1965 Mission Franco-Iranienne and was identified by Baraud as *L.niger*. This specimen was not mentioned by him in his Coléoptères Scarabaeoidea d´Europe (1992) nor, to our knowledge, by any other author, and, apart from *A.kraatzi* from Ashooradeh Island is the only specimen of *Liothorax* known for Iran. This specimen was examined in 2004 and tentatively identified as a form of *wilsonae* or *plagiatus* based on a strongly punctured metaventrite, shortened tarsi, and a surface finish notably different from *niger*, but due to its divergent morphology was not included in the type series. In light of the work done since, we suspect that it is a member of the *plagiatus* group, but requiring additional material, in particular males. As such this record has been labelled on the distribution maps (Fig. 29c) with a black question mark and a black square.

One of the peculiarities of *A.plagiatus* is the existence of two distinct colour forms, one plain black and the other, exemplified by the lectotype presumably from Sweden, with a red longitudinal-oblique flash over the central area of each elytron. One of us (RBA) has noted the red-streaked form in England, Ukraine (Kiev and Odessa), and Mongolia. The red form appears to be absent from the Czech Republic and Hungary as well as from Russia. As far as is known, this red-streaked form does not occur in *A.plagiatussinoplagiatus*.

*Aphodiusplagiatus* is normally found at the roots of vegetation in damp places and is often found washed up among strandline detritus at the edge of pools (Fig. 28a, c). It is generally associated with salinity, as in the Qinghai pool shown in Fig. 28c, but there did not appear to be any trace of salinity in Pavlovsk Park near St Petersburg where RBA collected one specimen at the edge of a snowmelt pool in April 1982 (Fig. 28a).

#### ﻿ Aphodius (Liothorax) plagiatussinoplagiatus subsp. nov.

Taxon classificationAnimaliaColeopteraScarabaeidae

A3AAB4F7-DFCF-5000-804C-A4F8B4FB44E5

https://zoobank.org/4ADB8359-223B-4AFA-B942-510A6EF9C63C

**Figure 30.**
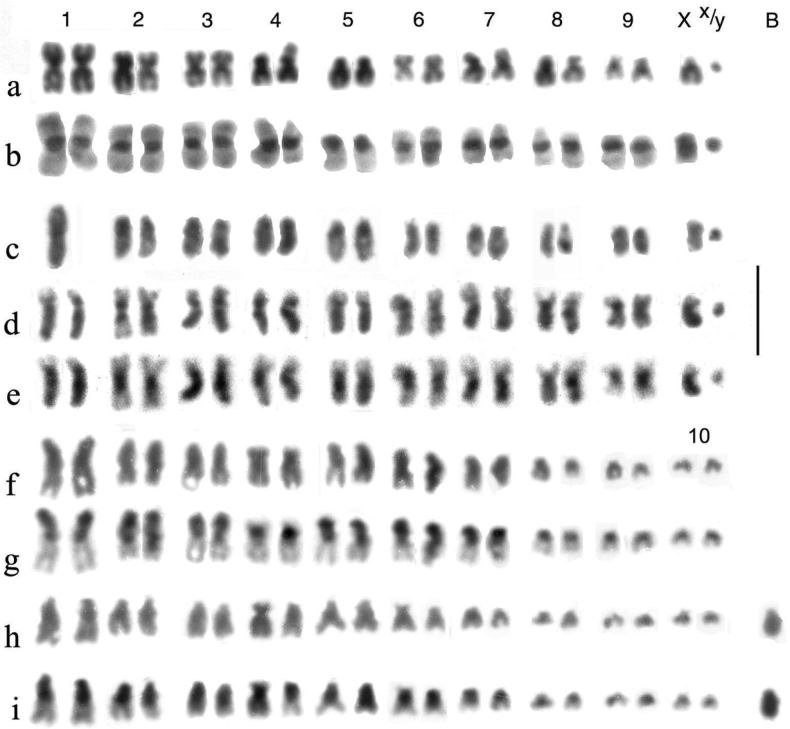
Mitotic chromosomes, arranged as karyotypes **a**, **b***A.plagiatusplagiatus*, ♂, GB, Dorset, Studland **a** plain (Giemsa stained) **b** the same nucleus C-banded **c***A.plagiatussinoplagiatus*, ♂, CH, Qinghai, Gangca, plain **d**, **e***A.rutilipennis*, ♂, CY, Zakaki Marshes **d** plain **e** C-banded **f**–**h***A.kraatzi* ♀, SK **f**, **g** nucleus without B-chromosomes **f** plain **g** C-banded **h**, **i** nucleus with 1 B-chromosome **h** plain **i** C-banded. Scale bar: 5 μm.

Figs 22j, k , 26f, g , 30c

##### Type material examined.

***Holotype*** ♀ Amdo/1884/Przevalsky (printed label), Zaidam/ Burch Budde/1-11-000 // *Aphodius*/*plagiatus*/var. (handwritten) (ZIN). ***Paratypes***: 1 ♂, data as holotype (originally on the same pin) (ZIN); 1♂ China Qinghai/N. Qinghai Hu/Gangca. Roadside/pool Printed label and a further label with the same data in Chinese. 37°18'N, 100°11'E/3370 m. 5.vi.2013,/R.B. Angus, F.L. Jia & Y. Zhang.// *A.plagiatus* (L.)/ Chromosome prep./6.vi2013./R.B. Angus //JFM 130815017 (printed labels) (NHMUK); 1♀ Cina-Gansu/Dingxi/7.8 – 12.8 1996/E. Kučera leg.//Ex coll. David Král/(NMP).

##### Differential diagnosis.

Aphodius (L.) plagiatus sinoplagiatus differs from A. (L.) p. plagiatus by its very glossy varnished appearance, the slightly larger aedeagus (length ca 1.25–1.4 mm) with abruptly widened paramere apices (Fig. 22j, k), and above all by the strikingly larger spermatheca, length ca 0.42 mm (Fig. 26f, g), as against ca 0.36 mm in A. (L.) p. plagiatus (Fig. 26a–e).

##### Description.

Length: 4.1–4.4 mm (♂), 4.1–4.6 mm (♀), width 1.8–2.0 mm (♂), 1.8–2.0 mm. Head: glossy black, sparsely double punctate, clypeus bulging upwards, frontoclypeal suture without tubercles, straight over middle ¾, effaced laterally. Outer angles of anterior emargination rounded.

Pronotum: highly arched transversely, lateral margins not visible from above medially. Surface sparsely double punctate, the larger punctures slightly stronger than on head, the Gansu male more heavily punctate. Basal margin largely unbordered, the raised margin extending ca ¼ of the way in from the hind angles.

Elytra black, highly polished (varnished), as scutellum. Metaventrite (♂ and ♀) as in the nominal subspecies. Legs mid- to dark brown, longer mesotibial spur longer than basal segment of mesotarsus.

Aedeagus (Fig. 22j, k) length 1.25–1.4 mm, outer apical membranous part abruptly widened.

Spermatheca (Fig. 26f, g) length ca 0.42 mm.

##### Etymology.

*sinoplagiatus* is an adjective indicating its Chinese distribution.

##### Remark.

The aedeagus of 2 ♂♂ from near Beijing (Fig. 22i) resembles that of *L.p.sinoplagiatus* in its widened paramere apices, but is smaller, length ca 1.1 mm.

#### ﻿ Aphodius (Liothorax) rodrigoi sp. nov.

Taxon classificationAnimaliaColeopteraScarabaeidae

B1B5BBB2-3CEA-5A6D-A84D-B2710D8655A0

https://zoobank.org/ABCB9508-F963-4C3D-ACDA-CEB4642AC551

Figs 2d , 7g , 11f , 20j , 22 m, m ’, m”, 25k

##### Type material examined.

***Holotype*** ♂: Spain: Madrid Aranjuez F. Morüder // Aphodiusplagiatus det C.M. Veiga 1990 (MNCN). ***Paratypes***: 4 ex ♀ Aranjuez F. Morüder // DA 251 F.J.Cabrero // DG 251 F.J.Cabrero // AP Bucal 206 F.J.Cabrero // Aphodiusplagiatus det C.M.Veiga 1990; 1 ex; ♂ Quero Prov Toledo Lauffer // DG 178 F.J.Cabrero // DA 249F.J.Cabrero // Aphodiusplagiatus det C.M.Veiga 1990; 1 ex; ♀ Quero v.1908 Molina // AP Bucal 56 F.J.Cabrero // DG 174 F.J.Cabrero // Aphodiusplagiatus det C.M.Veiga 1990; 1ex; ♂ Villacañas (TOLEDO) C. Bolívar // Hoyer C.M.Veiga // AP Bucal 205 F.J.Cabrero // DA 250 F.J.Cabrero // Aphodiusplagiatus det C.M.Veiga 1990 1 ex. (MNCN).

##### Differential diagnosis.

Aphodius (L.) rodrigoi resembles a small highly-polished somewhat rounded A. (L.) plagiatus. The metaventrite is slightly less strongly punctate and with the median diamond-shaped area depressed to mid-line. Aedeagus: (Fig. 22m, m’, m”) general size and shape of parameres similar to *A.plagiatus*, not turned downwards apically, but with the following differences: apices of parameres less produced laterally, parameres narrower and more converging towards apex, in lateral view parameres gradually curved towards apex and narrowest towards apex compared to *L.plagiatus* which is narrowest 1/3^rd^ towards apex.

The apical segment of the maxillary palpi is slightly longer (10%) than the 2^nd^ segment: terminal segment slightly longer (10%) than 2^nd^ segment (Fig. 11f), as against 15–20% longer in *A.plagiatus* (Fig. 11d, e).

##### Description.

General appearance (Fig. 2d). Length: 3.5–4.0 mm (♂) (HT 3.75 mm), 4.0–4.23 mm (♀); width: 1.4–1.6 mm (♂ and ♀).

Glossy black throughout except clypeal and elytral apical edges which are fuscous red. Legs and maxillary palps reddish yellow to maroon, antennae tan except for last three segments which are darker and clothed in off-white setae.

Head: frons strongly convex, clypeus flat to slightly concave. Frontoclypeal suture weak, widely interrupted in the middle. Clypeal edge truncated to slightly emarginate, impressed medially; side angles rounded with edge widely elevated. Sides of head almost straight and continuously merging with genae. Genae produced beyond eyes, rounded, strongly bordered and with some short yellowish setae.

Surface shiny with residual reticulation and strongly punctured. Punctation double, the larger punctures regularly dispersed on the clypeal sides and frons, absent from genae and anterior clypeus. Larger punctures 2–3× diameter of the smaller ones and strongly impressed, particularly on the sides where the surface almost appears wrinkled and closely spaced (1–1.5× their diameter). Anterior edge of clypeus very finely and regularly punctured, completely devoid of larger punctures.

Maxillary palp: terminal segment slightly longer (10%) than 2^nd^ segment (Fig. 11f) (in *A.plagiatus* the terminal segment is longer, 15–20% longer than the 2^nd^ segment) (Fig. 11d, e). Galeal patch armed with five or six strong galeal chaetae (7–9 in *A.plagiatus*). Galea smaller, similar in dimensions to the Tibetan ssp. of *A.plagiatus* but not to European populations in which it is larger.

Epipharynx (Fig. 7g) corypha small and only slightly produced (somewhat similar to the Tibetan populations of *A.plagiatus*), with the chaetae (celtes) much reduced in size. Fenestrae on zygum (angustofenestrae) and chaetae (heli) less numerous than in *A.plagiatus* and limited to the edge against pedia. Chaetopaedia as in *A.plagiatus*. Chaetopariae noticeably stronger than in *A.plagiatus*.

Pronotum subquadrate, sides slightly rounded, subparallel, and widest towards base. Regularly convex longitudinally and transversely. Base completely and distinctly bordered, border strong but fine. Anterior edge not bordered at all. Lateral margins visible dorsally in the apical 1/3 only. Sides strongly bordered throughout, anteriorly going around corner up to the middle of the eye. Lateral margins with short yellowish hairs barely visible dorsally in apical 1/2.

Surface of pronotum black and shiny, not at all alutaceous with only some residual superficial shagreen. Punctation double, larger punctures regularly distributed throughout but densest on the sides and anterolateral corners. Large punctures flat bottomed and umbilicated. Diameter 4–5× that of the smaller ones and spaced 1–3× their diameter.

Scutellum elongate, sides rounded, convergent throughout (only slightly pentagonal), length 1/11 elytral length. Glossy black, unpunctured, basally impressed, disk convex.

Elytra black, intervals convex, surface strongly glossy and slightly alutaceous under high magnification. Intervals with double row of very fine and faint punctures. Striae fine, 1/8× width of intervals, with sides crisp and right-angled. Regularly punctured, punctures wider than the striae and separated by 2× diameter (first 2 striae) or 3–4× (remainder). Elytral epipleura strong, gradually convergent towards apex, and at humeri forming a small but distinct tooth.

Underside black with yellowish pubescence on the abdomen.

Metaventrite (Fig. 20j): median diamond-shaped area rather strongly punctate, glabrous, its mid-line distinctly impressed. Surface regularly heavily punctate, the punctures with one end somewhat pointed, shiny, and not alutaceous. Sexual dimorphism subtle, female flatter than male.

Legs reddish to dark brown, tarsi and tibial spurs red to tan, rather long and slender. Protarsal spur regularly acuminate and curved in both sexes, reaching to apex of second tarsal segment. Metatarsal segments short. Basotarsomere slightly shorter than upper metatibial spine as long as segments 2 + 3. Fimbrial setae short and of unequal length. Longer spur of mid tibiae as long as first two tarsal segments.

Aedeagus: (Fig. 22 m, m’, m”) general size and shape of parameres similar to *A.plagiatus*, not turned downwards apically, but with the following differences: apices of parameres less produced laterally, parameres narrower and more convergent towards apex, in lateral view parameres gradually curved towards apex and narrowest nearer apex (ca ¼ of the paramere length as against ca 1/3 in *L.plagiatus*).

Endophallus typical of other members of the *A.plagiatus* species group, with two patches of elongate triangular scales or bristles. Those in basal patch longer and more elongate than any member of the *plagiatus* group (19–26µm in *A.rodrigoi* vs 14 –21µm), similar to those of *A.wilsonae* (22–32µm), those in the apical patch like *A.plagiatus*.

##### Etymology.

The name is derived from the surname of the name Joaquín Rodrigo Vidre, a Spanish composer whose most famous work, “Concierto de Aranjuez”, refers to the geographical area from which the species hails.

##### Remarks.

All the known specimens are from the south of Madrid (Aranjuez) and the adjacent areas of Toledo (Fig. 29b) The localities are associated with temporary endorheic saline lagoons and lakes (Fig. 28b) One of the authors (JFM) made repeated visits to several of the localities during late winter over several years (January and April of 2015–2017), but no specimens were found. Although it is possible that the species was missed due to its ecology or time of emergence, it is also possible that the species is very rare or has been extirpated from much of its former distribution. The known localities are part of an extensive system of aquifer-fed saline lagoons encompassing much of La Mancha and collectively known as “La Mancha Húmeda” (Wet La Mancha). Many of these lagoons are degraded or lost to agriculture but almost 1/2, including several visited for this study, are protected and in apparently good ecological condition (Florín-Beltrán 2001). Hence, it would be hasty to declare this interesting Iberian endemic extinct without carrying out extensive sampling across the area and in particular within the protected lagoons which might still host it.

#### ﻿ Aphodius (Liothorax) discoides

Taxon classificationAnimaliaColeopteraScarabaeidae

A. Schmidt, 1916, stat. rest.

32BC2D71-A956-58A5-8915-FC2A717EC635

Figs 2j, k , 5e–h , 7h , 14a , 17g–i , 18e , 20k, l , 22d–g, e ’, n’, o’, 25n, 26h, i

Aphodius (Nialus) plagiatus var. discus Reitter 1892: 204.
Aphodius (Nialus) plagiatus var. discoides Schmidt, 1916: 96 (new name for AphodiusdiscusReitter 1892, not Aphodiusdiscus Wiedemann, 1823).
Aphodius (Nialus) bytinskisalzi Petrovitz, 1971: 219.

##### Note.

Reitter described Aphodiusplagiatus “ var. discus m” as having the elytra yellow with only the wide margins black, and gave the locality “Araxes, Syria” (now Aras). Schmidt (1916) proposed the replacement name *A.discoides* as Reitter’s *Aphodiusdiscus* is a junior homonym of *Aphodiusdiscus* Wiedemann, 1823. Dellacasa et al. (2007) dismiss these names as unavailable as they refer to colour variants, but this is incorrect, as it would be the case only if the name was “infrasubspecific”. For names published before 1961, the International Code of Zoological Nomenclature (1999, 4^th^ edition) states that a name “is subspecific if first published before 1961 and the author expressly used one of the terms “variety” or “form” (including the use of the terms “var.”, “forma”, “v” and “f”), unless the author also expressly gave it infrasubspecific rank, or the content of the work unambiguously reveals that the name was published for an infrasubspecific entity”. Reitter clearly stated that his name referred to a variety from a known locality, meaning that the taxon concerned was subspecific rather than infra-subspecific. This name is therefore available and, in the form of Schmidt’s replacement name (*discoides*) is the oldest known name for this species. We have been unable to locate any type material of Reitter’s *A.discus* and therefore here designate a neotype. The specimen chosen is the holotype of *A.bytinskisalzi*, a male whose aedeagus has the endophallus extruded, from the same general area as Reitter’s material.

##### Type material examined.

***Holotype***. *Aphodiusbytinskisalzi* ♂ Israel, Kuneitra. 6.iv.1968. Leg Bytinski-Salz Tel-Aviv. This specimen is here designated as the neotype of *L.discoides* Schmidt. 33.12657°N, 35.8586°E (IL-SMNH.)

##### Additional material examined.

TR:1 ♂, “Turkei leg Schönmann et Schilhammer” “Prov. Diyarbakir Karacadag 28.5.1987. “Aphodius (Liothorax) plagiatus det Pittino 1989” (NMW); 1 ♂, Turkey Karacadag In coll. D. Král (NMP); 1♀, Turkey, 24.vi.1993, Kizildag env. Klíma leg. In coll. D. Král (NMP); 1 unsexed, Turkey, 1.v.1998, Erzincan 8 km Askale. Leg Sama (SZ); 1 ♀, Turkey, 17.v.2011, Muş 1400 m Buģlan geçidi G. Sama leg. (SZ); 1 black ♂, Turkey or. Karakurt env. In coll. D. Kràl (NMP).

##### Differential diagnosis.

This species agrees with *A.plagiatus* in the general form of the aedeagus, and in particular the absence of any bristles on the endophallus, which distinguishes it from all other known species. The lightly punctate central area of the metaventrite gives a clear separation from *A.plagiatus*. Most of the studied material has the elytra largely yellow, unique among the known Palaearctic *Liothorax*, but recognition of black specimens requires study of the aedeagus.

##### Redescription.

General appearance (Fig. 2j, k). Length: 3.8–4.8 mm (♂), 3.4–4.5 mm (♀), width 1.65–2.1 mm (♂), 1.5–2.0 mm (♀).

Head black, finely and sparsely double punctate, with small punctures and minute dots, the punctures separated by ca 2× the diameter of the larger punctures, punctation slightly rugose towards the anterior margin, especially either side of the median emargination of the clypeus. Sides of clypeal emargination rounded. Frontal suture almost invisible, but median transverse portion present, with a small vague elevation medially, just behind median bulge of clypeus, in males, and weakly raised at ends, just mediad of inner margins of eyes, where it would be expected to angle forwards to anterior margin of genae. However, this part of the suture is effaced. Centre of clypeus raised. Maxillary palpi blackish brown, antennae mid brown, clubs darker. Epipharynx (Fig. 7h) with the anterior margin of the clithra only weakly emarginated either side of the tylus, and the apophobae rather stout and the prophobae restricted to areas close to the edges of the basal 1/2 of the mesoepitorma.

Pronotum black with anterior and lateral margins vaguely browned. Hemicylindrical, strongly arched transversely, scarcely at all longitudinally. Punctation double, sparse, stronger than on head, punctures separated by at least 2× the diameter of the larger punctures. The larger punctures more developed posteriorly and laterally, almost absent from the anterior-median 1/3. Pronotal surface with a fine weak isodiametric reticulation (viewed at ×80). Sides of pronotum almost straight, parallel, incurved anteriorly. Pronotal surface bulges out laterally in basal 1/4, but the basal 1/4 of the lateral margins is still just visible from above. Head and pronotum (Fig. 5e–h): basal margin of pronotum unbordered in middle 1/3 (Fig. 14a).

Scutellum elongate, glossy dark brown, pentagonal, sides parallel in basal 1/2, then convergent to bluntly pointed tip.

Central area of elytra typically dull yellowish brown, semi-transparent so that the folded wings are visible, suggesting a vague darker pattern over this part of the elytra. Sutural interstice darker, as are the outer interstices (interstice 7 to the edge), with the darkening of interstice 7 sometimes confined to the outer edge just behind the humeral callus, then expanding over the entire interstice to the apex. Interstice 6 may be darkened in apical 1/2, and the apical 1/3 of the elytra is darkened throughout. Striae narrow, shallow, strial punctures scarcely encroaching on the strial edges. Interstices flat, sparsely and very finely punctate, ground with a fine isodiametric reticulation giving a silky sheen, though the strength of the reticulation varies (Fig. 17g–i). In the black male listed above the elytra and scutellum are entirely black. This specimen agrees with *A.discus* in the shape and sculpture of the head and pronotum and in the endophallus with scales only, lacking any hairs or bristles.

Median diamond-shaped plate of metaventrite (Fig. 20k, l) weakly depressed to mid-line, finely punctate, the surface with fine isodiametric reticulation.

Legs: dark brown, tarsi a little paler. Longer spur of mid tibiae clearly longer than basal segment of tarsus, extending to ca the middle of segment 2 (Fig. 18e).

Aedeagus (Fig. 22d–g) similar to that of *A.plagiatus*, but smaller, length ca 0.85 mm. Endophallus with short fine scales and with a field of densely packed columnar bristles at its apex (Fig. 22e’). The scales of the basal section of the aedeagus are the same size and shape in the black specimen as in the ones with paler elytra (Fig. 22n’, o’).

##### Remarks.

*Aphodiusdiscoides* appears to have a rather restricted distribution in Anatolia, Syria and Israel (Fig. 29b). It appears to be a rather rare species. We have no habitat information.

#### ﻿ Aphodius (Liothorax) rutilipennis

Taxon classificationAnimaliaColeopteraScarabaeidae

Baudi di Selve, 1870, stat. rest.

F7A41880-9EFA-50C9-98DB-BED70B4A40A9

Figs 2e–g , 5i–k , 7j , 13c, d , 18c , 20c, d , 22q, q ’, r, v, w, 25m, 26j, k, 30d, e

Aphodius (Liothorax) plagiatus var rutilipennis Baudi di Selve,1870: 64.
Aphodius ressli Petrovitz, 1962: 126
Aphodius cypricola Balthasar, 1971: 57.
Aphodius isikdagensis Balthasar 1952, sensu Dellacasa et al. 2001b: 6 (misidentification).

##### Note.

Baudi di Selve (1870) described a variety of *A.plagiatus* in which the elytra were brownish bronze (“brunneo-subaeneis”) and said that this variety was less rare (on Cyprus) than the black form. He added that, among other things, the mesosternum (sic) was more delicately and less densely punctate than in European *A.plagiatus*. Dellacasa et al. (2007) stated that the name *A.rutilipennis* Baudi di Selve was unavailable as it was simply a colour variant, but that would only be the case if the name was “infrasubspecific”. For names published before 1961, the International Code of Zoological Nomenclature (1999, 4^th^ edition), states that a name “is subspecific if first published before 1961 and the author expressly used one of the terms “variety” or “form” (including the use of the terms “var.”, “forma”, “v” and “f”), unless the author also expressly gave it infrasubspecific rank, or the content of the work unambiguously reveals that the name was published for an infrasubspecific entity”. Baudi makes no reference to infrasubspecific status, nor does his description imply that it was such. This name is therefore available and is the oldest known name for this species.

##### Type material examined.

Aphodiusplagiatusvarrutilipennis Baudi di Selve,1870. Type material in the Museo Civico di Storia Naturale Giacomo Doria, Genova (MCSNGDG) – not examined (not available for study).

*Aphodiusressli* Petrovitz, 1962. Holotype ♀ (MNHG);

*Aphodiuscypricola* Balthasar, 1971. Holotype ♂ (NMP).

##### Additional material examined.

GR: Corfu, 39.6°N, 19.8°E. Reitter, Nevinson coll. 1♂ (NHMUK). Evia, Chalcis, 36.463°N, 26.635°E, 1932, leg. J. Fodor. 1 unsexed (MNHB). Macedonia, Langassa Göll. 40.464°N, 32.147°E. 1♀ (MNHB).

AL: Albania mer. Butrint, 39.754°N, 20.021°E. Smetana, 1958, l♀ (NMP).

TR: Besika (Beşik) Bay, 40.274°N, 26.459°E. 2 ♂♂, 5 unsexed. Ex C.G. Champion (NHMUK). Turkey mer. Devecusagi, Yumurtalik env., 36.884°N, 36.039°E. 14.iv.1992. leg. O. Hovorka. 1 unsexed (NMP) prov. Antalya, Manavgat. 36.763°N, 31.437°E. leg. A. Bellmann 13.iii.2000. 1♂ (ABC). Antalya, Manavgat. 36.763°N, 31.437°E. leg. A. Bellmann 13.iii.2000. 1♂ (ABC). Antalya, 5Km nördi, Sagirin, Köprülu kanyon, 19.03.2002, leg. A. Bellmann, 1 unsexed (ABC); same data, 22.03.2002, 1 unsexed (ABC); Antalya, Akseki, 1300 m a.s.l., 19March2002, leg. Bellmann 1 unsexed (ABC); Alanya, Konakli; 13.03.2000 leg. A.Bellmann, 1 unsexed (ABC). Istanbul Halkali 28.vii.1968 Cl. Besuchet, 1 unsexed (MHNG).

CY: Akrotiri, 34.634°N, 32.991°E. G. Mavromoustakis 2♂♂ (NHMUK); Limassol distr. Zakaki marshes, puddle in lorry park at edge of reedbed, 3.iv.2005. R.B. Angus. 3 ♂♂, chromosome prep. 4: 7.iv.2005 R.B Angus, DNA extracts 110 & 91, J.F. Maté 2013, 1♀, chromosome prep. 5: 7.v.2005 (NHMUK); “Insel Cypern” ex coll, Fodor. 2♂♂, 1♀ (MNHB); Zypern, Sotira (Salzee), 6.4.2010, W. Ziegler leg., 1 unsexed (ABC).

##### Differential diagnosis.

A small black *A.plagiatus* group species, occasionally with dull reddish elytra, immediately distinguished from *A.plagiatus* by its clearly more lightly sculptured metaventrite and the bristles on the endophallus. Black specimens are distinguished from *A.chellala* sp. nov. by the narrower gap in the basal border of the pronotum.

##### Redescription.

General appearance (Fig. 2e–g). Head and pronotum (Fig. 5i–k). Length: 3.1–4.1 mm (♂), 3.9–4.2 mm (♀); width: 1.4–1.8 mm (♂), 1.7–1.9 mm (♀).

Head black, anterolateral margins of clypeus and genae with a vague dull yellowish brown strip, and actual margins narrowly raised. Frontoclypeal suture more or less straight over median 1/3 but middle part effaced, laterally angled to anterior edge of genae, but this part scarcely traceable. Frons with moderate double punctation which is effaced on the middle 1/4. Disc of clypeus bulges upwards, this bulge with only very fine sparse dot-like punctation, surface away from the bulge with moderate double punctation and the surface becoming weakly wrinkled towards the anterior and lateral margins. Front of clypeus weakly excised over middle 1/3, recurved either side of this excision. Antennae and maxillary palpi dull yellowish brown antennal clubs and apical segments of palpi darker. Epipharynx (Fig. 7j) with the anterior margin if the clithra excised either side of the median tylus, and the prophobae and apophobae quite extensively developed and with 3 or 4 chaetopedia each side.

Pronotum hemicylindrical, highly arched transversely, weakly so longitudinally. Lateral margins bordered but basal 1/2 not visible from above because of the outwardly bulging lateral parts of the pronotal surface in this area. Posterior margin of the pronotum usually with an almost continuous border, this with a small median interruption whose width is approximately the same as the diameter of the larger pronotal punctures (Fig. 13c). In the holotype of *A.cypricola* and a female from Greek Macedonia (Langasa-Göl, now Lagkadas near Thessaloniki) the median gap in the basal border of the pronotum is rather wider (Fig. 13d), but the aedeagal characters are the same as in other material. Pronotal surface with moderate double punctation, the larger punctures separated by 1–2× their diameter, becoming sparser medially. Smaller punctures dot-like and all punctures absent from a median longitudinal strip.

Scutellum elongate, pentagonal, glossy reddish brown with a few punctures medio basally.

Elytra maroon-bronze or black, interstices with a silky sheen due to the fine reticulation on their surfaces. Striae narrow, shallow and glossy, with punctures separated by ca 2× their diameter and indenting the strial margins. Interstices ca 6× as wide as the striae, flat and with a very fine reticulation and scattered very fine punctures separated from one another by ca 1/3 of the width of the interstices. Lateral margins of elytra slightly rounded to widest point just behind the middle, then rounded to the blunt apex.

Metaventrite: (Fig. 20c, d) grey-bronze with maroon reflections, median diamond-shaped plate depressed medially. Surface reticulate, the reticulation effaced in the middle of the median depression. Punctation fine and sparse, becoming stronger over the outer part of the median depression, but absent from its centre.

Legs: mid brown, basal segment of mid tarsi clearly shorter than the longer tibial spur (Fig. 18c).

Aedeagus (Fig. 22q, q’ r, v, w) small, length ca 0.7 mm with the endophallus bearing a dense field of bristles dorsally and the apices of the parameres membranous, expanded and obliquely truncate.

##### Remarks.

*Aphodiusrutilipennis* is distributed in mainly lowland coastal areas of southeast Europe and Asiatic Turkey (Anatolia) from Corfu and southern Albania (Butrint) in the west, via Evia and Macedonia (Greece), Besika Bay (Turkey), Cyprus (Limassol and Larnaca districts) to Hatay (Iskenderun) (Turkey: the type locality of *L.ressli*) in the east (Fig. 29b, c) On Cyprus it appears to be mainly winter-active. RBA collected it at the edge of a muddy pool in the Zakaki marshes (Limassol) and C. Makris (Limassol, Cyprus) collected it from under a discarded piece of carpet lying on the mud in the same area (pers. comm. to RBA, April 1985). This whole area is slightly saline.

#### ﻿ Aphodius (Liothorax) chellala sp. nov.

Taxon classificationAnimaliaColeopteraScarabaeidae

3F6E65BA-E811-5765-BEA7-804ADA8107F4

https://zoobank.org/D293FE92-32FA-4B73-8FB6-15CE48B7D55C

Figs 2h , 5l , 7k , 13e , 17e , 18d , 20e, f , 22s–s ”’, t, u, 25l, 26l

##### Type material examined.

***Holotype*** ♂: Chellala, Algeria (33.074°N, 0.123°W) De Vauloger. Nevinson coll. 1918 – 14 (NHMUK). ***Paratypes***: 8 ♂♂, 14 ♀♀, 1 unsexed (lacking head), data as holotype. 1 ♂ and 2 ♀♀ have the labels “Prov. D’Alger. Chellala 1895 de Vauloger”, these presumably the basis of the English labels on the other specimens. 9 ♂♂, 3 ♀♀, Taguin (near Zmalet El Emir Abdelkader, 35.221°N, 1.402°W), Algeria, De Vauloger. Nevinson coll. 1918 –14. NHMUK. As with the Chellala material, 2 ♂♂ have the French label “Prov. D’Alger Taguin 1895 de Vauloger” (NHMUK).

##### Differential diagnosis.

Closely resembling dark specimens of *A.rutilipennis* but basal margin of pronotum (Fig. 13e) more broadly interrupted than in *A.rutilipennis* (Fig. 13c, d). Endophallus of aedeagus, when extruded, with the scale-patches (Fig. 22q’, s”’) clearly different from the dense bristle-field of *A.rutilipennis* (Fig. 22q, q’).

##### Description.

General appearance (Fig. 2h). Length 3.6–4.5 mm (♂), 3.7–4.5 mm (♀), width 1.5–1.8 mm (♂), 1.6–1.9 mm (♀). Black, antennae, maxillary palpi and legs dark brown. Head: glossy black with anterior from in front of the genae dull brown, this colour merging into the general black ground colour. Anterior margin of clypeus emarginate medially, sides of the emargination rounded. Surface glossy with no trace of reticulation but with sparse double punctation, the larger punctures scarce medially. Disc of clypeus raised as a hump. Frontoclypeal suture fine, without tubercles, complete, angled forward just mediad of the inner margin of the eyes, running to anterior end of genae. Genae protruding in front of eyes, rounded, no angle between anterior end of genae and clypeus. Head and pronotum (Fig. 5l): epipharynx (Fig. 7k) similar to that of *A.rutilipennis*, but, at least in the specimen figured, more heavily sclerotised medially. Pronotum: hemicylindrical, highly arched transversely and weakly so longitudinally in mid-line. Sides of pronotum weakly curved, somewhat convergent anteriorly. Lateral margins in basal 1/4 not visible from above, concealed by lateral bulging of pronotal surface. Surface glossy, with no trace of reticulation but with sparse large punctures interspersed with much finer ones, these ca 1/5 of the diameter of the larger ones. In some specimens the punctation is a little stronger and closer. Anterior margin of pronotum not bordered, lateral margins bordered from just mediad of anterior angles to base, this border extending (as a very fine impressed line) over the lateral 1/3 of the hind margin. Median 1/3 of hind margin without any trace of an impressed line (Fig. 13e). Scutellum pentagonal, sides parallel in basal 1/2, the convergent to blunt apical point. Surface glossy black, impunctate. Elytra black, not quite parallel-sided, widest just behind middle then sides convergent to bluntly rounded apex. Interstices wide and almost flat, ca 5× the width of the striae, silky black due to fine indistinct reticulation, and with sparse fine punctures, these separated by ca 8× their diameters. Elytral sculpture: Fig. 17e. Striae narrow, vertical-sided, glossy, strial punctures encroaching on their margins. The striae stop short of the elytral apex on the inner 1/2 of the elytra, leaving a smooth glossy apical field. Metaventrite (Fig. 20e, f) fairly finely punctate, median furrow of central diamond-shaped plate flattish. No consistent sexual dimorphism. Legs dull mid- to dark brown. Long spur of mid tibiae clearly longer than the basal segment of the tarsi (Fig. 18d). Hind tibiae with apical spines fairly long and unequal on dorsal/external edge, shorter and of even length along ventral/internal edge.

Aedeagus (Fig. 22s–s”’, t, u) small, length ca 0.6 mm. Apex of parameres membranous, obliquely truncate, often distorted in dried specimens, even when they are soaked again. Endophallus with scales, some of which are elongate, when not everted indistinguishable from that of *A.rutilipennis* (Fig. 22v, x). However, the everted endophallus shows a clearly different arrangement of scale-patches (Fig. 22q’, s”’). The two specimens with the endophallus everted were the only ones where it had become extruded by partial decomposition of the specimen. These endophalli were softened in dilute potassium hydroxide, transferred to alcohol and then critical-point dried. Unfortunately the better of the two (Fig. 22s–s”’) disintegrated when removal from the SEM stub was attempted. For this reason, the specimen with the less good endophallus is chosen as the holotype.

##### Etymology.

The specific name *chellala* is a feminine noun, the name of the type locality.

##### Remarks.

So far known only from North Africa (Fig. 29b). We have no information about the habitat.

#### ﻿ Aphodius (Liothorax) kraatzi

Taxon classificationAnimaliaColeopteraScarabaeidae

Harold, 1868

FE605F66-CB5B-5078-A68A-185AA9D27B99

Figs 3a, b , 5o, p , 8a, b , 14b , 17j , 18f, g , 20g–i , 22y, z , 25h , 26o–s , 30f–h

Aphodius kraatzi Harold, 1868: 84.

##### Type material examined.

(MNHP), seen but not studied in detail by DK.

##### Additional material examined.

SK: S. Slovakia, E. of Chlaba, sandy place S of the railway. 47.842°N, 18.844°E. 107 m a.s.l.. 11.vi.2014. leg. D. Král. 1♂, 5♀♀ (NHMUK). Martovce. 47.858°N, 18.127°E. 2 ♀♀ (NHMUK). Velké Kosíhy. 47.734°N, 18.127°E. 1♀ (NHMUK). Virt. 47.742°N, 18.324°E. 31.vii – 2.viii.2012. Martinů Ivo leg. 2, unsexed (NMP).

GR: Lesbos, Petra. 39.399°N, 26.181°E. 2, unsexed (NMW).

RU: Astrakhan obl. Dosang. 46.904°N, 47.916°E. Cattle dung. A. Frolov & L. Akhmetova. 2♀♀, 4 unsexed, 23.v.2007, 1 ♀, 1–3.v.2004 (NHML). Novosibirsk obl. Karasuk. 53.5°N, 78°E. vi–vii.1982. R.B. Angus. 1♀ (NHMUK). SE Siberia, S Chita reg. 50.071166°N, 115.663446°E. Zun Tore Lake. 30.v.2002. 1 ♂ (JFMC)

IN “O. Ashur-ade” (Ashooradeh Island). 36.91°N, 53.96°Е. Solovkin 1.v.1913. 4, unsexed (ZIN)

KZ: Dzhulek, Orenburg – Tashkent railway, Syr Darja (Taken as Zholek near Baigakum, 44.314°N, 66.466°E), 3♂♂, 3 ♀♀, 9, unsexed (NHML). Kozakhit, Almaty obl. Altyn Emel NP Ul;ken Kalkan. 43.874°N, 78.749°E. N bank of R. Ile, at light, 5.v.2012. D.J. Mann & J. Cooter. 1♂, 3 unsexed (OUM).

UZ: Kyzylkum desert Baymachan distr. 11.v.1995. 2, undissected (NHMUK).

AF: Kabul. 34.548°N, 69.213°E. 1 ♂, 1♀ (NMP), 1♀, 1 unsexed (NMW).

##### Differential diagnosis.

A conspicuously narrow elongate black beetle, with the scutellum wide, generally wider than elytral interstice 2. Elytral interstices weakly raised, not flat as in most other species. Smaller, length 3.7–4.8 mm. Basal line of pronotum completely effaced over middle 1/3 (Fig. 14 b). ♀ with spermatheca smaller (Fig. 26o–s), length ca 0.25 mm.

##### Redescription.

General appearance (Fig. 3a, b). Black, elongate, parallel-sided, and conspicuously narrow. Length: 3.4–4.8 mm, width 1.5–1.9 mm.

Head black with anterior and lateral margins of the clypeus narrowly brown. Anterior margin of clypeus emarginated, the sides of this emargination bluntly rounded. Central part of clypeus bulging upwards, frontoclypeal suture occasionally slightly raised medially but without any trace of a real tubercle, straight or slightly curved backwards over middle 1/2, angled forward to run to the junction of the clypeus and genae laterally. Punctation double, varying from close and strong to rather sparse, generally weaker and sparser in the central area of the frons and becoming rugose anteriorly and laterally (head and pronotum: Fig. 5o, p) Maxillary palpi dark brown, the apical segment blackish. Antennae brown, the clubs darker. Epipharynx (Fig. 8a, b) with the anterior margin of the clithra almost straight either side of the median tylus and the acropariae very well-developed. The prophobae are well-developed on each side of the mesoepitorma and there are three or four chaetopedia on each side. Pronotum black, hemicylindrical, highly arched transversely, only weakly so longitudinally. Entire lateral margins visible from above. Surface with double punctation of variable strength, generally weaker medially. Hind margin bordered only in lateral 1/4s (Fig. 14b). Scutellum rather wide, generally wider than elytral interstice 2, pentagonal, moderately elongate, sides parallel in basal 1/2, then angled to apical convergence. Metaventrite variable. Median diamond-shaped plate sometimes excavated and quite strongly punctured (Fig. 20g), or flatter and more finely punctured (Fig. 20h, i). Legs brown, longer spur of mid tibiae clearly longer than the basal segment of the tarsus (Fig. 18f, g). Elytra glossy black, interstices very weakly convex, finely reticulate and with sparse fine punctation, and 6–8× width of the striae (Fig. 17j).

Aedeagus (Fig. 22y, z) ca 0.8 mm long, apical 1/3 of parameres slightly more expanded than in *A.rutilipennis* and *A.chellala* sp. nov., its outer margin more rounded. Endophallus with fine bristles.

##### Remarks.

*Aphodiuskraatzi* is widely distributed in the drier regions of the western and central Palaearctic, from southern Slovakia to Kazakhstan, Uzbekistan, Afghanistan, and the Chita region of Transbaikal in Russia (Fig. 29b, c). Most specimens are taken at light but Andrey Frolov (St Petersburg) mentions that it can sometimes be found at the roots of grass and other herbaceous plants, as in the area of the Astrakhan oblast’ (Russia) shown in Fig. 28d. He adds that material flying to light often arrives from the direction away from neighbouring water (pers. comm. to RBA, April 2015). In 1982 RBA took one specimen in a water-filled ditch at Karasuk on the Kulundinskaya Steppe in Western Siberia.

#### ﻿ Aphodius (Liothorax) rusakovi

Taxon classificationAnimaliaColeopteraScarabaeidae

Gusakov, 2004

21737574-B261-5B2F-A919-858AE9561D34

Figs 2i , 5m , 14c , 17f , 20m, n , 18h, v , 22p, p ’, 26m, n

Aphodius (Liothorax) rusakovi Gusakov, 2004: 6.

##### Type material examined.

2 paratypes from the Orenburg oblast’, Russia (NHMUK).

##### Additional material examined.

RU: 1 ♀, Ulyanovsk, 54.056°N, 48.519°E (ZIN); 1 ♂, Volgograd oblast’, 48.7°N, 44.5°E, Fastov. leg. Grebennikov.(ZIN).

KZ or UZ 1♀ “Syr-Darja./ B. v. Bodemeyer.” (NMP).

##### Differential diagnosis.

A conspicuously narrow parallel-sided species, comparable with *A.kraatzi* but larger, length 4.5–6.5 mm and base of pronotum completely bordered by a fine impressed line, this sometimes weaker medially (Fig. 14c). Protibial spur of male strongly incurved apically (Fig. 18v). ♀ with spermatheca larger (Fig. 26m, n).

##### Redescription.

General appearance (Fig. 2i). Glossy black, length 4.7–5.5 mm, width 1.9–2.2 mm. Head fairly closely punctate, sometimes slightly rugose on the frons and anterolateral parts of the clypeus, and occasionally rugose over all except the central part of the clypeal bulge. Double punctation scarcely apparent except when the punctation is sparser on the clypeal bulge. Clypeus emarginated anteriorly, edges of this emargination bluntly rounded (head and pronotum: Fig. 5m). Frontoclypeal suture interrupted medially in ♀, in ♂ sometimes with a very weak tubercle, viewed with suitable lighting (Fig. 2i). Epipharynx (Fig. 7i) with anterior margin of clithra almost straight either side of the median tylus and the mesoepitorma scarcely darkened. Prophobae rather strong, grouped towards the margins of the mesoepitorma. Chaetopedia three or four each side. Pronotum highly arched transversely, weakly so longitudinally. Entire lateral margins visible from above (Figs 2i, 5m). Surface with double punctation, this often close though sometimes sparser on the disc. In some specimens the punctures are deeply impressed, so the pronotal surface does not appear smooth. Basal margin entirely bordered, the border rather heavy (Fig. 14c). Elytra noticeably elongate, parallel-sided. Striae narrow but rather deep, strial punctures separated by ca 4× their diameters. Interstices 6–8× the width of the striae, with distinct sparse fine punctation and fine reticulation (Fig. 17f). Apex bluntly rounded. Metaventrite (Fig. 20m, n) with median diamond- shaped plate fairly strongly punctate and no obvious sexual dimorphism. Legs with longer spur of mid tibiae slightly longer than the basal segment (Fig. 18h), and in males the spur of the anterior tibiae is distinctly hooked (Fig. 18v).

Aedeagus (Fig. 22p, p’) length ca 1.1 mm, parameres relatively short and blunt-ended, somewhat downturned apically. Endophallus with scales but no hairs or bristles.

##### Remarks.

Distributed mainly in southern Russia, Ukraine, and Kazakhstan (Fig. 29b, c). Gusakov (2004) lists the holotype and 14 paratypes as having been collected in a dried-out rivulet at Ilek, 120 km SSW of Orenburg on the southern Urals, with two further paratypes from the Rostov and Kharkov districts of Ukraine and four more from the Temirsky region and Uralsk oblast’ in Kazakhstan The Ulyanovsk specimen (ZIN) is labelled as having been taken in a damp saline area (solonchak).

### ﻿The *niger* group

Species of the *niger* group are characterised by the aedeagal endophallus bearing a field of large recurved teeth (Figs 23, 24), clearly visible in cleared preparations where the endophallus is retracted as well as in specimens with the endophallus everted. The apices of the parameres are downturned, though sometimes only weakly so (Figs 23, 24). The basal segment of the mesotarsi may be longer than the longer mesotibial spur, but it is often more or less the same length of the spur or, in some species, clearly shorter than the spur, as in species of the *plagiatus* group (Fig. 18). The *niger* group comprises nine species, of which five are here described as new.

#### ﻿ Aphodius (Liothorax) niger

Taxon classificationAnimaliaColeopteraScarabaeidae

Illiger, 1798

AF8BC118-B8C0-5617-88F1-062974FE8977

Figs 4a , 6e , 9a–c , 15a, b , 17l, m , 18i , 19a , 21a–c , 23a–f , 24 a , 25a , 26t , 31a, b, d, e , 34a–d

Aphodius niger Illiger, 1798: 24. Conserved as valid name: International Commission for Zoological Nomenclature Opinion 2009 (2005: 45).

##### Type material examined.

***Lectotype***, unsexed, “Suec”, designated by Krell et al. (2003) (ZMB).

##### Additional material examined.

SV: Sodermanland, Holo, Tulgarn, Nasuden. 58.95°N, 17.62°E. Trampled bare organic mud. 15.v.2011. Hans-Erik & Livia Wanntorp. 4♂♂, chromosome preps 2: 24.v.2001, 1:25.v.2011, 2: 25.v.2011, 1: 31.v.2011, R.B. Angus; 7♀♀, chromosome preps 3: 24.v.2011, 4: 24.v.2011, 9: 25.v.2011, 2: 31.v.2011, 3: 31.v.2011, 4: 31.v.2015, 5: 31.v.2011, R.B. Angus (NHMUK); 1 ♂ same data, chromosome prep. 2: 24.v.2011 (NMP).

GB: Hampshire, New Forest. Long Slade Bottom. 50.801°N, 1.619°W. Temporary pool after heavy rain. 30.v.2002, R.B. Angus. 4 ♂♂, 4 ♀♀, 5 unsexed. Chromosome preps 1–3: 31.v., 2: 1.vi, 4: 1.vi.2011, R.B. Angus (NHMUK); 1 ♂, same data.(NMP); New Forest, White Moor Pond. 50.820°N, 1.607°W. 2.x.2015, 2.v.2016, R.B. Angus. 8 ♂♂, 7 ♀♀, 3 unsexed. Chromosome preps: 3:x.2015, 5: v.2016. New Forest, Balmer Lawn. 50.828°N, 1.567°W. At edges of pond and ditch. V.2002. R.B. Angus (NHMUK). Inkpen Common, Berkshire 51.375°N, 1.453°W (fide Mann and Garvey (2014), not examined) 1 unsexed (OUM).

RU: Yaroslavl oblast’, Berditsino, 57.454°N, 40.1108°E. leg. Yakovlev. 1♂ (NMW).

##### Differential diagnosis.

**Figure 31.**
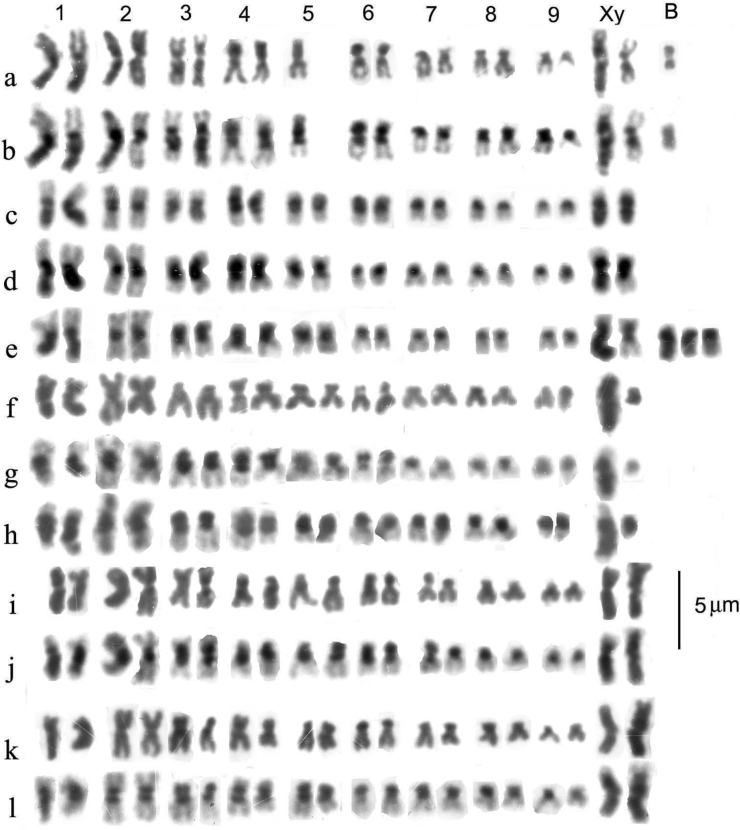
Mitotic chromosomes, arranged as karyotypes **a**, **b***A.niger*, ♂, SV, Tullgarn **a** plain **b** the same nucleus C-banded (1 replicate of autosome 5 missing) **c***A.niger* ♀, SV, Tullgarn, C-banded **d**, **e***A.niger*, ♂♂, GB, Hampshire, New Forest, C-banded **e** with 3 B-chromosomes **f**, **g***A.muscorum*, ♂, HU, Hortobágyi **f** plain **g** the same nucleus C-banded **h***A.muscorum*, ♂, HU, Hortobágyi, C-banded **i**, **j***A.muscorum*, ♀, HU, Kisújszállás **i** plain **j** C-banded **k**, **l***A.muscorum*, ♀, HU, Kisújszállás **k** plain **l** the same nucleus C-banded. Scale bar: 5 μm

*Aphodiusniger* is immediately distinguished from all other *Liothorax* species (and all Aphodiinae whose chromosomes are known) by its long, almost entirely euchromatic, Y chromosome (Fig. 31a, b, d, e). The basal segment of the mesotarsus is always clearly longer than the longer mesotibial spur (Fig. 18i). Elytra glossy black, though interstices have fine reticulation. Central part of metaventrite without reticulation (Fig. 21a–d). Endophallic teeth clearly longer than high. Length of longest endophallic teeth at least 40 μm.

##### Redescription.

General appearance (Fig. 4a). Length 4.2–4.5 mm (♂), 4.1–5 mm (♀), width 1.8–2 mm (♂), 1.8–2.2 mm (♀) (Swedish material), 4.4–4.8 mm (♂), 4.4–5.1 mm (♀), width 2.0–2.2 mm (♂ and ♀) (English Material).

Head black, without obvious brownish margin. Anterior margin of clypeus excised over middle 1/3, the lateral edges of this excision bluntly rounded. Frontoclypeal suture very fine, without any trace of a tubercle, straight-transverse over middle 1/2, angled forward to run to the clypeo-genal junction laterally. The lateral parts of the suture may be almost completely effaced. Clypeus raised in a rounded bulge medially. Surface moderately strongly punctate with double punctation. Larger punctures separated by ca their own diameter over most of the head, but sparser on clypeal bulge. Antennae and palpi blackish brown. Epipharynx (Fig. 9a–c) with the anterior margin of the clithra strongly excised either side of the median tylus and with a few fine acropariae. Chaetopedia stout, 3–6 each side. Chaetopariae ca as stout as chaetopedia, forming a close-set line each side. Apophobae very fine, arranged in a line outside that of the chaetopariae; prophobae vine, quite long, arranged against the sides of the mesoepitorma. Pronotum (Fig. 6e) hemicylindrical, black, highly arched transversely, weakly so longitudinally, lateral parts of surface bulging outwards so that the lateral margins are often not visible from above in their basal 1/3. Surface glossy black, with double punctation, the larger punctures separated by 1–2× their diameter, sparser medially and sometimes petering out in anterior 1/8 where only the fine punctures are present. Scutellum elongate, pentagonal, glossy black with a few sparse punctures. Elytra black, interstices flat, 6–8× the width of the striae, finely reticulate and with sparse fine punctures (Fig. 17l, m), the reticulation stronger towards the apex (Fig. 19a). Striae glossy, without reticulation, with a single row of punctures separated by ca 2× their diameter and excising the strial margins. Metaventrite moderately punctate, the median plate not reticulate, sometimes distinctly concave in males Fig. 21a but sometimes almost flat with a median impressed line, as in females (Fig. 21b, c). Legs blackish brown, mesotarsi always with basal segment longer than longer mesotibial spur (Fig. 18i).

Aedeagus (Figs 23a–f, 24a) length ca 1.2 mm, paramere length ca 0.48 mm, basal piece length ca 0.69 mm, tooth-field length ca 0.67 mm (measurements from the cleared preparation; Fig. 24a). Length of longest teeth on endophallus 54–65 μm. Parameres with the sclerotised strut running from the base just mediad of the outer lateral margin to the inner apical angle. Apical soft pad of parameres rounded apically, not widened.

##### Remarks.

Chromosomally verified material is from the New Forest, England and Södermannland, Sweden. Material from Berditsino, Russia (NMW) is taken to belong to this species. In the New Forest *A.niger* is typically taken at the edges of pools and ditches in April-May, but when large numbers are taken it is almost always due to inwash from the surrounding grassland after heavy rain (Fig. 27a). In Britain, outside of the New Forest, *A.niger* has recently been found at Inkpen Common near Hungerford in Berkshire (Mann and Garvey 2014). In the Swedish locality the beetles were burrowing in trampled organic mud (H.-E. Wanntorp, pers. comm., April 2011) (Fig. 27c). Very occasionally *A.niger* is taken in dung, as by J. Bergsten in the New Forest in 1999, and sometimes in dry areas of the Swedish island of Öland (H. Lundqvist, pers. comm., April 2011). These are rare, random occurrences and may sometimes result from the beetle taking shelter from drought. One further feature of the occurrence of *A.niger* is worthy of comment – it shows dramatic fluctuations in abundance. Thus, in 2002 it was very abundant in its New Forest localities whereas more recently RBA has taken it only in low numbers. Similarly, H.-E. Wanntorp describes the species as being in very high numbers in the locality where he usually only took a few specimens (Fig. 29a).

#### ﻿ Aphodius (Liothoxax) muscorum

Taxon classificationAnimaliaColeopteraScarabaeidae

Ádám, 1994

236DE068-9AE6-5F0B-9E41-85F645D1F480

Figs 4b , 6f , 9e , 15c , 17n-q , 18j , 21e, g, i, j , 23u , 25b , 26u , 31f–l , 34e–g

Aphodius (Liothorax) muscorum Ádám, 1994: 6.

##### Type material examined.

The holotype (Fig. 4b) has five labels: 1: Hung. Borsod-Abaúj-Zemplén m., Aggtelek Vörös-tó, 350 m. 2: Typhetum angustifoliae-latifoliae. 3: iszapból (= from mud) 1989.v.19., leg Ádám L. 4: Paratypus *Liothoraxmuscorum* Ádám, 1994 (A red label, incorrect because Adam mentioned only a single holotype with these data). 5: HOLOTYPE A. (Liothorax) muscorum Adam. R. B. Angus det. 2017. It is a female, so only somatic characters are available for assessment (MNSB).

##### Additional material examined.

HU: Kisújszállás, 47.212°N, 20.700°E, iv.2012, R.B. Angus, 2 ♀♀(NHMUK). Hortobagy National Park, Kis-Kecskés area, 47.67721°N, 21.06067°E. Washed from mud at edge of a lake, and swarming in mud at edge of lake, 47.67772°N, 21.07451°E. 24.iv.2015, 16♂♂, 18♀♀. R. B. Angus (NHMUK, JFM). Aggteleki NP, Josvafö, Szelce-völgy 1♀, 1 unsexed (MNSB). Aggteleki NP, Josvafö, Menes-völgy. 1 unsexed (MNSB). Bukk NP 1♂, 2 ♀♀, 3 unsexed (MNSB). Hortobágyi N.P., 3.vi.1980, leg. U. Göllner, 2 unsexed (ABC).

##### Differential diagnosis.

Closely resembling *A.niger* but clearly distinguished as a separate species by the short, almost dot-like, Y chromosome (Fig. 31f–h). Elytral interstices often with fine but distinct reticulation, giving the surface a leaden-grey silky sheen. Metaventrite of females variously depressed medially, depressed area often partly reticulate (Fig. 21e, g–j).

##### Redescription.

General appearance (Fig. 4b). Length: 4.2–5.2 mm (♂), 4.3–5.2 mm (♀). Width: 1.85–2.35 mm (♂), 1.9–2.3 mm (♀). Black, head, pronotum and scutellum glossy (pronotum sometimes less so), elytra with interstices distinctly reticulate, giving a silky lead-grey sheen. Head: glossy black, rather finely punctate, the punctures separated by 2–3× their diameter. Some slightly larger punctures are present. Frontoclypeal suture very fine, with a glossy impunctate area either side of it. Median 2/3 almost straight, its mid-point and lateral ends slightly raised. Suture angled forward outside the lateral elevations, reaching the margin of the head at the front of the genae. Genae (preocular lobes) protrude laterally at an angle of ca 45˚ in front of eyes, then curve inwards to meet the frontoclypeal suture ca 1.5× eye-diameter in front of eyes. Clypeal margin obtusely angled outward from this point, then curved inwards to the median excision. Median area of clypeus bulging upwards. Genae laterally with a narrow raised margin, this continued across the clypeus, becoming weaker medially. The punctation of the frons is sometimes stronger, with a clear distinction between larger punctures and fine dot-like ones. Antennae and maxillary palpi black to blackish brown. Epipharynx (Fig. 9e) similar to that of *A.niger* (Fig. 9a–c). Head and pronotum (Fig. 6f). Pronotum: hemi-cylindrical, glossy black and with double punctation. The intensity of the punctation is variable, with the larger punctures in some specimens confined to the lateral and posterior 1/4s of its surface, while in others it extends over the whole surface and in some cases both the large and small punctures are heavily impressed, giving a roughened appearance to the surface. There is often an ill-defined narrow median band more or less free of punctures, extending from behind the middle of the disc almost to the hind margin. Lateral margins with a distinct raised bead, this either black or dark brown and extending round the hind corners often giving a continuous narrow raised border over the entire basal margin. It is necessary to tilt the specimen to trace the full course of this border, which is sometimes absent either side of the median 1/3, or from the middle 1/3 of the margin (Fig. 15c). Lateral margins visible from above over the anterior 1/2, behind this varying from being completely visible to totally obscured by the bulging pronotal surface. Scutellum: elongate, sides parallel in basal 1/2, then angled inwards to bluntly rounded apex. Surface glossy black, with sparse fine punctures. Elytra: black but with lead-grey silky sheen resulting from fine but distinct reticulation on the interstices. The strength of the reticulation varies from very weak in the holotype (Fig. 17n) to rather stronger in some Hortobágyi material (Fig. 17o) and stronger still in the two females from Kisújszállás (Fig. 17p, q). This reticulation may be obscured if the specimen is at all greasy. Interstices flat, ca 6× wider than the striae, with very fine punctures, these ca the same size as the meshes of the reticulation. Striae narrow, vertical-sided, their bottoms glossy. Strial punctures in single rows, small, separated by ca 3× their diameter, and excising the strial margins.

Metaventrite: median diamond-shaped plate in male finely and sparsely punctate, concave, depressed to median longitudinal groove (Fig. 21g); in female variable, sometimes median longitudinal groove flattened, but with the median area depressed anteriorly and sometimes with some reticulate sculpture (Fig. 21i, j).

Legs dark brown, often blackish, basal segment of mesotarsi normally longer than longer mesotibial spur (Fig. 18j) but sometimes equal in length or slightly shorter.

Aedeagus: Fig. 23u. Endophallus with heavy recurved teeth. Length ca 1.06 mm, paramere length ca 0.54 mm, basal piece length ca 0.69 mm. Length of tooth-field ca 0.57 mm, of longest teeth on endophallus 47–58 μm.

##### Remarks.

As already mentioned, Ádám described *A.muscorum* as a new species, intending it to be the valid name for the species then known as Aphodius (Liothorax) niger (Panzer,1797). However, chromosomal research by RBA has shown that Hungarian material is in fact different from true *A.niger*, so Ádám’s *Liothoraxmuscorum* must be regarded as a valid species. Ádám gives a description which, from its reference to variation in the strength of the elytral sculpture, is based on a fairly wide selection of Hungarian material. However, he lists only one type specimen, the holotype from the lake Vörös-tó in the Aggteleki national park in NE Hungary, adding that he took the unsexed specimen from mud in a drying out *Typha* bed. Vörös-tó is now a fairly deep lake with limited development of *Phragmites* round its edge. It was dredged out when the nearby entrance to the Baradla Barlang (cave) was developed (Sandor Rozsa, Chief Warden of the National Park, pers. comm. to RBA, April 2012). The nearby Aggteleki-tó has extensive development of mud and *Typha*, but though RBA has taken A. (Nialus) varians in both lakes he failed to find the *Liothorax*. It is therefore necessary to rely on the holotype and a limited amount of material from the neighbourhood, in the MNSB, for comparison with extensive material from the Hortobágyi National Park, from which chromosomes and DNA are available.

*Aphodiusmuscorum* stat. nov. is at present reliably identified only from Hungary. It appears to be a steppe species which may have a wider distribution eastwards but this needs to be verified with *A.niger* s.l. material from adjacent areas further east (Fig. 29a). RBA took the species abundantly in lakeside mud in the Hortobágy National Park in April 2015 (Fig. 27b), and 2 specimens in the mud of a drying out ditch near Kisújszállás in April 2012.

###### ﻿*Aphodius* "cf *A.niger*": species resembling *A.niger* and *A.muscorum*, but of uncertain identity

**Material examined.** RU: Volgograd, 48.739°N, 44.529°E. 1♂ (NMP). Tyumen. 57.215°N, 65.574°E. 1♂.(NMP).Tuyanso, Chernomorsk oblast’. 44.310°N, 30.517°E. 1915. 1♂ (ZIN)

KZ: 10 KM SE of Akmolinsk (Astana) 51.085°N, 71.720°E. Under detritus by the water. 5.v.1957. E. L. Gurjeva. 1 ♀ (ZIN). Akmola obl. Kokshipan. By R. Tirs, Marsh on floodplain with *Ranunculus* and *Fritillaria*. 13.v.1957. E.L. Gurjeva. 1♂ (ZIN). Akmola oblast’, dolina Basaga-ozik., 49.840°N, 65.991°E. In a Solonchak (saltmarsh). L. Arnoldi, 27,v.1957.(ZIN); Uralsk 51.214°N, 51.271°E. 1♂ (NMP).

CZ: Hradec Králové, Na Plachtě. 50.186°N, 15.859°E. 1♂, 1 unsexed (NHMUK). České Budějovice, Hosín aerodrome. 2 unsexed (NHMUK). Břeclav Pohansko 48.73°N, 18.918°E. Martinů Ivo leg. 20.iv.2008. 1♂, 4 ♀♀, 4 unsexed (NHMUK).

SK: Bol. 48.494°N, 21.949°E. 2 unsexed (NHMUK).

AU: Feldkirch Moosbrugger Vbg. 47.258°N, 9.581°E. 1♂, 1♀(JHMC). Purgstall a. E. Austria inf. 48.063°N, 15.132°E & 48.064°N, 15.133°E. 2, unsexed. Leg. Ressi (JFMC). Umgebung Wien Mauerbach, 48.246°N, 16.165°E & 48.264°N, 16.354°E. 2, unsexed (JFMC). Wien Umgebung, 48.209°N, 16.392°E. 1, unsexed (JFMC). Neusiedler See. 47.712°N, 16.16.804°E 1, unsexed (JFMC). Ullig Hart bei Graz, 47.082°N, 15.440°E. 1, unsexed (JFMC).

GE: Masselund, Emsland, 8.5.1969, Kerstens 1 unsexed; Hasslünne, Emsland, 8.5.59, Kerstens, 1 unsexed (ABC); Garz Dr Minarz, “594 niger”, 1 unsexed; c. Epplsh Steind. d., 1♂, 6 unsexed (NMW).

PL: Hinter Pommern Kołobrzeg (Zieleniewo), 1 unsexed (MNCN).

RU: Yaroslavl oblast’, Berditsino, 57.454°N, 40.1108°E. leg. Yakovlev. 1♂ (NMW).

**Remarks.** Material from France, Italy, Bosnia, and Turkey is listed by Maté and Angus (2005). It is not listed here but is mapped (Fig. 29).

Material from coastal or near-costal localities from Poland, Germany, and France is likely to be *A.niger*. The Yaroslavl specimen is taken as *A.niger* because locality seems to be ecologically similar to the English and Swedish ones. This might also be true of the Tyumen male.

The specimens from Břeclav Pohansko and Bol are likely to be *A.muscorum* because these lowland localities are on the edge of the Hungarian plain, and the same is true of all the eastern Austrian material.

The outstanding problem with Central European material is the identity of the “*A.niger*” from the German Mittelgeberge and the northern and central parts of Czechia, as well as the French Massif Central. The map given by Rößner (2012) shows German records, mainly pre-1950, concentrated either in coastal regions or somewhat further inland (the Mittelgeberge) with few records connecting the two areas. Rößner et al. (2016) give updated records, with some west German localities in the Mittlegeberge post 1950. Costessèque (2005) records it from all the Départements of the Massif Central, but RBA failed to find it in the southern Auvergne (Cantal, Haute-Loire) in April 2017. While all the material may be *A.niger*, there remains the possibility that a further, as yet unknown species may be present. We have, unfortunately, not been able to obtain living material from this inland area.

The material from Astana and the Akmola oblast and Uralsk may include a new species characterised by a reticulate metaventrite in the males, but a male from a saline depression (Solonchak) by the river Basaga-ozik lacks any trace of reticulation. The Volgograd male also lacks any trace of reticulation on the central portion of the metaventrite. The males from the Chernomorsk oblast’ and Volgograd also lack any reticulation on the metaventrites and their identities are unclear.

#### ﻿ Aphodius (Liothorax) felix sp. nov.

Taxon classificationAnimaliaColeopteraScarabaeidae

EE13BC0C-5048-59B7-9CEF-09240294C48E

https://zoobank.org/79A36FAF-5DDF-45AC-99BB-A9BC5CDBEC67

Figs 4c , 6i , 8e, f , 15d , 16v , 18p, q , 21f, k , 23v, w , 25c , 26v , 32a–e

##### Type material examined.

***Holotype*** ♂: Italy, Abruzzo, Campo Felice. Washed into pool. 1.vi.2009. R. B. Angus. Chromosome prep. 4, 7.vi.2009. R. B. Angus (NHMUK). ***Paratypes***: 1♂, data as holotype, chromosome prep. 3, 7.vi.2009. R. B. Angus; 3♀♀, data as holotype, chromosome preps 1 – 3, 9.vi.2009. R. B. Angus (NHMUK); 1♀, data as holotype, 1.vi.2009 (sequenced) (JFMC); Basilicata, M. Sirino (PZ) XI.1997 1400–1700m Leg. F. Angelini (JFMC).

##### Differential diagnosis.

**Figure 32.**
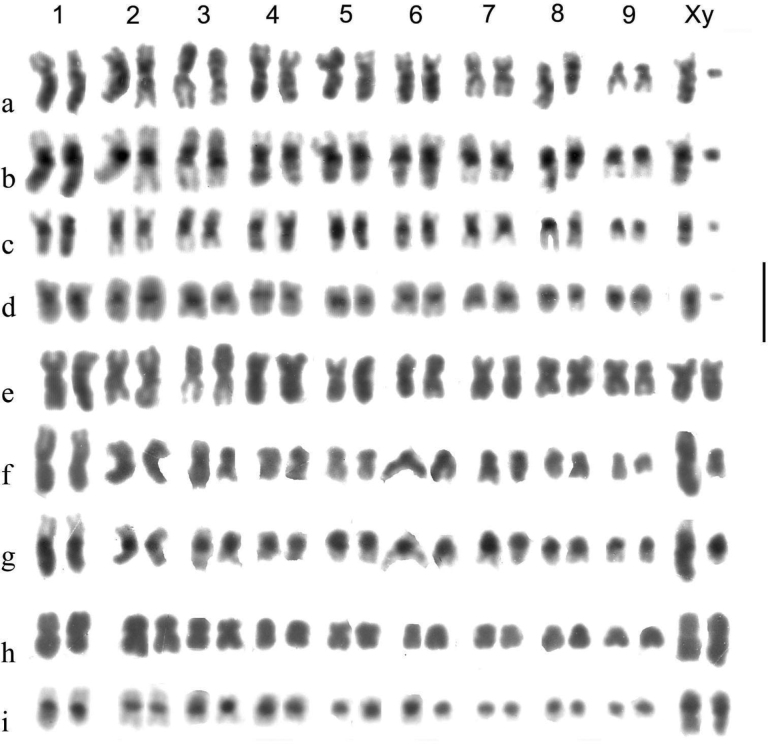
Mitotic chromosomes arranged as karyotypes **a–e***A.felix* sp. nov. IT, Abruzzo. Campo Felice **a**, **b** holotype ♂ **a** plain **b** the same nucleus C-banded **c** holotype ♂ C-banded, autosome 8 polymorphic, 9 subacrocentric **d** paratype ♂, C-banded, autosomes 8 and 9 metacentric **e** paratype ♀ **f**–**i***A.bellumgerens* sp. nov., paratypes, IT, Sicily **f**, **g***A.bellumgerens* sp. nov., ♂, paratype **f** plain **g** C-banded **h**, **i***A.bellumgerens* sp. nov., ♀, paratype **h** plain **i** C-banded. Scale bar: 5 μm

*Aphodiusfelix* sp. nov. was the first new species to be recognised, after *A.wilsonae*, initially because of its small y chromosome. The endophallic teeth are large, as in *A.niger*, and the basal segment of the mesotarsi is not longer than the longer mesotibial spur. The pronotum is fairly strongly punctate and the sides bulge outwards so that the lateral margins are not visible from above either all the basal 1/2 or visible again in the basal 1/6 (Fig. 6i), a character shared with Sicilian *A.bellumgerens* (Fig. 6j). Chromosomally, these two species are clearly distinguished by their X chromosomes, the X being clearly the longest in the nucleus in *A.* (*bellumgerens* Fig. 32f–i), but clearly shorter than autosome pairs 1–3 in *A.felix* (Fig. 32a–e).

##### Redescription.

General appearance (Fig. 4c). Length: 4.4–4.5 mm (♂), 4.2–5.0 mm (♀); width: 1.9–2.0 mm (♂), 1.6–2.3 mm (♀). Glossy black, tarsi, and tibial spurs dark brown, tibiae with a hint of brown-bronze reflections.

Head with dorsal surface domed, flatter behind frontoclypeal suture. Frontoclypeal suture distinct, completely non-tuberculate but with smooth elevated areas each side of the median straight section and a weaker elevation medially. Genae rounded, strongly protuberant laterally in front of eyes. Clypeus with rounded median emargination, angles either side of this rounded. Clypeus and genae with strong raised margin, this slightly brownish. Surface strongly and more or less evenly punctuate, the punctures separated by ca 1.5× their diameter, and finer medially. Antennae and palpi more or less black. Epipharynx (Fig. 8e, f) clithra evenly excised either side of the median tylus, chaetopedia with four or five rather stout spines and spines of chaetopariae mainly shorter than chaetopedia. Head and pronotum as in Fig. 6i.

Pronotum hemicylindrical, highly arched transversely but more or less flat longitudinally, lateral margins more or less parallel, but slightly convergent anteriorly, weakly and very evenly curved. Pronotal surface bulging outwards from the general curvature over all or part of the basal 1/2 of the pronotum, ca 1/4 of the way in from each side, so that the lateral margins are not visible from above either in all of the basal 1/2 of the pronotum, or visible in the basal 1/6 (Fig. 6i). Lateral margins with distinct raised border, this continued very finely over the lateral 1/3 of the basal margin, the median 1/3 of which normally lacks any border Occasionally the median 1/4 of the basal margin is bordered but on either side of this is an unbordered section amounting to one 1/4 of the length of the basal margin (Fig. 15d). Anterior margin without any trace of a raised border. Surface with double punctation of variable strength. Larger punctures separated by 2–4× their diameter, sometimes very strongly impressed and with the pronotal surface depressed immediately round their edges, but sometimes more moderate and with the pronotal surface evenly curved around them. Finer punctures dot-like, in some specimens separated from one another by ca 2× their diameter but in other specimens much sparser, separated by 4–6× their diameter. Pronotal surface between the punctures smooth, glossy. Scutellum: elongate, pentagonal, ca 10% of elytral length, glossy black with brownish lateral and apical edges, and with sparse punctures medially. Elytra: glossy black but the interstices slightly duller than head and pronotum and with fine isodiametric reticulation (visible at × 40 magnification with bright diffuse illumination) (Fig. 16v). Striae narrow (ca 1/5 the width of the interstices), vertical sided and with punctures separated by ca 2× their diameter. In the two ♂♂ the punctures bulge into the interstices but in the three ♀♀ they hardly deflect the strial margins. Lateral margins of the elytra distinct, at base strongly upcurved in front of the humeral bulges; stronger basally and at apex ca as wide as stria 2. Interstices 4× as wide as striae, with fine sparse punctation, this a bit stronger in the humeral area in front of abbreviated stria 9.

Metaventrite: median diamond-shaped plate fairly strongly punctate, concave to the depressed mid-line (2 ♂♂) (Fig. 21f, k), flat with faint median impressed line to median concavity over middle 1/3 of the diamond-shaped area (3 ♀♀). Legs: black with dark brown tarsi and tibial spurs, rather long and slender, basal segment of hind tarsi as long as segments 2 + 3 + 1/2 of segment 4. Longer spur of mid tibiae longer than basal segment of mid tarsi, though sometimes the segment is almost as long as the spur (Fig. 18p, q).

Aedeagus (Fig. 23v, w): size and shape of parameres as *A.niger*, and spines of endophallus strong as in *A.niger*. Longest teeth on endophallus 45 μm long.

##### Etymology.

*felix* – Latin, happy, named from the type locality, Campo Felice.

##### Remarks.

As yet this species is known only from the type series. The beetles were taken at the edge of a pool below a grassy slope on Campo Felice (Fig. 29a) in the Abruzzo mountains (Fig. 28f) after a day of continuous heavy rain. A return visit a day or so later, after better weather, yielded no further specimens, so that it seems that this species lives at the roots of the grass and other herbs rather than in the mud at the water’s edge. Specimens from northern and central Italy mentioned in Maté and Angus (2005) are most likely *A.felix*. See Remarks after *A.bellumgerens*.

#### ﻿ Aphodius (Liothorax) bellumgerens sp. nov.

Taxon classificationAnimaliaColeopteraScarabaeidae

C85001FB-6131-5D4F-BAF4-B413BEB88D37

https://zoobank.org/892D4998-D52B-4B6B-BF0D-ADA21A086F2F

Figs 4f , 6j , 9f, g , 16d , 17u , 18r , 21l, m, p–t , 24m, n , 25e , 26z , 32f–i

##### Type material examined.

***Holotype*** ♂: Sicily. Parco delle Madonie. Piano Battaglia. At edges of pool. 1.v.2013. R.B. & E.M. Angus. Chromosome prep. 7: 8.v.2013 (NHMUK). Length 3.9 mm, width 1.8 mm. ***Paratypes***: 7♂♂, 8♀♀, same data as holotype. Chromosome preparations 1–8, 7.v.2012, 1–6 and 8, 8.v.2012 (NHMUK). 7♂♂, 5♀♀, same data as holotype but 23.iv.2018 (NHMUK), 1♂, 1♀, same data (NMP); 12♂♂, 6♀♀, Sicily, Nebrodi, Monte Soro, 1600m a.s.l. 25.iv.2018. R.B. & E.M. Angus. Chromosome preparations 1–4, 27.iv.2018, 1–5, 1.v.2018, 1–5, 2.v.2018, 1–4, 10.v.2015 (NHMUK). 1♂, Sicily, Nebrodi, leg F. Krell (DMNS). 3, data as holotype (sequenced) (JFMC); 1 SICILY, Nebrodi, Monte Soro, 1600m a.s.l. 25.iv.2018. R.B. & E.M. Angus. Chromosome preparation 4, 27.iv.2018 (Sequenced) (JFMC).

3♂♂, 4♀♀, 37 unsexed: ITALY, Sicily, Monte Soro, Tumpelufer, welkes Laub. 1800 m NN 30.5.1993 leg. U Schaffrath (ABC & JFMC).

##### Differential diagnosis.

Most similar to *A.felix*, from which it is shown to be different because of its clearly larger X chromosome (Fig. 32f–i, a–e). The slightly smaller aedeagus, with the endophallic tooth-field clearly shorter than the parameres (Figs 23p–t, 24m, n) is also distinctive.

##### Description.

General appearance (Fig. 4f). Length: 3.6–4.4 mm (♂), 4.0–4.8 mm (♀), width: 1.65–1.95 mm (♂), 1.7–2.0 mm (♀). Glossy black, apical segments plus claws of tarsi dark brown.

Head with no trace of reticulation but with sparse double punctures, the punctures separated by ca 2× the diameter of the larger punctures. Frontoclypeal suture indistinct but present, without tubercles but slightly raised at ends of median section, before turning to run to the anterior margins of the genae. Anterior margin of clypeus emarginated medially, rounded at sides of the emargination. Antennae and palpi black. Epipharynx (Fig. 9f, g) with anterior margin of clithra emarginate each side of projecting median tylus, with a few fine acropariae. Chaetopedia 4 or 5 each side, stout. Chaetopariae forming a single line each side, closely set, stout. Apophobae forming a narrow band of fine setae outside the chaetopariae, prophobae in a narrow field each side of the mesoepitorma. Head and pronotum (Fig. 6j): pronotum: surface either smooth and glossy as head or slightly dull, with double punctation, size of the large punctures stronger than on head, but variable, these punctures separated by ca 2× their diameter. Small punctures tiny dots. Mid-line impunctate or with very fine punctures. Pronotum more or less hemicylindrical, highly arched transversely, in mid-line weakly arched longitudinally. Sides of pronotum bulging outwards in basal 1/3, so that the basal 1/3 of the lateral margins is not visible from above. Lateral margins completely bordered, this border extending along lateral 1/3 of basal margin as a very fine impressed line. Median 1/3 of basal margin without an impressed border, as in the *A.wilsonae* shown in Fig. 16d. Scutellum pentagonal, sides parallel in basal 1/2, then converging to blunt apical point. Elytra: sides either almost parallel over basal 2/3 or more distinctly rounded to widest point 2/3 of the way from the base, bluntly rounded to apex. Interstices flat, ca 6× the width of striae, glossy black, sometimes with very fine reticulation, especially over basal 1/4 (Fig. 17u). Sutural interstices weakly raised to suture. Striae narrow, shallow, the strial punctures scarcely impinging on their margins. Median diamond-shaped plate of metaventrite with sparse fairly fine punctures, sometimes flattened in males or with a median depression in females (Fig. 21l, m).

Legs: Long spur of mid tibiae clearly longer than basal segment of tarsi, exceeding it by a distance approximately equal to the width of the basal margin of the second tarsal segment (Fig. 18r). Spines on apical margin of posterior tarsi short and even on outer/ventral face, longer, sparser, and more irregular on inner/dorsal face.

Aedeagus (Figs 23p–t, 24m, n) Length ca 0.9–1.1 mm, paramere length ca 0.43–0.47 mm, basal piece length ca 0.5–0.6 mm, tooth field length ca 0.3–0.4 mm. This is one of the few species in which the length of the endophallic tooth-field is less than that of the parameres. Longest teeth on endophallus ca 47 μm long.

##### Etymology.

*bellumgerens* – Latin, waging war, adjective, named for the type locality, Piano Battaglia (the battlefield).

##### Remarks.

So far known only from Sicily (Fig. 29a). Many of the types were taken by swishing the water at the edges of the pool on the Piano Battaglia, washing the beetles from the banks so that they floated on the water. This pool dries out completely in the summer. The Monte Soro specimens were floating and clinging to plants in a shallow pool, following recent rain (Fig. 27g). We have seen specimens from Calabria (Sta Christina, Cippo di Garibaldi) which are apparently morphologically identical but have not been included in the type series due to the scarcity of material and lack of karyological or molecular data. RBA has recently received living material from Calabria: Villaggio Mancusa, 39.10727°N, 16.64085°E, from which chromosomes indicating *A.felix*, not *A.bellumgerens* have been obtained. These data will be published in due course.

#### ﻿ Aphodius (Liothorax) bameuli sp. nov.

Taxon classificationAnimaliaColeopteraScarabaeidae

89FA22F1-B783-56FA-8226-4DE0E8904C5A

https://zoobank.org/F188AEBA-0E15-4EB4-B1AD-A1A49DBBA81E

Figs 4d , 6g , 8g , 16a, x , 18k, l, u 21p–r, 23j–l, 24b, 25f, 26w, x, 33a–d, 34h

##### Type material examined.

***Holotype*** ♂: Corsica, Haute-Corse, pelouse (lawn) grassland by the Lac de Melo. Cow dung. 19.vi.2011. R. B. and E. M. Angus. Chromosome prep. 2, 29.vi.2011. R. B. Angus (NHMUK). ***Paratypes***: 2♂♂, data as holotype, 1 labelled Chromosome prep. 1, 29.vi.2011. R. B. Angus; 4♂♂, data as holotype but 24.vi.2011; 14♀♀, data as holotype, including Chromosome preps 1–4, 28.vi.2011, 6–9, 28.vi.2011 and 1–3, 1.vii.2011, R. B. Angus; 1♀, data as holotype (NMP); 11♀♀, data as holotype but 24.vi.2011; 2♂♂, 5♀♀, data as holotype but 30.vi and 1.vii.2009, R. B. and E. M. Angus. ♂ Chromosome preps 1 and 2, 6.vii.2009, ♀ Chromosome preps 3–5, 5.vii.2009 (NHMUK). 1♂ data as previous (NMP); 1♂, data as holotype but 24.vi.2011, in F. Krell collection (DMNS). 3 Corsica, Haute-Corse, pelouse (lawn) grassland by the Lac de Melo. Cow dung. 19.vi.2011. R. B. and E. M. Angus (sequenced) (JFMC); 7 ♂♂, 23 unsexed, “18.vii.1983. Corse. Lac de Nino 1743 m. Rinderkot. Leg. H. Fery”. In H. Fery collection (ZSM); 2 unsexed, “France Corse Nino-See 1730 m. 12.vii.1987. Leg H. Hirschfelder.” In H. Fery collection (ZSM). In CZC and JFMC: 1♂ 1♀, 1 unsexed France: Corsica Plateau d´Alzo 1500m 42°16'N, 9°04'E 21/V/1994 leg. C. Zorn.

##### Differential diagnosis.

The parallel-sided, hemicylindrical, lightly sculptured appearance (Fig. 4d) is distinctive. The pronotal sides bulge outwards laterally so that the lateral margins are not visible from above in basal 1/2 of the pronotum. The lateral margins themselves are parallel, only very slightly convergent anteriorly. The general shape of the beetles and their pronotal configuration resembles those of *A.niger* and *A.muscorum* (Fig. 4a, b), but these species are more heavily sculptured.

##### Description.

General appearance (Fig. 4d). Length: 4.0–4.6 mm (♂), 3.9–4.8 mm (♀); width: 1.7–1.9 mm (♂), 1.6–2.0 mm (♀). Glossy black, tarsi, tibial spurs dark brown, femora and tibiae dark brown with black-bronze reflections. Head: dorsal surface domed, flatter behind frontoclypeal suture. Medial area of clypeus more demarcated as a dome than in *A.felix*. Frontoclypeal suture distinct, completely non-tuberculate but with distinct smooth elevated areas each side of the median straight section and a weaker elevation medially. Genae rounded, strongly protuberant laterally in front of eyes. Clypeus with rounded median emargination, angles either side of this rounded. Clypeus and genae with strong raised margin, this slightly brownish. Surface strongly and more or less evenly punctuate, the punctures separated by ca 1.5× their diameter, and finer medially. Antennae and palpi more or less black. Epipharynx (Fig. 8g) clithra evenly excised either side of the median tylus, chaetopedia with 4–6 spines, these slightly less robust than those of *A.felix* and spines of chaetopariae as long as or longer than chaetopedia. Head and pronotum as in Fig. 6g. Pronotum hemicylindrical, highly arched transversely but more or less flat longitudinally, lateral margins more or less parallel, but very slightly convergent anteriorly, weakly and very evenly curved. Pronotal surface bulging outwards from the general curvature over the basal 1/2 of the pronotum, ca 1/4 of the way in from each side, so that the lateral margins are not visible from above in the basal 1/2 of the pronotum (Fig. 6g). Lateral margins with distinct raised border that continued very finely over the lateral ca 1/3 of the basal margin, at least the median 1/3 of which lacks any border (Fig. 16a). Anterior margin without any trace of a raised border. Surface with double punctation of variable strength, but in general weaker than in *L.felix* sp. nov., though the strength of the punctation of the two species overlaps. Larger punctures separated by 2–4× their diameter, sometimes very strongly impressed and with the pronotal surface depressed immediately round their edges, but sometimes more moderate and with the pronotal surface evenly curved around them. Finer punctures dot-like, in some specimens separated from one another by ca 2× their diameter but in other specimens much sparser, separated by 4–6× their diameter. Pronotal surface between the punctures with a silky sheen and a very fine, generally indistinct, small, isodiametric reticulation.

Scutellum elongate, pentagonal, ca 10% of elytral length, glossy black with brownish lateral and apical edges, and with sparse punctures medially.

Elytra glossy black but the interstices slightly duller than head and pronotum, appearing slightly silky and with a very fine indistinct isodiametric reticulation sometimes visible. Striae narrow (ca 1/5 the width of the interstices), vertical sided and with punctures separated by ca 2× their diameter. In the two ♂♂ the punctures bulge into the interstices but in the 3 ♀♀ they hardly deflect the strial margins. Lateral margins of the elytra distinct, at base strongly upcurved in front of the humeral bulges; stronger basally and at apex ca as wide as stria 2. Interstices 4× as wide as striae, with fine sparse punctation (Fig. 16x), which is a bit stronger in the humeral area in front of abbreviated stria 9.

Legs (Fig. 18k, l, u) dark brown with bronze-black reflections and tarsi and tibial spurs brown, rather long and slender, basal segment of hind tarsi as long as segments 2 + 3 + 1/2 of segment 4. Longer spur of mid tibiae longer than basal segment of mid tarsi.

Metaventrite (Fig. 21p–r): median diamond-shaped area rather lightly punctate, without pubescence, its mid-line either almost indistinguishable or variably depressed, sometimes forming a distinct longitudinal furrow up to 1/3 as wide as the median point of the diamond. There is no sexual dimorphism.

Aedeagus (Figs 23j–l, 24b) Length ca 1.2 mm, paramere length ca 0.45 mm, basal piece length ca 0.76 mm, length of endophallic tooth-field ca 0.4 mm. Length of longest teeth on endophallus ca 44 μm.

##### Etymology.

Named after our good friend Dr Franck Bameul of Bordeaux, who accompanied RBA and EMA to the Lac de Melo on the 24.vi.2011 and was with us as we all endured a prolonged and spectacular thunderstorm.

##### Remarks.

*Aphodiusbameuli* sp. nov. is endemic to Corsica (Fig. 29a) and, as far as we know, is the only *Liothorax* definitely associated with dung. On the Melo lawns (Fig. 27e) it occurred in cow pats, avoiding the more liquid regions towards the centre of the dung. It ate the dung, which filled the guts of specimens used for chromosome work. Hans Fery (pers. comm. ix.2015) told us that when he collected it by the Lac de Nino the water was very high, covering the lakeside lawns but that the cow pats protruded above the water surface and that is where the beetles were found. RBA and EMA failed to find it in the cow dung on the lawns in the valley of Pozzi (Ghisoni), further south.

It is an elongate, parallel-sided, and lightly sculptured species.

#### ﻿ Aphodius (Liothorax) krelli sp. nov.

Taxon classificationAnimaliaColeopteraScarabaeidae

121D8F68-75C2-5860-934C-05AC5878D13E

https://zoobank.org/40001D30-4534-4350-97CB-5E286675ADC8

Figs 4e , 6h , 8j , 16b , 17y , 18m, n , 23m, o , 26y , 33e–l , 34i, j

##### Type material examined.

***Holotype*** ♂: Sardinia, Nuoro Province, Badde Salighes 3.iv.2012. R. B. & E. M. Angus. L 5.0 mm, b 2.1 mm. Chromosome prep. 2, 5.iv.2012 (NHMUK). ***Paratypes***: 4♂♂, 4♀♀, data as holotype, but 1♂ taken on 1.iv.2012. ♂ paratypes with chromosome preparation data prep. 5: 5.iv.2012 and prep. 1: 10.iv.2012, ♀ paratypes with data preps 3, 4, 6–8: 5.iv.2012 (NHMUK); 1♀, data as holotype (NMP) 1♀, data as holotype (JFMC); 1♂, chromosome prep 5, 5.iv.2012, data as holotype (DMNS); 1♀, Sardinia, Sassari, Ciaccia, 17.VII.2008, old sheep dung, R. B. Angus (JFMC); 2 unsexed, ITALY, Sardegna, Nuoro, Altopiano della Campeda, 40°21.245'N, 8°47.044'E, 580m, 18.5.2005 leg. Starke (ABC); 1 unsexed, [ITALY], Badde Salighes Sardegna [Nuoro Pr], Aphodius (Nialus) niger Carpaneto det. 1982 (MNHG).

##### Differential diagnosis.

**Figure 33.**
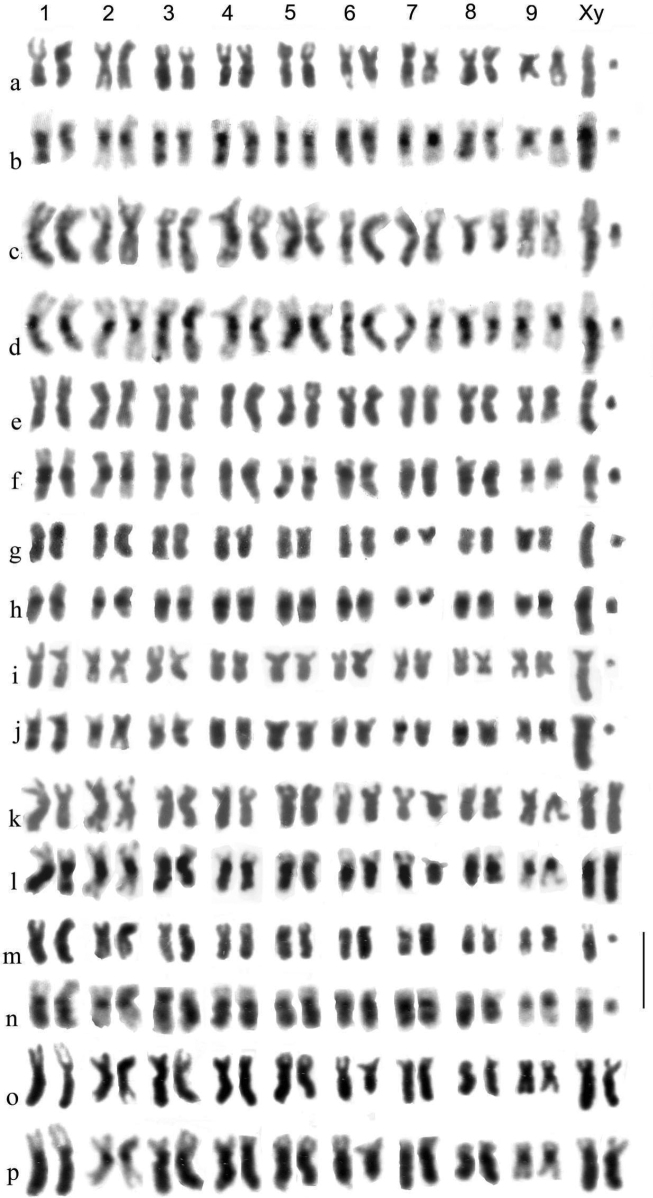
Mitotic chromosomes arranged as karyotypes **a–d***A.bameuli* sp. nov., ♂ paratypes, FR, Corsica, by the Lac de Melo. Autosome 9 polymorphic in **a**, **b** homozygous submetacentric in **c**, **d**. **a**, **c** plain **b** and **d** the same nuclei C-banded **e–l***A.krelli* sp. nov., types, IT, Sardinia **e**, **f** paratype ♂ with the heterochromatic long arm of autosome **1** weakly developed **e** plain **f** the same nucleus C-banded **g**, **h** holotype with the heterochromatic long arm of autosome **1** fully developed **g** plain **h** the same nucleus C-banded **i**, **j** paratype ♂ with both replicates of autosome 7 lacking the heterochromatic long arm **i** plain **j** the same nucleus C-banded **k**, **l** paratype ♀ with autosome 9 heterozygous for an inversion polymorphism **k** plain **l** the same nucleus C-banded **m–p***A.wilsonae* SP **m**, **n** holotype ♂ **m** plain **n** the same nucleus C-banded **o**, **p** paratype ♀ from Balneario de Corconte **o** plain **p** the same nucleus C-banded. Scale bar: 5 μm.

Sardinian *A.krelli* differs from Corsican *A.bameuli* in being less parallel-sided (Fig. 4e, d) and with the pronotum more heavily punctate (Fig. 6h, g). The karyotypes of the two are very similar in the sizes and shapes of their chromosomes (Fig. 33e–l) but the long arm of autosome 7 in *A.krelli* is heterochromatic and is polymorphic for a deletion – it may be present (Fig. 33f, i–l) or absent (Fig. 33g, h).

##### Description.

General appearance (Fig. 4e). Length: 4.4–5.0 mm, width: 2.0–2.1 mm (♂), 4.1–4.5 mm, width 1.7–2.0 mm (♀). Head: glossy black with no trace of reticulation, anterior margin vaguely browner. Sculpture of double punctation, the fine punctures separated by ca 2× their width, the coarser punctures ca 3× the diameter of the fine ones, distributed over the anterior and lateral parts of the head and on the frons. Frons elevated in a vague hump on disc. Frontoclypeal suture very fine but distinct and complete. Anterior margin of clypeus emarginated medially, the margin rounded either side of this emargination. Anterior and lateral margins of head bordered, the border extending from the back of the genae. Genae distinct, fairly abruptly divergent anteriorly from anterior margin of the eyes, widest point behind middle of genae, in front of which the genal margins curve more gently to the clypeus, which they meet at a slight angle. Epipharynx (Fig. 8j) with anterior margin of clithra strongly emarginate each side of the median tylus, and with a few fine acropariae. Chaetopedia stout and long, four or five each side; chaetopariae closely set, fairly stout, forming a line each side. Prophobae very fine, clustered along the edges of the mesoepitorma; apophobae fine and long, arranged in a line outside the chaetopariae. Antennae and palpi blackish brown. Head and pronotum as in Fig. 6h. Pronotum: hemicylindrical, highly arched transversely but very weakly so longitudinally in the mid-line. Lateral margins entirely visible from above. Lateral margin bordered, basal margin with very fine border, this sometimes complete but usually absent from the median 1/2 to 1/3 of the margin. Surface glossy black with no hint of reticulation but with double punctation. Fine punctures dot-like, extending over the whole surface, coarser punctures ca 4–8× the diameter of the dots, sparser and tending to be smaller on the disc. Fine punctures separated by ca 4–6× their diameter, coarse ones by 1.5–2.5× their diameter.

Scutellum: narrow, pentagonal, sides parallel in basal 1/2 then convergent to a blunt point apically. Surface glossy black with a few punctures in basal 1/2.

Elytra: elongate, not quite parallel-sided, widest just behind middle, then tapered to bluntly rounded apex. Ground colour of interstices silky black with very fine faint isodiametric reticulation (Fig. 17y). Striae glossy black. Interstices flat, ca 10× the width of the striae, with scattered fine punctures separated by ca 4–5× their width. Sutural interstices weakly raised longitudinally from their external margins to ca 1/3 of the way to the suture, then depressed to the suture. Striae narrow, with vertical sides, with a single row of punctures separated by ca 3× their diameter, the sides of these punctures encroaching into the strial margins. Metaventrite with median diamond-shaped area fairly strongly punctate, the punctures larger than in *A.bameuli*, in males concave to median impressed furrow, in females often flatter.

Legs: dark brown, longer spur of mid tibiae clearly longer than basal segment of tarsus, although sometimes only slightly so (Fig. 18m, n). Post tibiae with apical fringe of spines short and of even-length ventrally, longer, sparser and of uneven lengths dorsally.

Aedeagus: Fig. 23m–o. Length ca 1.1 mm, length of parameres ca 0.5 mm, of basal piece ca 0.6 mm, of endophallic tooth-field ca 0.6 mm. Length of longest endophallic teeth 56–58 μm.

##### Etymology.

This species is named after Dr F.-T. Krell of the Museum of Nature and Science, Denver, Colorado.

##### Remarks.

*Aphodiuskrelli* sp. nov. is endemic to Sardinia (Fig. 29a). Pittino (2010) lists various Sardinian localities, including Badde Salighes, but all the analysed material is from Badde Salighes (Fig. 27d). *Aphodiuskrelli* sp. nov. is described as a new species despite the fact that its karyotype is similar to that of *A.bameuli* sp. nov. from Corsica. In spite of the karyological similarities, it is genetically divergent from *A.bameuli*, from which it differs by 4.7%, and instead clusters with *A.bellumgerens* sp. nov., forming a strongly supported clade. In addition the morphometric study clusters *A.krelli* sp. nov. between the *niger*-crown group and the 3B group, from which it differs in being significantly more convex. Morphologically it differs from *A.bameuli* sp. nov. in being generally more heavily sculptured and more rounded. Its habitat is also different – whereas *A.bameuli* sp. nov. is a dung-inhabiting species, *A.krelli* sp. nov., like most *Liothorax*, is found at the edge of water. The type material, brought home alive for laboratory studies, was divided into two lots. One was given organic detritus from where it was collected while the other was given damp fibrous cow-dung of the sort eaten by *A.bameuli* sp. nov. The beetles given detritus continued feeding on the journey home but those given dung did not feed at all.

#### ﻿ Aphodius (Liothorax) isikdagensis

Taxon classificationAnimaliaColeopteraScarabaeidae

(Balthasar, 1952)

90E91BD0-8620-5953-91E3-513E8E367030

Figs 3c, d , 6a, b , 16e , 18s , 24c , 26c ’

Aphodius (Ataeniomorphus) isikdagensis Balthasar, 1952: 22.

##### Type material studied.

***Holotype*** (labelled as ♂, undissected), with the locality data as given above and a label “Aphodius (Ataeniomorphus) isikdagensis n. sp., Dr V. Balthasar”. At present *A.isikdagensis* is known only from the type series, the holotype and seven paratypes, one labelled as ♀ allotype, with the locality data Çamlidere, Isik d., Anat. 23.vi.47. Exp. N. Mus. ČSR. ***Paratypes***: 1 labelled as ♀ allotype and 6 paratypes, noted by Balthasar as 4♂♂ and 2♀♀. Thus 1 paratype is unaccounted for. Balthasar does not appear to have dissected any of the types (though a male paratype had been dissected) and apparently distinguished the sexes on the degree of clypeal bulging and the degree to which the genae were angled out at their junction with the clypeus. Both these characters are clear in two of the paratypes (Figs 3c, d, 6a, b) the largest of the ♂♂ and ♀♀, but not in two other ♀♀ where the reduced clypeal bulging is clear, but not the outward angling of the genae. Assuming 1 ♂ paratype is elsewhere, the 6 paratypes noted by Balthasar comprise 2♂♂ and 4♀♀. It may be that Balthasar inadvertently switched the numbers of males and females. Balthasar gives the length range as 4.5–5.5 mm. Our measurements are based on stacked images.

##### Additional material studied.

One male, Turkey, Artvin Çam gec. 2000 m, coll. Ziani, shown by its aedeagus (Fig. 24k) to belong to the *A.niger* section, may belong here. Its length is 4.64 mm, width 1.89 mm, so it is within the size range of *A.isikdagensis*, with which it also agrees in the brown, not blackish, legs, though the maxillary palpi are black. The aedeagus (Fig. 24k) in lateral view is shown to have the parameres only weakly downturned apically.

##### Differential diagnosis.

The larger of the two known Turkish species, identified as a member of the *A.niger* group by the large recurved endophallic teeth (Fig. 24c). Maxillary palpi mid-brown. Epipharynx (Fig. 8c, d) with apophobae arranged in narrow bands ca two bristles wide.

##### Redescription.

General appearance (Fig. 3c, d). Length 4.6–5.8 mm, width 1.9–2.5 mm. Head black, browner towards the anterior and lateral margins, no tubercle on frontoclypeal suture. Frontoclypeal suture fine and straight-transverse over middle 1/3, at either end of this either effaced or angled forward to reach the edge of the head at the junction of the clypeus and genae. Outer margin of genae more or continuous with that of the frons in male, distinctly angled outwards in female (Fig. 6a, b). Clypeus moderately bulging upwards medially in male, weakly so in the female, anterior margin broadly excised medially, the sides of this excision bluntly rounded. Punctation rather weak, sometimes stronger and tending to be rugose towards the anterior and lateral margins. Epipharynx (Fig. 8c, d) with the central tylus strongly projecting and the anterior margin of the clithra clearly excised either side. Central darkened sclerotised epitorma broadly triangular, the whole median darkened region appearing widest at base (Fig. 8c) or narrowed basally (Fig. 8d). Acropariae virtually absent, chaetopedia strong, six or seven each side. Surface of gymnopedia covered with small tooth-like asperities. Prophobae quite strong, clustered at the edges of the sclerotised mesoepitorma, apophobae fine, arranged in a narrow slightly irregular band ca two bristles wide, outside the chaetopariae. Antennae and palpi mid brown. Pronotum (Fig. 6a, b) hemicylindrical, highly arched transversely, scarcely at all longitudinally. Entire lateral margins visible from above. Surface with double punctation, male paratype with the larger punctures separated by at least twice their diameter, sparser on disc. The female paratype has the punctation sparser and finer. Posterior margin completely bordered (Figs 6a, b, 16e), the border wider medially, narrowed but continuous round the posterior corners.

Scutellum pentagonal, elongate, glossy, sometimes with a few punctures medio-basally. Elytra black, interstices flat, 6–8× width of the striae, with fine isodiametric reticulation and sparse fine punctures (Fig. 17k).

Metaventrite (Fig. 21x, y) moderately punctured, flattened over median diamond-shaped plate, sometimes with an impressed median line. Legs mid brown, basal segment of mesotarsi elongate, either clearly longer than the longer tibial spur, or ca the same length (Fig. 18s).

Aedeagus (Fig. 24c): total length ca 1.1 mm, parameres length ca 0.48 mm, basal piece length ca 0.64 mm, length of endophallic tooth-field ca 0.70 mm. Length of longest tooth on endophallus (measured from photograph) ca 60 μm. Parameres with darkened sclerotised strut running from near the outer margin at the base to the inner margin at apex, where the apical sensory pads run round the apex from the tip of the oblique strut to the outer edge, almost to its outer apical angle.

##### Remarks.

This species is identified as a member of the *Aphodiusniger* group by the large, recurved teeth on its endophallus (Fig. 24c), and the parameres with the apical sensory pads running round the apex from the tip of the oblique strut to the outer edge. This may be the result of collapse of only weakly downturned apically parameres as a result of drying.

Dellacasa et al. (2001b) regarded this species as conspecific with *A.rutilipennis*, at that time known as *A.ressli* (Petrovitz, 1962). They figure the aedeagus (without the endophallus in its sac), but the parameres do not really match those of the paratype shown in Fig. 24c. Dellacasa et al. (2007), in their revision of the world species of *Liothorax*, show the same figure of the aedeagus.

At present *A.isikdagensis* is known only from the type series, the holotype and seven paratypes, one labelled as ♀ allotype, with the locality data Çamlidere, Isik d., Anat. 23.vi.47. Exp. N. Mus. ČSR (Fig. 29b, c). Other Turkish material (Vilayet Rize, Ovitdagi gecidi m 2600, coll. Balerio; Kizildag Gecidi 2290 m, coll. Král; Artvin Çam gec. 2000 m, coll. Ziani) is shown by its parameres to belong to the *A.niger* species complex, and this is also true of a male from central Armenia, Selim Pass 2350 m, coll. Ziani. See discussion of *L.alberti* sp. nov.

#### ﻿ Aphodius (Liothorax) alberti sp. nov.

Taxon classificationAnimaliaColeopteraScarabaeidae

3EF5EA58-8BCC-5C07-A903-6A941DDCB1D7

https://zoobank.org/EB4FACD0-EC1A-40A1-9DFB-EB3418C749F6

Figs 3e–g , 5c, d , 6c, d , 8k, l , 18t , 20o , 21n, o , 24e–j 25d, 26b’, c’

##### Type material examined.

***Holotype***: ♂, “Turchia – Vil. Rize, Ovitdagi gecidi mt 2600,18-jun-1992. leg. A. Ballerio.” (NHMUK). ***Paratypes***: 4♂♂, 7♀♀, 17 unsexed, data as holotype. 1♂, 1♀, 1 with mouthparts dissected (NHMUK), 1♂, 2♀♀, 14 unsexed (AB). 5♀♀, data as holotype (JFMC).

##### Other material examined.

Four specimens, unsexed, Ulu Dag b. Bursa As. M. occ. V:1968 Schweiger, MNHG, may belong to this species. A male from central Armenia, Selim Pass 2350 m, coll. Ziani is shown by its parameres to belong to the *A.niger* species complex, and it agrees with *A.alberti* in its black to blackish-brown appendages and its small size, length 3.9 mm. The epipharynx of this specimen has the apophobae less regular but the darkened area is narrowed in its basal 1/4.

##### Differential diagnosis.

*Aphodiusalberti* sp. nov. is the second *A.niger* group species recognised in Turkey, and as such requires comparison with *A.isikdagensis*. The most striking distinctions are the size ranges: in *A.alberti* sp. nov. 4.1–4.9 mm, in *A.isikdagensis* 4.5–5.8 mm and the colour of the appendages: in *L.alberti* sp. nov. the legs are blackish brown to very dark brown (Fig. 3e–g), in *A.isikdagensis* mid brown (Fig. 3c, d). The maxillary palpi are metallic black to very dark blackish brown in *A.alberti* sp. nov. as against mid brown in *A.isikdagensis* (metallic black in the Artvin specimen). In *A.isikdagensis* there is a sexual dimorphism in the head: the males with the central area of the clypeus clearly more strongly bulging upwards than in females, and the outer margin of the genae almost continuous with the clypeal margin in the male, but distinctly angled outwards in the female. In *A.alberti* sp. nov. the central area of the clypeus of both sexes bulges upwards strongly, and the outer margin of the genae is almost continuous with that of the clypeus. The epipharynxes of the two species show some clear differences: in *A.isikdagensis* the median darkened area is broadly triangular, widest at its base, there are six or seven chaetopedia each side, and the rows of apophobae are arranged in narrow bands, ca two bristles wide, while in *A.alberti* sp. nov. the darkened area is narrowed over its basal 1/3, there are four chaetopedia each side and the rows of apophobae are as single lines (Fig. 8c, d, k, l).

##### Description.

General appearance (Fig. 3e–g). Length 4.1–4.5 (♂), 4.1–4.9 mm (♀), width 1.9–1.95 mm (♂), 1.9–2 mm (♀). Head (Figs 3e–g, 6c, d) black, anterior clypeal margin narrowly dark brown, excised medially, angles at sides of excision bluntly rounded. Central area of clypeus bulging upwards in both sexes, punctation fine, moderately dense, with some variation in strength, this not dependant on the sex of the specimen. Frontoclypeal suture fine and straight-transverse over central 1/2, with no trace of a median tubercle, then angled forward to meet the sides just anterior to the genae. Lateral margins of genae either continuous with those of clypeus or with a very slight outward angle, without sexual dimorphism. Maxillary palpi glossy blackish brown, antennae dark brown. Epipharynx (Fig. 8k, l) with the central tylus strongly projecting anteriorly and the anterior margin of the clithra clearly excised either side. Central darkened sclerotised epitorma triangular in apical 2/3, then the sides curved inwards so that the width at the base of the darkened region is ca 3/4 of its maximum width. Acropariae virtually absent, chaetopariae well-spaced, four each side. Surface of gymnopedia covered with small tooth-like asperities. Prophobae fairly strong, clustered at the edges of the median sclerotised mesoepitorma, apophobae fine, arranged in a single line outside the chaetopariae.

Pronotum hemicylindrical, highly arched transversely, scarcely at all longitudinally. Entire lateral margins visible from above. Surface with double punctation, some specimens (male and female) have the punctation heavier with the larger punctures separated by at least twice their diameter, sparser on disc (Figs 3e, g, 6d), in others the punctation is sparser and finer (Figs 3f, 5c, d). Posterior margin completely but narrowly bordered (Figs 3e–g, 6c, d), the border not widened medially, continuous round the posterior corners. Scutellum pentagonal, elongate, glossy, sometimes with a few punctures medio-basally.

Elytra black, interstices flat, 6–8× width of the striae, with fine isodiametric reticulation and sparse fine punctures. Metaventrite (Fig. 21n, o) rather finely punctured in both sexes, flattened over median diamond-shaped plate, sometimes with an impressed median line. Legs dark blackish brown (Fig. 3e–g), basal segment of mesotarsi elongate, slightly longer than the longer tibial spur (Fig. 18t). The legs and metaventrite Fig. 20o appear unnaturally pale brown due to the intense lighting.

Aedeagus (Fig. 24e–j) Aedeagus length ca 1.1 mm, parameres length ca 0.44 mm, basal piece length ca 0.59 mm. Length of tooth-field ca 0.58 mm, length of longest teeth ca 54µm. Parameres in lateral view weakly to moderately downturned apically (Fig. 24h–j). In dorsoventral view sensory area either not visible (Fig. 24g, the holotype), or visible after squashing as a curved band round the paramere apex (Fig. 24e, f, a paratype). The parameres struts are clearly less darkened in the paratype, suggesting that the aedeagus is less fully hardened, which would render it more prone to squashing.

##### Remarks.

Apparently widespread on high ground in Anatolia and possibly extending into Armenia (Fig. 29a). According to A. Ballerio (pers. comm., 16.vi.2023) the *Liothorax* were found on snow patches and in puddles at the sides of the road passing through Ovit pass, a typical alpine prairie (Fig. 28e). They were mixed with fewer Aphodius (Neagolius) ovitensis Pittino & Ballerio, 1994.

#### ﻿ Aphodius (Liothorax) wilsonae

Taxon classificationAnimaliaColeopteraScarabaeidae

Maté & Angus, 2005, stat. rest.

804FB70A-33F6-5170-BB50-0EA7C567EA54

Figs 4g , 5k , 6k, l , 9h, i , 16c,d , 17w , 18o , 21u–w , 23g–i , 24o , 26d ’, e’, 33m-p, 34k, l

Aphodius (Liothorax) wilsonae Maté & Angus, 2005: 329.

##### Type material examined.

***Holotype*** ♂, SP Provincia de Burgos, Balneario de Corconte, 40.031°N, 3.884°W, 26.iv.2001. leg. R.B. Angus (NHMUK). ***Paratypes***: SP: Provincia de Madrid, Manzanares el Real, Embalse de Santillana, 40.720°N, 3.857°W, 1.iv.2003, sieving detritus from edge of water, leg. R.B. Angus & G.I. Aradottir, 1♂, 1♀, 1 unsexed (NHMUK).

##### Other material examined.

SP Provincia de Cantabria, Areños, 43.112°N, 4.724°W, 5.vi.2012.By digging mud under dried-out pool. 1♂. Leg. R.B & E.M. Angus; Provincia de Burgos, S of Balneario de Corconte, strandline detritus. 1.iv.2014. leg. R.B. Angus. 1♂ (NHMUK); Provincia de Álava, Vittoria au Mt Gorbea, 34.634°N, 2.782°W, Juin 1879. 1♀ (MNHG); Provincia de Madrid, Puerto de la Morcuera, 40.828°N, 3.399°W. 15.1v.1997. 2, unsexed (NMP).

P: Monchique, Algarve, 37.317°N, 8.557°W. 6–13.v.1910, leg. K. Jordan. 3♂♂, 1♀ (NHMUK); Serra de Estrela, 40.326°N, 7.708°W. leg. H. Fery. 2♂♂, 1 unsexed (ZSM).

##### Differential diagnosis.

Endophallic teeth conspicuously shorter than in all other species, ca as high as long. Length of longest teeth ca 35 μm (Fig. 23g–i, 24o), as against at least 40 μm in other species.

##### Redescription.

General appearance (Fig. 4g). Length 3.3–4.8 mm (♂), 3.8–5 mm (♀), width 1.6–2.1 mm (♂), 1.7–2.2 mm (♀). Black without obvious paler edges to head, antennae, palpi, and legs black to very dark brown. Head with the frontoclypeal suture very fine, often more or less effaced, laterally almost always so, and without tubercles. Clypeus bulging upwards medially, with double punctation, this finer on disc. Epipharynx (Fig. 9h, i) with anterior margin of clithra strongly emarginate either side of the projecting median tylus, with a few very fine acropariae. Chaetopedia well-developed, stout, 6–8 each side. Chaetopariae shorter and thinner than chaetopedia, forming a closely-set line each side. Apophobae forming a line of very fine hairs outside the chaetopariae, prophobae lying in small inconspicuous groups near the sides of the mesoepitorma.

Head and pronotum (Fig. 6k, l): pronotum highly arched transversely and generally distinctly arched longitudinally, giving a somewhat domed appearance, the hemicylindrical appearance less usual than in other *Liothorax* species. Surface of pronotum bulging outwards laterally so that the lateral margins are sometimes not visible from above in their basal 1/3s. Surface with double punctation, the strength of this variable, from rather fine (Fig. 5k) to distinctly coarser.

Scutellum elongate, pentagonal, glossy with a few punctures medially.

Elytra black, interstices with very fine reticulation (Fig. 17w) and widely separated fine punctures, ca 8× the width of the striae. Striae glossy, vertical-sided, with punctures separated by ca 2× their diameter and encroaching on the vertical sides of the striae (Fig. 16w). Metaventrite (Fig. 21u–w) with central diamond-shaped area bearing sparse fine or moderate punctures and often small patches of reticulation. No obvious sexual dimorphism. Legs dark brown, longer spur of mesotibiae clearly slightly longer than basal segment of mesotarsus (Fig. 18o).

Aedeagus (Figs 23g–i, 24o). Length ca 1.1 mm, paramere length ca 0.43 mm, basal piece length ca 0.69 mm, length of endophallic tooth-field ca 0.37 mm, length of longest teeth ca 35 μm.

##### Remarks.

*Aphodiuswilsonae* stat. rest. is widely distributed mainly on higher ground in the northern half of Spain and Portugal, but with some material from the Algarve (this location needs to be verified by future sampling; Fig. 29a). It is generally taken in the spring and early summer, either in detritus at the edges of water bodies (Fig. 27f), or at the roots of vegetation where pools have dried out. It is sometimes very abundant.

### ﻿Chromosomes

Details of the material from which successful preparations have been obtained are given in Table 1, Relative Chromosome Length in Table 4, and a summary of the major features of the karyotypes in Table 5.

***Aphodiusplagiatus*.** Published information: Maté and Angus (2005). 2n = 18 + Xy. Mitotic chromosomes, arranged as karyotypes, are shown in Fig. 30a, b, male from England (Studland), Giemsa-stained and C-banded *L.p.plagiatus*, and Fig. 30c, *L.p.sinoplagiatus* ssp. nov., male from Tibet (Qinghai, Gangca), Giemsa-stained. Heterochromatin is confined to pericentromeric C-bands. There is a distinct step-decrease in size between autosomes 1 and 2. Autosome pairs 5 and 9 are more of less acrocentric, while the others are more or less metacentric, but pairs 4, 7, and 8 have the short arm prone to being less condensed, suggesting a secondary constriction. The X chromosome is a small subacrocentric and the y a very small (dot-like) submetacentric. Qinghai material matches that from Studland and Hunstanton, but C-banding failed (the only nucleus obtained apparently disappeared as a result of the treatment). None of the chromosomes has a heterochromatic long arm.

*Aphodiusrutilipennis*. Published information: none. 2n = 18 + Xy. Mitotic chromosomes, arranged as a karyotype, are shown Fig. 30d, e, Giemsa-stained and C-banded. Autosomes 1, 3, 5, 7, 8, and the X chromosome have heterochromatic long arms. The y chromosome is a dot-like metacentric.

*Aphodiuskraatzi*. Published information: none. 2n = 20 including the sex chromosomes, + B-chromosomes. Karyotypes have been obtained from only 1 ♀, which means the X chromosome cannot be identified. Fig. 30f, g shows the chromosomes from one nucleus, Giemsa-stained and C-banded, while Fig. 30h, i shows a second nucleus, this time with one B-chromosome. Chromosomes 1–3, 5, and 6 have the shorter arms heterochromatic, chromosome 4 is subacrocentric with a heavy pericentromeric C-band, and chromosomes 8–10 are distinctly shorter than the others, subacrocentric and with only small pericentromeric C-bands. The B-chromosome is entirely heterochromatic.

**Figure 34.**
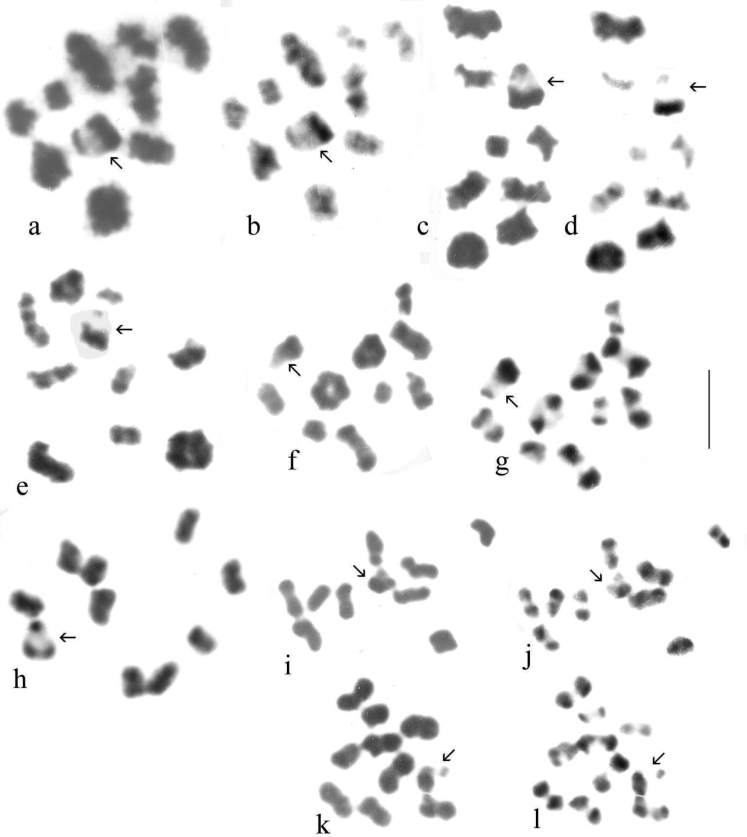
Meiotic chromosomes, metaphase I from testis **a–d***A.niger*, GB, Hampshire, New Forest, White Moor **a**, **c** plain **b**, **d** the same nuclei C-banded **e–g***A.muscorum*, HU, Hortobágyi **e** plain **f**, **g** the same nucleus **f** plain **g** C-banded **h***A.bameuli* paratype, FR, Corsica, by Lac de Melo, plain **i**, **j***A.krelli* IT, Sardinia, paratype **i** plain **j** the same nucleus C-banded **k**, **l***A.wilsonae*, SP, Areños **k** plain **l** the same nucleus C-banded. The sex bivalent is indicated by an arrow. Scale bar: 5 μm.

***Aphodiusniger*.** Published information: Maté and Angus (2005). 2n = 18 + XY_p_ (♂), XX (♀) + B-chromosomes. *Aphodiusniger* differs from all other Aphodiinae whose chromosomes are known in the large, mainly euchromatic (and therefore gene-carrying) Y chromosome. First metaphase of meiosis (Fig. 34a–d) shows that the Y chromosome is strongly condensed and forms a typical “parachute” association with the X chromosome. C-banding (Fig. 34b, d) shows that the C-banding region of the Y chromosome is smaller than the condensed chromosome visible in unbanded preparations (Fig. 34a, c). Mitotic chromosomes, arranged as karyotypes, are shown in Fig. 31a–f, and RCLs are given in Table 2. *Aphodiusniger* is one of four known species in which only one pair of autosomes, in addition to the X chromosome, has a consistently heterochromatic long arm, although the long arm of autosome 3 may be extensively heterochromatic as well (Fig. 30b). The remaining chromosomes have fairly heavy centromeric C-bands. The other species in this group are *A.muscorum* Adam, *A.felix* sp. nov., and *A.bellumgerens* sp. nov. *Aphodiusniger* has autosome pair 2 metacentric, pairs 1 and 3–6 submetacentric and pairs 7–9 subacrocentric. The X chromosome is submetacentric and the Y is metacentric. The B-chromosomes are metacentric and heterochromatic (Fig. 31b, e).

***Aphodiusmuscorum*.** Published information: none. 2n = 18 + Xy_p_ (♂), XX (♀). Mitotic chromosomes, arranged as karyotypes, are shown in Fig. 31f–l, and RCLs are given in Table 2. The y chromosome is a small submetacentric, smaller than autosome pair 9 and largely heterochromatic (Fig. 31g). Metaphase I of meiosis shows the Xy_p_ bivalent with the y far smaller than in *A.niger*, and in one case only revealed after C-banding (Fig. 34e–g). The X chromosome and one pair of autosomes have a heterochromatic long segment, but in this case the autosome, although placed as pair 1, is slightly shorter than pair 2. This arrangement is because pair 2, metacentric and with a heavy but discrete centromeric C-band, appears to be the same as in *A.niger*. Autosome pairs 1 and 3–7 are submetacentric, with pair 8 being borderline submetacentric-subacrocentric, and only pair 9 clearly subacrocentric. The RCL table shows pair 1 to be significantly shorter than in *A.niger* and pair 7 to be significantly longer. The RCL values are clearly the same in the sample from the Hortobágyi and the two females from Kisújszállás, though here the sample size is too small for the larger autosome 7 to appear significantly different from that of *A.niger*. The extent of the heterochromatin on the long arm of autosome 1 shows some variation (Fig. 31) but the heterochromatic region is not sufficient to account for the smaller size of this autosome than that of *A.niger*.

***Aphodiusfelix* sp. nov.** Published information: none. 2n = 18 + Xy (♂), XX (♀). Mitotic chromosomes, arranged as karyotypes, are shown in Fig. 32a–e, and RCLs are given in Table 2. No preparation of meiosis is available. Autosome 1 and the X chromosome have the long arm heterochromatic and the long arms of autosome pairs 4, 5, 6, and 8 may also be heterochromatic, with the heterochromatin staining less densely than that of the centromeres. Autosome 8 is polymorphic for a pericentric inversion and may be either metacentric or subacrocentric. Autosome pairs 2 and 3 are metacentric, 4 – 7 and the X chromosome are submetacentric, and pair 9 is subacrocentric. The y chromosome is a very small, almost dot-like, metacentric. Autosomes 1 and 2 are ca the same size and pairs 3–9 show a progressive decrease. The X chromosome is ca the same size as pair 6.

***Aphodiusbellumgerens* sp. nov.** Published information: none. 2n = 18 + Xy (♂), XX (♀). Mitotic chromosomes, arranged as karyotypes, are shown in Fig. 32f–i, and RCLs are given in Table 2. No preparation of meiosis is available. Autosome 1 and the X chromosome have their long arms clearly heterochromatic, and the X chromosome is the longest in the nucleus. Autosomes 1–5, 7, and 8 are submetacentric, and 5 and 9 are subacrocentric. The y chromosome is a small submetacentric, but not dot-like and in fact only slightly shorter than pair 9. It is largely heterochromatic (Fig. 32g).

***Aphodiusbameuli* sp. nov.** Published information: none. 2n = 18 + Xy_p_ (♂), XX (♀). Mitotic chromosomes, arranged as karyotypes, are shown in Fig. 33a–d, metaphase I of meiosis is shown in Fig. 34h and RCLs are given in Table 2. Autosome pairs 1, 2, 6–8, and the X chromosome are submetacentric, with heterochromatic long arms. Autosome 2 is metacentric, with a heavy centromeric C-band, autosome 8 is submetacentric with the centromeric C-band sometimes extending on to the basal part of the long arm, and autosome 9 has a heavy centromeric C-band and is polymorphic for a pericentric inversion and may be either metacentric or subacrocentric. The X chromosome is the longest in the nucleus and the y chromosome is a small subacrocentric, ca 1/3 of the length of autosome 9.

***Aphodiuskrelli* sp. nov.** Published information: none. 2n = 18 + Xy_p_ (♂), XX (♀). Mitotic chromosomes, arranged as karyotypes, are shown in Fig. 33e–l, metaphase I of meiosis is shown in Fig. 34i (Giemsa-stained), j (the same nucleus, C-banded), and RCLs are given in Table 2. The RCLs of the chromosomes, as well as their configurations and distribution of heterochromatin show almost no differences from the karyotype of *L.bameuli*, including the inversion polymorphism of autosome pair 9. Autosome pair 7 has the long arm heterochromatic and is polymorphic for a deletion of its heterochromatic long (Fig. 33k, l). This polymorphism has not been detected in *A.bameuli* and the arm concerned has not been found to be heterochromatic.

***Aphodiuswilsonae*.** Published information: Maté and Angus (2005). 2n = 18 + XY_p_ (♂), XX (♀) + B-chromosomes. Mitotic chromosomes, arranged as karyotypes, are shown in Fig. 33m–p, metaphase I of meiosis is shown in Fig. 34k (plain), l (the same nucleus, C-banded), and RCLs are given in Table 2. Autosome pair 2 is metacentric with a heavy centromeric C-band autosome 9 is submetacentric with a heavy centromeric C-band. The remaining autosomes, and the X chromosome, are submetacentric, sometimes almost subacrocentric, with heterochromatic long arms. The X chromosome is of the same size range as pairs 2–5, clearly smaller than those of *A.bameuli* sp. nov. and *A.krelli* sp. nov. (Table 2). The y chromosome is a very small metacentric, less than 1/2 the length of autosome 9. The B-chromosome, figured by Maté and Angus (2005), is a small submetacentric, similar in size to autosome 9. It has not been C-banded.

### ﻿Molecular analysis

**Figure 35.**
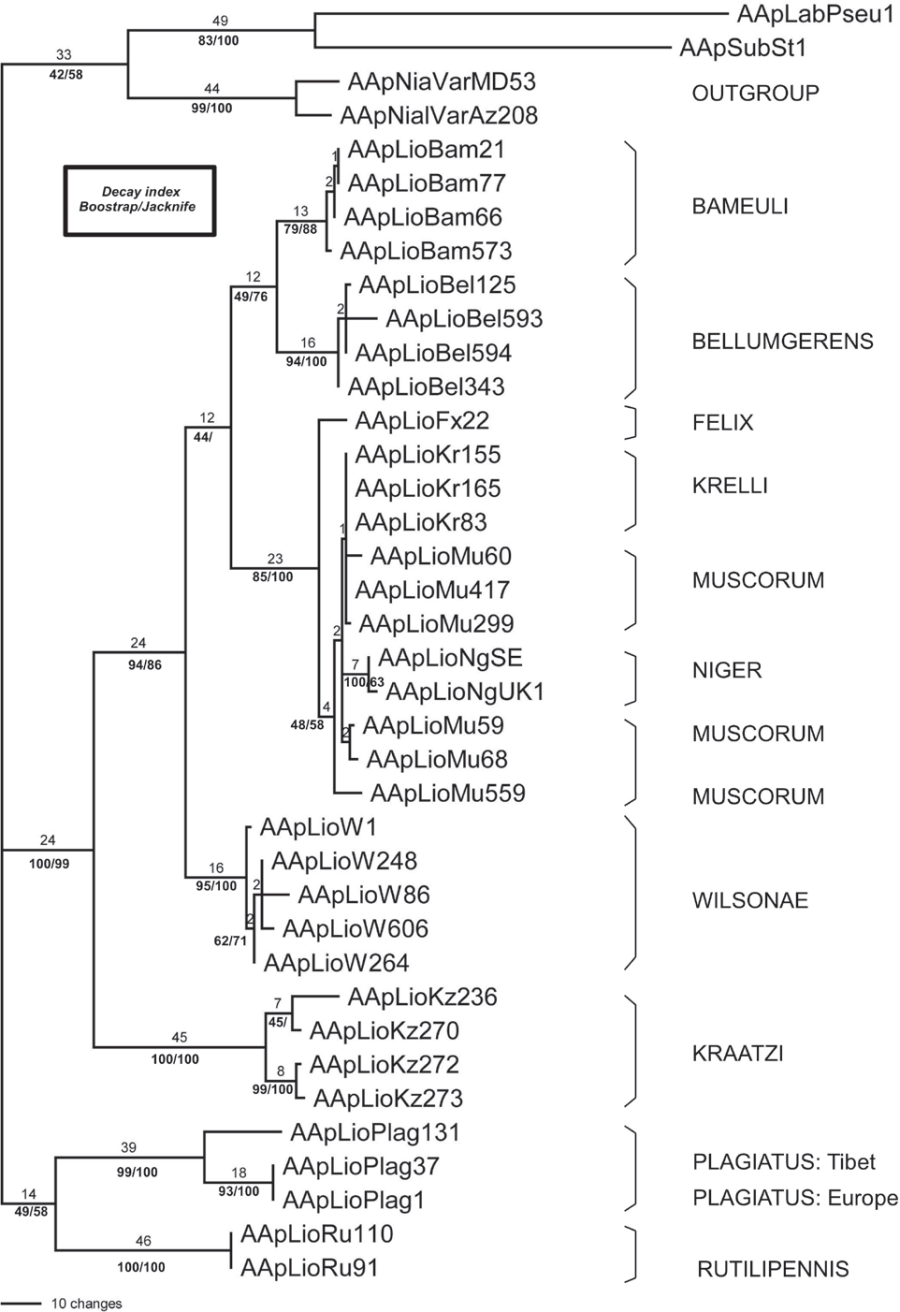
Molecular Majority consensus (80%) phylogeny of 28 equally most parsimonious trees derived from the exhaustive search on the combined dataset of mtDNA (cox1 and Cyt-B). Support values shown only for the supraspecific nodes decay indices above the nodes, bootstraps and jackknife values below them. Scale bar size equivalent to 10 base pair changes.

Thirty-eight specimens were sequenced (Table 4): ten ingroup species (34 specimens in total) and three outgroup species (four specimens) representing three of the assumed most closely related subgenera (*Labarrus* Mulsant & Rey, 1870, *Subrinus* Harold, 1870 and *Nialus*, sensu Rakovič, 1981). Of the 34 ingroup specimens 29 were sequenced successfully for both genes, whilst five *Liothorax* specimens (*A.bameuli* sp. nov. (573), *A.kraatzi* (272 and 273), *A.muscorum* (417), and *A.plagiatus* (1)) failed to produce usable cytochrome-b (cyt-b) sequences despite repeated attempts. Once aligned the sequence ends were trimmed to eliminate primer binding sites and reduce missing data, resulting in a combined dataset 1210 base pairs (bp) long (430 bp for cytochrome b and 780 bp for cytochrome oxidase I), of which 380 bp were variable and 277 bp parsimony informative. The parsimony search found 28 equal length most parsimonious trees (length = 220). The consensus tree (Fig. 35, majority consensus 80%) was well resolved with high consistency (CI, 0.623 [0.568]) and retention indices (RI, 0.842 [0.842]; reported numbers are for all sites first and parsimony-informative sites only second). Node support for most of the species’ groups was strong on all three measures (support is reported for the main nodes only, with decay indices above and bootstraps and jackknife values below, in that order). Within the *niger* clade (*niger* crown+*bameuli*-*bellumgerens*+ *wilsonae*), the data strongly supported all the species outside the *niger*-crown clade (*A.niger*, *A.felix* sp. nov., *A.muscorum*, and *A.krelli* sp. nov.) which was largely unresolved, due to the placement of some specimens of *A.muscorum*, although there was support for the split between *A.felix* sp. nov. and the other three taxa. Support for the *niger*-crown group was high. The maximum likelihood tree search (log-likelihood -1497.25, tree not shown) resulted in a similar topology albeit with some minor differences in the *niger*-crown clade. The *niger* clade was highly supported and all three groups within it showed deep and well supported splits. The *plagiatus* group (*A.kraatzi*, *A.plagiatus* and *A.rutilipennis*) was paraphyletic, with *A.plagiatus*+*A.rutilipennis* forming a clade basal to all the other *Liothorax* species. Within it, *A.plagiatus* and *A.rutilipennis* were deeply split, as were the Asian (Qinghai, specimen 131, subspecies *A.p.sinoplagiatus*) and European *A.plagiatus* species (1 and 37). Similarly *A.kraatzi* had a deep split between the Western European (236 and 270) and Eastern Russian (272 and 273) *A.kraatzi*.

**Figure 36.**
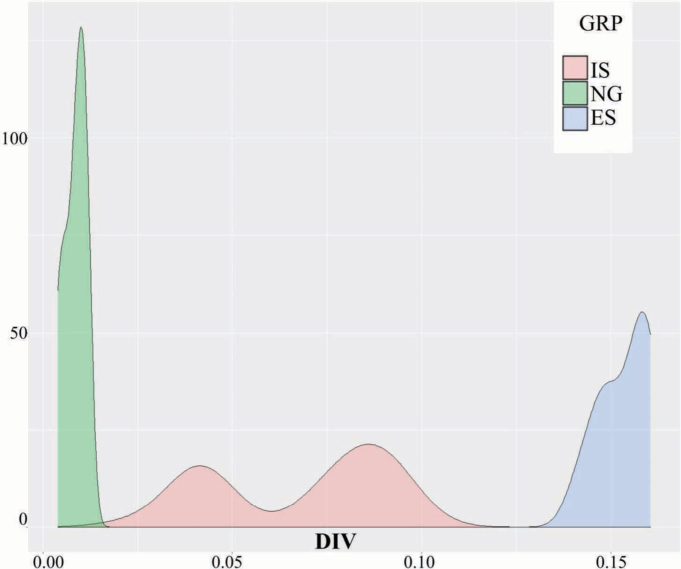
Intraspecific distance comparison of intraspecific (IS), interspecific (ES) and within niger-crown group (NG) distances. The distance distribution showed some overlap between the IS and NG distribution at the extreme but none with the ES distribution both the Wilcoxon’s rank test (< 2e-16) and Kruskal-Wallis (< 0.000001) were highly significant for the latter.

Molecular divergences. The interspecific p-distances are shown in Table 7 and range from > 17% (K2P) between subgenera to 0% within species. These distances are comparable to other studies and congruent with the often-quoted interspecific gap of 2–4%. These distances were further analysed by comparing the distributions of the intraspecific (IS), interspecific (ES) and within *niger*-crown group (NG) distances, as shown in Fig. 36. The distance distributions for all three groups showed little overlap, with the intraspecific and interspecific distance distributions being highly significantly different (Wilcoxon’s rank test [< 2e-16], Kruskal-Wallis [< 0.000001]), whereas the NG and IS distance distributions overlapped slightly more (Wilcoxon [0.0073], Kruskal-Wallis [n.s.]). Much of the overlap within the IS-ES and NG-IS was due to two species: *A.plagiatus*, where there is a very deep split between the Qinghai material (subspecies sinoplagiatus) and the European material (p-distance 0.03203); and *A.felix* sp. nov. with the other *niger*-crown group species, from which is has a greater divergence than the average intragroup divergence (0.01364 vs 0.00525) and intermediate between the highest IS distances and lowest ES distances (East – West *A.kraatzi* [0.02696] or *A.bameuli* sp. nov. and *A.bellumgerens* sp. nov. [0.02731]).

**Table 7.** Intergroup (below diagonal), (diagonal, grey) p-distances and Kimura two-parameter (above diagonal) intra group distances for combined molecular dataset.

	OUTGROUP	A. (L.) bameuli	A. (L.) bellumgerens	A. (L.) felix	A. (L.) krelli	A. (L.) kraatzi	A. (L.) muscorum	A. (L.) niger	A. (L.) plagiatus	A. (L.) rutilipennis	A. (L.) wilsonae
**OUTGROUP**	0.1353	0.1592	0.1647	0.1733	0.1697	0.1604	0.1707	0.1730	0.1571	0.1508	0.1608
**A. (L.) bameuli**	0.1279	0.0038	0.0287	0.0509	0.0466	0.1026	0.0471	0.0525	0.1064	0.0908	0.0453
**A. (L.) bellumgerens**	0.1315	0.0273	0.0051	0.0563	0.0500	0.0970	0.0508	0.0528	0.1089	0.0871	0.0413
**A. (L.) felix**	0.1367	0.0466	0.0513	n/c	0.0119	0.1059	0.0142	0.0147	0.1162	0.0911	0.0521
**A. (L.) krelli**	0.1347	0.0430	0.0460	0.0116	0.00	0.1051	0.0040	0.0077	0.1161	0.0958	0.0463
**A. (L.) kraatzi**	0.1293	0.0885	0.0843	0.0905	0.0901	0.0209	0.1060	0.1047	0.1163	0.1080	0.0963
**A. (L.) muscorum**	0.1351	0.0435	0.0467	0.0138	0.0039	0.0909	0.0057	0.0101	0.1161	0.0987	0.0478
**A. (L.) niger**	0.1367	0.0480	0.0484	0.0143	0.0076	0.0898	0.0099	0.0017	0.1186	0.0985	0.0491
**A. (L.) plagiatus**	0.1262	0.0901	0.0921	0.0966	0.0967	0.0983	0.0968	0.0984	0.0203	0.0953	0.1036
**A. (L.) rutilipennis**	0.1227	0.0791	0.0764	0.0793	0.0830	0.0925	0.0851	0.0850	0.0824	0.00	0.08696
**A. (L.) wilsonae**	0.1289	0.0419	0.0386	0.0476	0.0429	0.0837	0.0442	0.0452	0.0878	0.0762	0.0047

The results are congruent with and supportive of the specific status of several undescribed species. Within the *niger* group, we find strong molecular support for the species status of *A.wilsonae* as concluded by Maté and Angus (2005). At the time they noted that though the sampling was limited, the divergence between the three available species (*A.niger*, *A.plagiatus*, and *A.wilsonae*) was large enough to assume that all three were good species. In the present study we have a much larger the sample size in addition to a wider geographical area, which further supports the original hypotheses. Furthermore, the empirically derived divergence range (3.3–4.2%) is well above the commonly used cutoff 2–2.5% and far greater than the interspecific distances (< 1%) within *A.niger* or *A.wilsonae* (Table 5), and between *A.wilsonae* and any of the other *niger*-group species. This leads us to the conclusion that *A.wilsonae* is a *bona species* according to the molecular markers and reject Dellacasa and Dellacasa’s (2005) synonymisation. Regarding the rest of the *niger*-group clade we also find strong molecular support for the specific status of *A.bameuli* sp. nov. and *A.bellumgerens* sp. nov., with distances > 4% between these taxa and others within the *niger* sensu lato group. The remaining species, conforming the *niger*-crown clade, have poor resolution and do not coalesce into monophyletic groups due to *A.muscorum* and *A.krelli* sp. nov. There are several reasons why this could be the case, such as incomplete lineage sorting or introgression, but considering the low divergences, it is likely that these lineages represent a very recent speciation event. Additional sampling and the sequencing of faster evolving nuclear genes will be necessary to elucidate their relationships.

The results for the sequenced *plagiatus* group species support their specific status but they do not form a monophyletic clade. Instead they are a paraphyletic group, with *A.rutilipennis+A.plagiatus* as the most basal sampled species and *A.kraatzi* as sister to the *niger* group. Furthermore, the deep divergence within *A.plagiatus* between the Chinese and European samples, is suggestive of a cryptic species complex. Though the sample size is insufficient to consider raising the Chinese populations to specific status, the molecular evidence, in conjunction with certain morphological traits such as the distinctly larger aedeagi and the spermathecae of the Chinese material, justifies recognition of a distinct subspecies inhabiting the Tibetan plateau.

### ﻿Morphological analysis

#### ﻿Principal component analysis

The data were found to be suitable for PCA (MSA = 0.95, Bartlett’s K-squared spherecity test = 174.32 (p ≤ 0.001)) and the minimum number of factors to extract was determined to be two according, explaining over 80% of the variability.

Factor rotation under Varimax resulted in higher variance explained (cumulative loading 82%) but under Promax better separation was achieved (cumulative loading 67%). In either case factor loadings split along the same morphological features, with leg characters being positively loaded on factor 1 (PC1) and body measures (pronotal length and width, scutellar length and body height) on factor 2 (PC2). The other body measures (elytral length and width) were equally loaded on both components.

**Figure 37.**
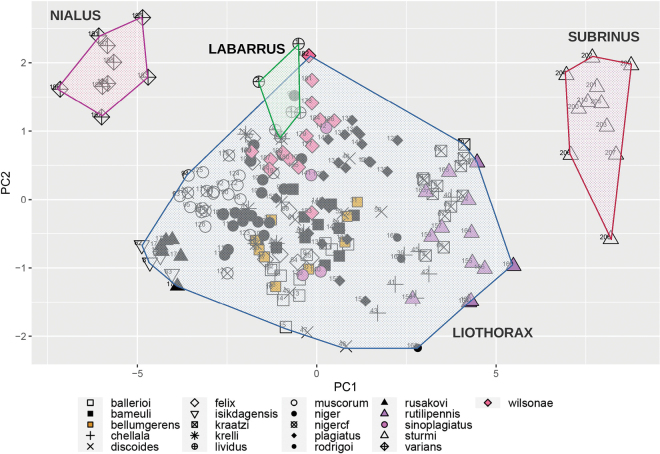
Bivariate plot of the principal components 1 and 2 derived from the linear morphological measurements. Subgeneric groupings indicated by the polygons enclosing the taxa, with vertices in the outermost specimens of each group.

The resulting values clearly distinguished several groups. The generic groupings were highly divergent (Fig. 37) except for A. (Labarrus) which was not significantly different from *Liothorax*.

Because of this large morphological divergence between the genera, the dataset was reanalysed after excluding the outgroups A. (Nialus) and A. (Subrinus) to determine if the PCA values and loadings were overwhelmed by the subgeneric differences rather than the specific ones. The results of the analysis of the culled dataset were broadly similar to the complete dataset and cumulative variance explained remained almost unchanged (70% under Varimax and 66% under Promax, same loadings), indicating that the divergences and loadings were comparable, and henceforth all analyses were done on the full dataset.

**Figure 38.**
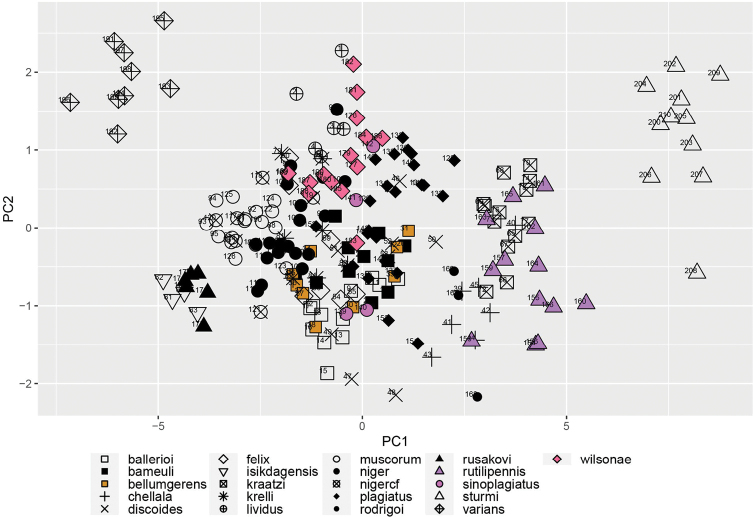
Bivariate plot of the principal components 1 and 2 showing the distribution of all the species in the morphospace as defined by the measurements taken in this study. For the translation of the symbols refer to the legend below the figure.

**Figure 39.**
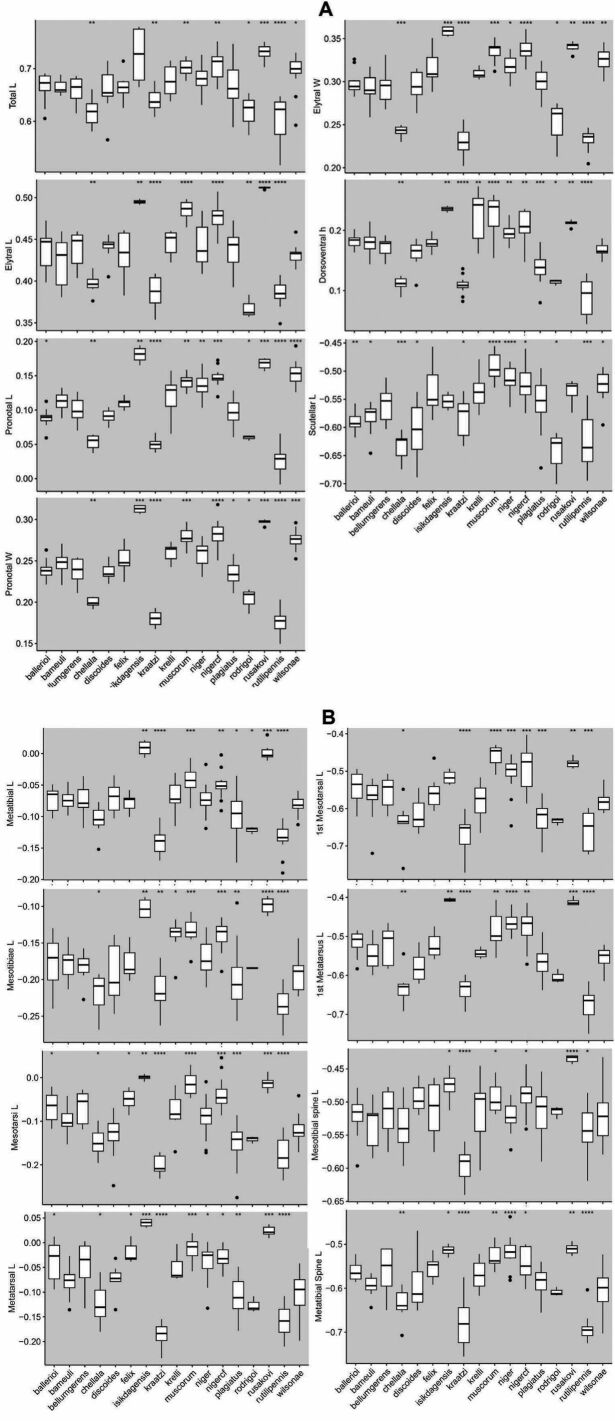
Boxplots of the allometrically corrected linear measurements for body measurements **A** and leg measurements **B** used in the PCA. Species names in the x axes and morphological features in the y axes.

Visual examination of the principal component bivariate plot (Fig. 38) and the boxplots (Fig. 39) demonstrated distinct groupings for several taxa. Within the *niger* group taxa (*A.niger*, *A.muscorum*, *A.krelli* sp. nov., *A.felix* sp. nov., *A.wilsonae*, *A.bameuli* sp. nov., and *A.bellumgerens* sp. nov.), the divergence of *A.wilsonae* from the other species is obvious (Fig. 36) and comparable to the position of the outgroup taxon *Labarrus*, occupying an intermediate place between the *niger*-group and *A.plagiatus*. The second major split concerned the species in the *niger*-crown group and the *bameuli*-*bellumgerens-alberti* group (3B group), with barely any overlap except for some *A.krelli* sp. nov. and *A.felix* sp. nov. specimens. Within this group, *L.bameuli* sp. nov. and *L.bellumgerens* sp. nov. broadly overlap, whereas *A.alberti* sp. nov. diverges from them along PC2 (shorter scutellum). Within the *niger* crown group (*A.niger*, *A.muscorum*, *A.krelli* sp. nov., and *A.felix* sp. nov.), *A.niger*, and *A.muscorum* form two groupings with partial overlap where the *A.niger* cf. specimens are placed (material from Czechia and western Russia which could not be karyotyped as *A.muscorum* or *A.niger*), whereas *A.krelli* sp. nov. and *A.felix* sp. nov. broadly overlap with each other.

Within the *plagiatus* group, *A.plagiatus* sensu stricto was clearly separated from the rest of the species in the group, with no overlap at all with any species other than *A.discoides*. The other species of the group dispersed widely. There was a partial overlap between *A.rutilipennis*, *A.chellala* sp. nov. and *A.rodrigoi* sp. nov., but it was only due to three very divergent specimens (one from each species) which overlapped, while the majority clustered with conspecific specimens exclusively. Neither *A.discoides* nor *A.isikdagensis* overlapped at all with *A.rutilipennis* (as would have been expected according to the synonymy with *A.rutilipennis* sensu Dellacasa et al. (2007)). *Aphodiusisikdagensis* formed a tight cluster next to *A.rusakovi* and well away from the other *Liothorax* but ultimately closer to the *A.niger* clade (as would be expected from the endophallus of *A.isikdagensis* but not *A.rusakovi*), whilst *discoides* was closest to the group of East Asian *A.plagiatus*. Morphologically it also showed a very spread-out distribution considering the relatively small sample size (8 specimens). Finally, *A.kraatzi* was recovered away from most of the other *plagiatus*-group species, overlapping *A.rutilipennis*.

### ﻿Statistical analyses of individual characters

Morphological divergences between species pairs were highly significant on many characters, congruent with specific-level differentiation of the hypothesised species. All the characters demonstrated highly significant divergences in aggregate (p < 0.001, Wilcoxon’s test and Tukey’s test) and in most species-pair comparisons, with body proportions (width, length, and height) being the most significantly divergent, followed by the tibial and tarsal lengths. The results are summarised in a colour-coded table (Fig. 41), indicating the number of significant morphological differences in each species-pair comparison.

Within the *niger* clade, *A.wilsonae* was significantly divergent from all species in the *niger* group. *Aphodiuswilsonae* differs from other *niger*-group species in having shorter meso- and metatarsi, 1^st^ meso- and metatarsal segments, and a shorter mesotibial spine (Fig. 39B). These differences in the hind legs had previously been noted by Maté and Angus (2005). However, we can also report that *A.wilsonae* differs from the *niger* crown in being overall flatter and with shorter hind tibiae. From *plagiatus* it differed in having a significantly larger pronotum and being overall wider (Fig. 39A). The species in the *niger*-crown group (*A.niger*, *A.muscorum*, *A.krelli* sp. nov., and *A.felix* sp. nov.) were significantly to very significantly divergent in several morphological features compared to each other except for *A.krelli* sp. nov. *Aphodiusfelix* sp. nov. was significantly wider, with a wider and shorter pronotum (Fig. 39A), shorter legs and more flattened form than the other species, whereas *A.muscorum* had a longer and wider body (elytral length and width) and longer midlegs, particularly compared to *A.niger* sensu auctorum. *Aphodiuskrelli* sp. nov. occupied a middling position amongst the previous three species, differing the least from *A.felix* sp. nov. in its dorsoventral height and strongly from *A.niger* (shorter first metatarsal and first mesotarsal lengths and mesotibial length) and from *A.muscorum* (shorter first metatarsal and first mesotarsal lengths, narrower elytral width). The *niger*-crown group and the *bameuli*-*bellumgerens-alberti* group (3B group) differed significantly in several characters, namely the relative size of the pronotum (significantly smaller in the 3B group) and the shorter scutellum and more flattened shape (Fig. 39A) and the shorter middle legs. The closest species from the *niger* crown group to the 3B group was *A.felix* sp. nov., which was significantly wider. Within the 3B group there were no significant morphological differences. Both *A.rusakovi* and *A.isikdagensis* were significantly different from all other species morphometrically. They had significantly larger bodies (Fig. 39A) and longer legs (Fig. 39B), and were placed closest to *A.muscorum*, from which they differed in their longer hindlegs.

In the *plagiatus* group, one of the major divergences was between *A.plagiatus* and most of the other species in the group (*A.rodrigoi* sp. nov., *A.chellala* sp. nov., and *A.rutilipennis*) in numerous characters. *Aphodiusplagiatus* had a larger pronotum, was overall wider and more convex and had a longer scutellum (Fig. 39A). It was similar in gross morphology to *A.discoides*, from which it differed in being significantly more convex, and having a longer scutellum and shorter meso- and metatarsi. As for the other three species, *A.rodrigoi* sp. nov., *A.chellala* sp. nov., and *A.rutilipennis*, they were quite similar morphologically to each other and the differences amongst them were mostly not significant, the only exception being *A.chellala* sp. nov. which had a significantly bulkier prothorax. Finally, *A.kraatzi* had a highly divergent morphology compared to all other *Liothorax* and was closest to *A.rodrigoi* sp. nov. and *A.rutilipennis*, but still differing significantly in the relative length of the mesotibial spine and the remarkably shortened pronotal length.

**Figure 40.**
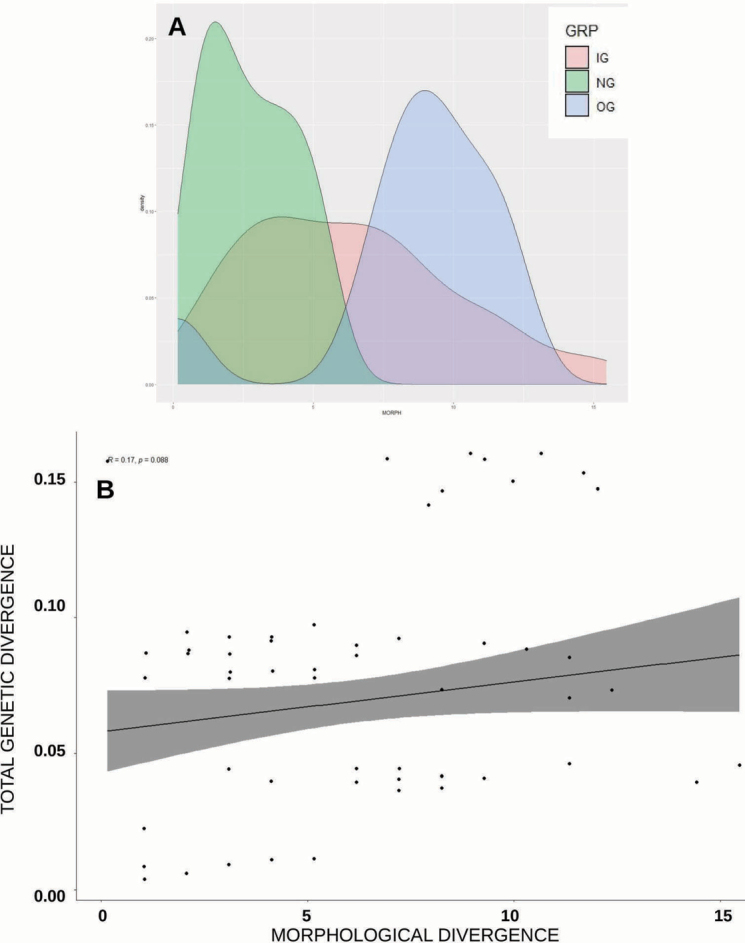
Comparison of the morphological distributions **A** at three phylogenetic levels: within the *niger*-crown group (NG), between species (IG) and between the genera/subgenera (OG)) **B** correlation between morphological and molecular divergence, demonstrating weak and non-significant positive correlation between both data sources.

Comparison of the morphological divergence at the three levels (Fig. 40A; within the *niger*-crown group, between species and between genera) shows a similar but non-significant result to the molecular divergences (Fig. 35). Hence, though the divergence values correlate with the phylogenetic distance, they overlap so broadly that they are non-significant. The lack of significance was driven mostly by the species in the 3B clade (which morphologically overlapped widely with each other as well as with some of the other *niger* group specimens) as well as the subgenus Labarrus (wide overlap with *A.wilsonae*). This is particularly obvious with the datapoints in the lower right quadrant in the bivariate plot (Fig. 40B) which corresponded to specimens in the 3B clade (*A.bameuli* sp. nov. and *A.bellumgerens* sp. nov.) which overlapped broadly both with the *niger*-crown group and *A.plagiatus*, as well as due to a single extreme specimen of *A.wilsonae* (specimen 183) which was morphologically divergent and placed just within the 3B group.

**Figure 41.**
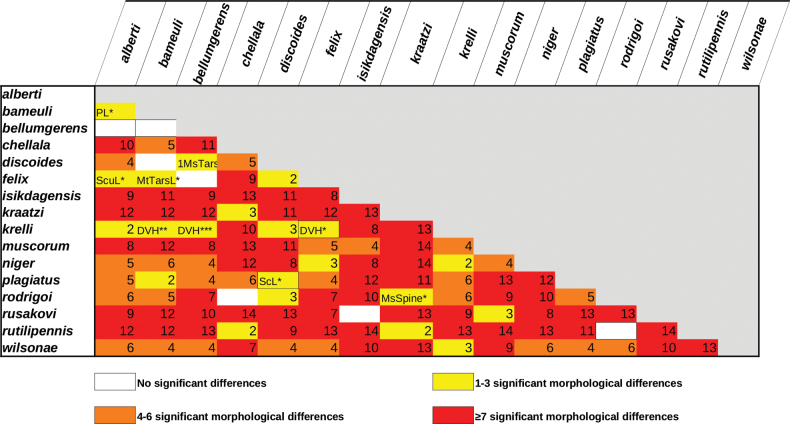
Comparative matrix summarizing the number of significant differences in each species pair. Significance values were corrected to account for multiple comparisons (Bonferroni correction). When there is only one significant difference the level is shown (* <0.01 ** <0.001 ***<0.0001).

## ﻿Discussion

Although *Liothorax* comprises a group of morphologically similar species, this study has found that it is possible to morphologically discriminate many of the species using continuous linear measurements of external, sex-independent morphological characters. Furthermore, these differences were statistically significant and congruent with other independent sources of evidence which support their species status.

The karyological and molecular results of this and previous papers have shown that within the western Palaearctic *Liothorax* there is an unrecognised diversity of species that have remained hidden due to their morphological similarity. However, this similarity is only an artefact of the character suites that have been used to date to identify species in the Aphodiini, as well as a failure to properly account for the significance of what Dellacasa and Dellacasa (2005) regard as “intraspecific variation”, which the authors fail to define, as opposed to interspecific variation. In order to determine what this variation means, it is necessary to quantify it, not just intra- and interspecifically with as wide a geographical coverage as possible, but also within the context of the closest clades (genera/subgenera). This is also necessary on account of the need to accurately identify the cryptic diversity uncovered by the cytogenetic and molecular methods. Unfortunately the Aphodiinae are a morphologically conservative group, particularly compared to the better studied sister clade the Scarabaeinae, which has made their identification and classification difficult and heavily reliant on a small suite of characters, chiefly the aedeagus and epipharynx. Although there are some studies that have used morphometrics to study variation in epipharynx in Scarabaeidae (Pizzo et al. 2009; Tocco et al. 2011), this has been only amongst species pairs. Also, these studies have worked on the same limited data sets so there is a restrictive circularity in the classification of the group.

Within the *niger*-crown clade (*A.niger*, *A.muscorum*, *A.krelli* sp. nov., and *A.felix* sp. nov.), we have found support for the specific rank of these taxa. *Aphodiusniger*, *A.wilsonae*, and *A.muscorum* have been found to be morphologically distinct from each other in this study, a result that is counter to the conclusions of Dellacasa and Dellacasa (2005) and Dellacasa et al. (2007). There is also strong and statistically significant support for the morphological distinctiveness of the *bameuli*-*bellumgerens*-*alberti* clade from the rest of the *niger*-clade taxa, although within the clade we could not find any morphological discriminant features. There was a broad overlap between *A.bameuli* sp. nov. and *A.bellumgerens* sp. nov., and although *A.alberti* sp. nov. diverged noticeably from the other two, this divergence was not significant. Similarly, within the *niger*-crown clade, it was difficult to distinguish *A.felix* sp. nov. and *A.krelli* sp. nov., probably due to noisy data (outlier specimen of *felix* along PC2 overlapped broadly with *krelli*) and insufficient sampling to overcome it. Future studies will address the lack of resolution by increasing the sampling size, which was rather low for these two otherwise easily distinguishable species. Similarly, larger samples of *niger* and *muscorum* should also improve the delimitation of these species and avoid the complications resulting from the indeterminate *niger* cf. material from peripheral areas. Fortunately, *A.bameuli* sp. nov., *A.bellumgerens* sp. nov., and *A.felix* sp. nov. show clear chromosomal differences.

The previously synonymised species *A.isikdagensis*, *A.discoides*, and *A.rutilipennis* have been found to be too morphologically divergent to be conspecific, contrary to previous studies on the group (Stebnicka 1990; Dellacasa et al. 2007). *Aphodiusisikdagensis* (a member of the *A.niger* group, misidentified and wrongly synonymised with *A.rutilipennis*) nevertheless appeared closest to *A.rusakovi* and both were placed next to *A.muscorum*, supporting their inclusion in the *niger* group, as suggested by the aedeagal morphology of *A.isikdagensis*, but not *A.rusakovi*. This morphological affinity, as well as their close geographical distribution, could also be indicative of a close phylogenetic relationship. On the other hand, with the exception of some overlap with *A.discoides*, neither *A.rutilipennis*, *A.chellala* sp. nov., nor *rodrigoi* sp. nov. can be considered conspecific with *A.plagiatus* or *A.isikdagensis*, as their morphology is much too different from either, contradicting their synonymisation by previous authors (Stebnicka 1990; Dellacasa et al. 2001b). However their close morphometric similarity and the small sample size (for *A.rodrigoi* sp. nov. only three specimens were available for all measurements whilst for *A.chellala* sp. nov. only six) limited the statistical power such that they could not be discriminated from *A.rutilipennis*.

In addition, although morphological and molecular divergence were positively correlated, we did not find a significant statistical support for this association, even though the more genetically distant species were morphologically more divergent and vice versa. It is possible that the sample size was too small such that a few outliers overpowered the results. On the other hand, the discriminatory power of the PCA on the morphometric data between closely related taxa (for example between *plagiatus* and the other species in the *plagiatus* group, or between the major clades in the *niger* group), suggests that the gross morphology of the Aphodiini may be under strong selective pressure and not the drift like neutral evolution of mitochondrial molecular sequences. Possible reasons include ecomorphological selection, competitive displacement in sympatry, or a combination of both. A much larger sample of populations is required to answer this question, in particular since there were several species for which no molecular data were available (*A.chellala* sp. nov., *A.discoides*, *A.rodrigoi* sp. nov., *A.rusakovi*, and *A.isikdagensis*), and is beyond the scope of this paper. Similarly, although *A.niger* and *A.muscorum* are well characterised, the undetermined *A.niger* cf. specimens make it difficult to elucidate their exact distributions and to establish to what extent they overlap in Central Europe. Additional positively identified samples (via karyotyping) of these two species from Central and Eastern Europe is needed.

The present multifaceted study, drawing on evidence from morphology, karyology, and genetic analysis shows clearly that the known species of Palaearctic *Liothorax* comprise a monophyletic group of 16 distinct species. As implied by the title of this paper, this number is likely to be increased as more material and data from other populations and species from other areas becomes available. Also requiring clarification is the relationship of the Palaearctic *Liothorax* to those described from the Nearctic.

## Supplementary Material

XML Treatment for Aphodius (Liothorax) plagiatusplagiatus

XML Treatment for Aphodius (Liothorax) plagiatussinoplagiatus

XML Treatment for Aphodius (Liothorax) rodrigoi

XML Treatment for Aphodius (Liothorax) discoides

XML Treatment for Aphodius (Liothorax) rutilipennis

XML Treatment for Aphodius (Liothorax) chellala

XML Treatment for Aphodius (Liothorax) kraatzi

XML Treatment for Aphodius (Liothorax) rusakovi

XML Treatment for Aphodius (Liothorax) niger

XML Treatment for Aphodius (Liothoxax) muscorum

XML Treatment for Aphodius (Liothorax) felix

XML Treatment for Aphodius (Liothorax) bellumgerens

XML Treatment for Aphodius (Liothorax) bameuli

XML Treatment for Aphodius (Liothorax) krelli

XML Treatment for Aphodius (Liothorax) isikdagensis

XML Treatment for Aphodius (Liothorax) alberti

XML Treatment for Aphodius (Liothorax) wilsonae

## ﻿Additional information

### Author ORCIDs

Robert B. Angus https://orcid.org/0000-0002-3860-5617

Jason F. Maté https://orcid.org/0000-0002-2505-7113

David Král https://orcid.org/0000-0002-7930-1927

### Data availability

All of the data that support the findings of this study are available in the main text.
