# ﻿Addenda and corrigenda: Angus RB, Maté JF, Angus EM, Král D (2024) Towards a revision of the Palaearctic species of *Aphodius* Hellwig, 1798, subgenus *Liothorax* Motschulsky, 1860 (Coleoptera, Scarabaeidae, Aphodiinae). ZooKeys 1207: 205–299. https://doi.org/10.3897/zookeys.1207.117225

**DOI:** 10.3897/zookeys.1228.146632

**Published:** 2025-02-21

**Authors:** Robert B. Angus, Jason F. Maté, Elizabeth M. Angus, David Král

**Affiliations:** 1 Department of Life Sciences (Insects), The Natural History Museum, Cromwell Road, London SW7 5BD, UK The Natural History Museum London United Kingdom; 2 c/Henares 16, Velilla de San Antonio, Madrid, 28891, Spain Unaffiliated Madrid Spain; 3 Biomedical Imaging Unit, Level B South Block, Mail point 12, General Hospital, Southampton SO16 6YD, UK Biomedical Imaging Unit Southampton United Kingdom; 4 Department of Zoology, Faculty of Science, Charles University, Viničná 7, CZ-128 00, Praha 2, Czech Republic Charles University Praha Czech Republic

**Keywords:** Corrections, *Liothorax*, new data

## Abstract

Corrigenda to the abovementioned paper are given, as well as new data on Aphodius (Liothorax) felix Angus et al., 2024 and A. (L.) bellumgerens Angus et al., 2024

## ﻿Addenda and corrigenda

### ﻿Addenda

1. *Aphodiusfelix* Angus et al., 2024 and *A.bellumgerens* Angus et al., 2024 in Calabria.

Angus et al. (p. 268) mentioned newly acquired living material from Calabria, collected from the mud of a dried-out pool near Villagio Mancusa (39.107°N, 16.641°E). This material was collected by members of the Balfour-Browne Club (a water-beetle study group) in the course of a meeting in Calabria. Much of this material was moribund but karyotypes were obtained from three females. These are shown in Fig. 1, along with material from Campo Felice for comparison. Although identification of the X chromosomes is difficult when only females are available, the sequence of chromosomal sizes and shapes along the karyotype matches those of *A.felix*, but not those of either *A.bellumgerens* or the Sardinian *A.krelli* Angus et al., 2024, when their karyotypes are used as templates. The collecting site is shown in Fig. 2.

**Figure 1.**
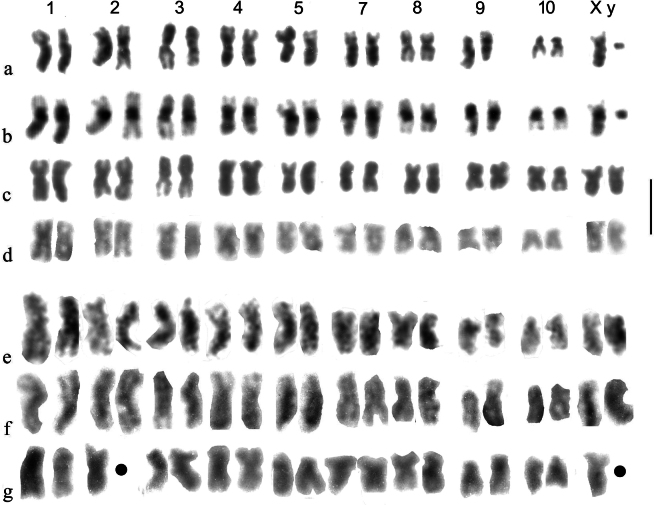
Mitotic chromosomes of *A.felix* Angus et al., 2024, arranged as karyotypes **a**, **b** Campo Felice, ♂♂, **a** Giemsa stained **b** C-banded **c** Campo Felice, ♀, Giemsa stained **d**–**g** ♀♀, Calabria, Giemsa stained **d**, **e** ♀ 2 **f**, **g** ♀4. Missing chromosomes in **g** are indicated by heavy dots. Scale bar: 5 μm.

**Figure 2.**
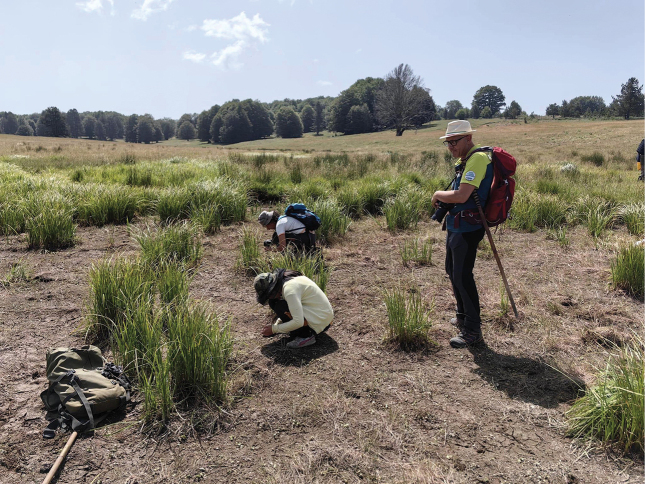
Members of the Balfour-Browne Club working the bottom of a dried-out pool near Villagio Mancusa. Photo courtesy of Zuqi Mai.

The possibility of *A.bellumgerens* occurring in the extreme toe of Calabria was suggested by Jason Maté after studying material borrowed from the Muséum d’histoire naturelle in Geneva, Switzerland.

We have been able to re-examine two of these specimens, a male and a female, collected by Paganetti. Both specimens were in the R. Petrovitz collection. The female has the data “Calabria/ Sta Christina/ lg Paganetti.” and the male simply “Calabria/Paganetti”. The aedeagus of the male was glued to the face of the card on which the beetle was mounted, but only the phallobase remained, the parameres and aedeagal tube having been broken off, probably long ago. However, floating the basal piece off the card and clearing it in KOH solution showed that the retracted endophallus was present, as were the two struts which arise from the base of the aedeagal tube. These parts are shown in Fig. 3, with the whole aedeagi of *A.bellumgerens* and *A.felix* for comparison. The endophallus (Fig. 3c), though contracted, is a better size match for that of *A.bellumgerens* (Fig. 3d) than the larger endophallus of *A.felix* (Fig. 3e).

**Figure 3.**
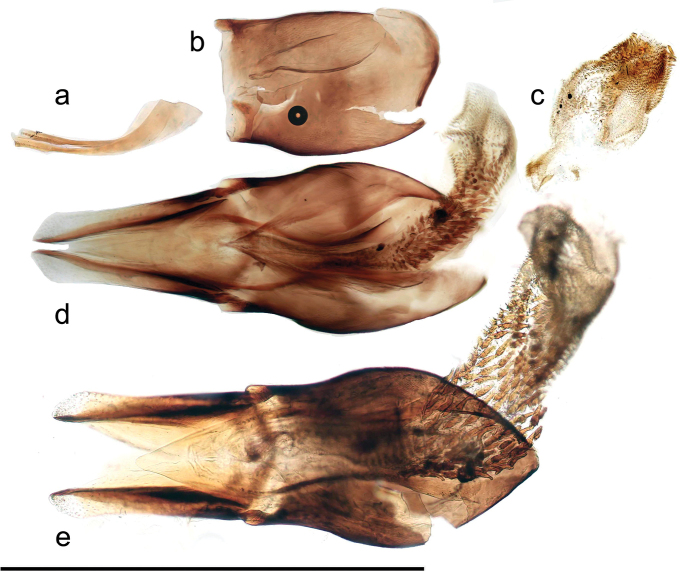
Aedeagi **a**–**c***A.bellumgerens*, aedeagal fragments from a specimen labelled “Calabria, Paganetti” (MHNG) **a** aedeagal struts **b** phallobase **c** retracted endophallus **d***A.bellumgerens*, paratype, IT, Sicily, Nebrodi **e***A felix*, Calabria, Villagio Mancusa. Scale bar: 1 mm.

Any consideration of the beetles from the toe of Italy must address the possibility of Sardinian species being present in view of the close proximity of the Plateau della Sila to Sardinia during the Pliocene. (Foster 2024). This plateau (and its National Park) is adjacent to the Villagio Mancusa locality where the *A.felix* was taken. In the case of subgenus Liothorax, the species involved would be *A.krelli* Angus et al., 2024. Fortunately, the shape of the pronotum distinguishes this species from both *A.bellumgerens* and *A.felix*. In *A.felix* and *A.bellumgerens* the sides of the pronotum bulge outwards so that the lateral margin is to some extent obscured in dorsal view, and the pronotal sides appear somewhat rounded (Fig. 4a–d), whereas in *A.krelli* the pronotal sides are less bulging and the lateral margins are visible throughout, giving straighter sides to the pronotum (Fig. 4e). It thus seems unlikely that *A.krelli* occurs in this part of Italy.

**Figure 4.**
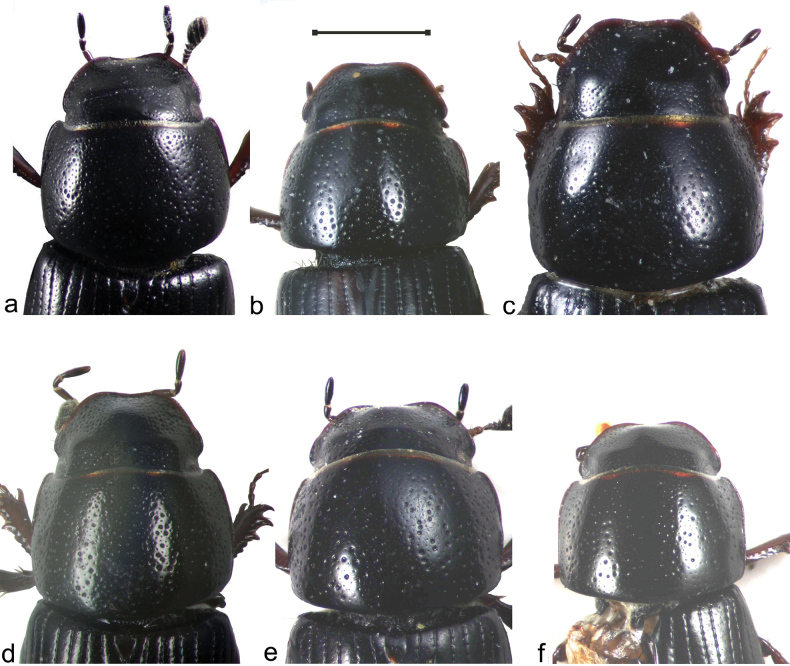
Heads and pronota **a***A.bellumgerens*, holotype, Sicily **b***A.bellumgerens* ♂, Calabria (MNHG)) **c***A.bellumgerens* ♀, Calabria (MNHG) **d***A.felix*, paratype, IT, Campo Felice **e***A.felix* ♀ 2, Calabria **f***A.krelli*, holotype, IT, Sardinia. Scale bar: 1 mm.

Although the identifications of *A.felix* and *bellumgerens* presented here appear to be reliable, it would be highly desirable to get better living material, including males, of both species so that totally unambiguous karyotypes could be obtained.

### ﻿Corrigenda

1. Figs 8, 9. The published figures are from previous manuscript versions and are incorrect and do not match the captions. Corrected versions are given here.

**Figure 8.**
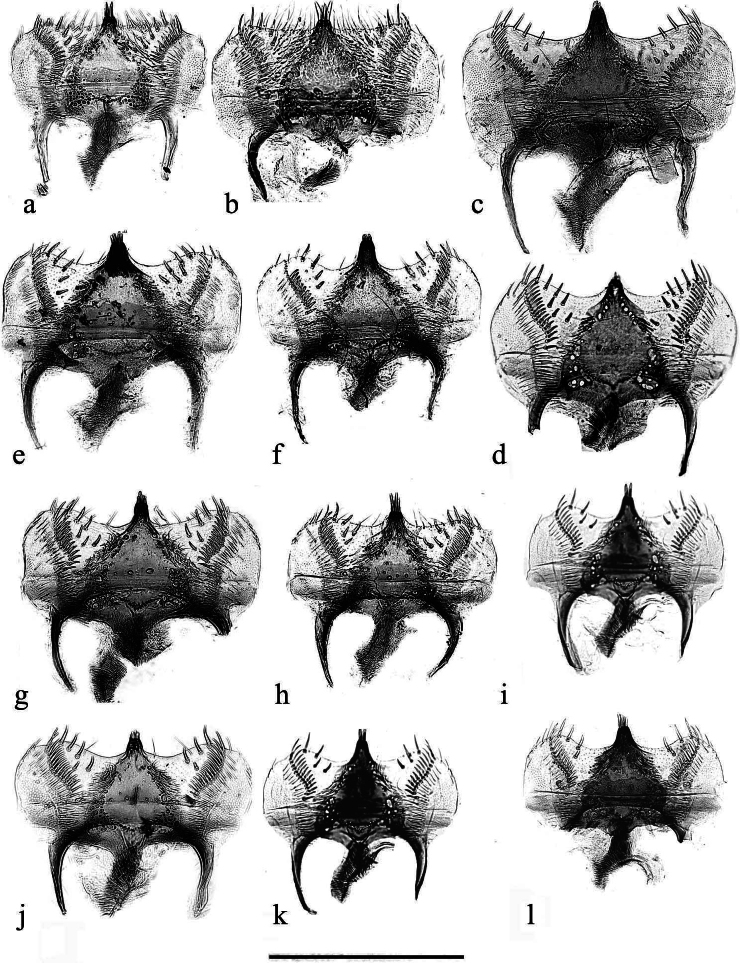
**a**–**l** epipharynxes **a**, **b***A.kraatzi*, SV **c**, **d***A.isikdagensis* paratypes, TR, Çamildere **e**, **f***A.felix* sp. nov. paratypes **g**, **h***A.bameuli* sp. nov. paratypes **i***A.alberti*? AR **j***A.krelli* sp. nov., paratype **k, l***A.alberti* sp. nov., paratypes. Scale bar: 0.5 mm.

**Figure 9.**
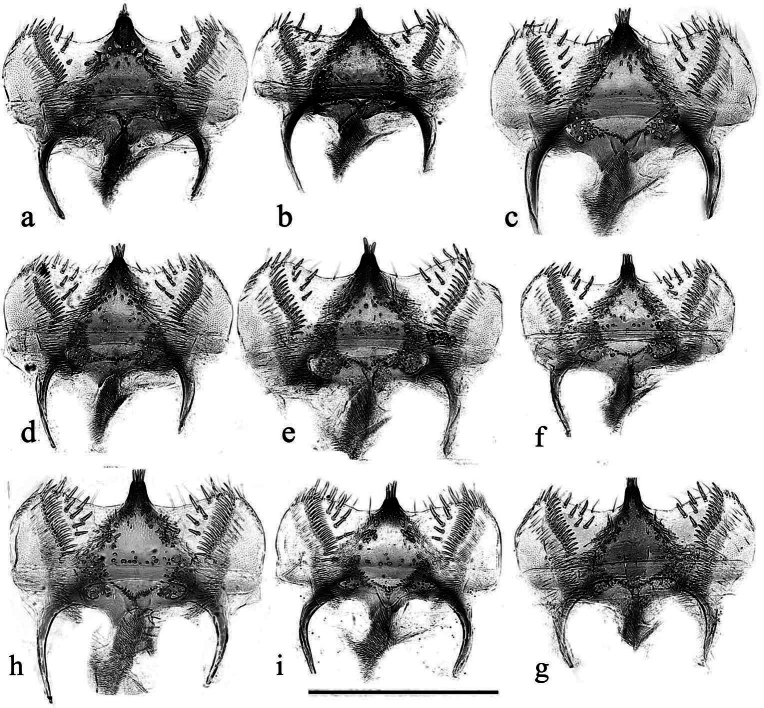
**a**–**i** epipharynxes **a**–**c***A.niger***a** SV, Tullgarn **b**, **c** GB, Hampshire, New Forest **d***A niger*? CZ, Hradec Králové **e***A.muscorum* HU, Hortobagyi **f**, **g***A.bellumgerens* sp. nov. paratypes, IT, Piano Battaglia **h**, **i***A.wilsonae* paratypes, SP **h** El Vellón **i** Manzanares el Real. Scale bar: 0.5 mm.

2. p. 245, Fig. 29c. The *A.p.plagiatus*, apparently in China (Nei Mongol), is incorrectly plotted - it should be in Mongolia.

3. p.272, *A.isikdagensis*. Fig. 8c, d is omitted from the list of figures.

4. p. 275. *A.alberti*. Before “Remarks” should be “Etymology. Named after Prof. Alberto Ballerio who collected the type material.”

5. *A.alberti* and “*ballerioi*”. In the initial drafts of this paper we (reluctantly) treated *Liothorax* as a genus and named Alberto Ballerio’s species as *A.ballerioi*. Then Prof. Ballerio cautioned that he did not regard the status of the genera/subgenera of *Aphodius* as stable and suggested using the name *alberti* to avoid future homonymy. In the event, we followed advice from two referees and treated *Liothorax* as a subgenus, so *ballerioi* was unavailable. Unfortunately, not all references were corrected, so *ballerioi* is listed in the captions to Figs 37 and 38 (p. 288) and 39A and B (pp. 289 and 290). This should be corrected to *alberti* here, as well as in any other places we still haven’t noticed.

6. In the Acknowledgements Prof. Ballerio is listed as being located in Rome, but it should be Brescia. This is correctly given in the list of Material on p. 207.

## ﻿Additional information

### Author ORCIDs

Robert B. Angus https://orcid.org/0000-0002-3860-5617

Jason F. Maté https://orcid.org/0000-0002-2505-7113

David Král https://orcid.org/0000-0002-7930-1927

### Data availability

All of the data that support the findings of this study are available in the main text.
